# The circadian phase of antenatal glucocorticoid treatment affects the risk of behavioral disorders

**DOI:** 10.1038/s41467-020-17429-5

**Published:** 2020-07-17

**Authors:** Mariana Astiz, Isabel Heyde, Mats Ingmar Fortmann, Verena Bossung, Claudia Roll, Anja Stein, Berthold Grüttner, Wolfgang Göpel, Christoph Härtel, Jonas Obleser, Henrik Oster

**Affiliations:** 1 Institute of Neurobiology, Center of Brain, Behavior and Metabolism, University of Lübeck. Marie-Curie-Straße, 23562 Lübeck, Germany; 2 Department of Pediatrics, University of Lübeck, Ratzeburger Allee 160, 23538 Lübeck, Germany; 3 Department of Women’s Health and Obstetrics University of Lübeck, Ratzeburger Allee 160, 23538 Lübeck, Germany; 4 Vestische Children’s Hospital Datteln, University Witten/Herdecke, Dr. Friedrich-Steiner-Str. 5, 45711 Datteln, Germany; 5 Department of Pediatrics I, University Hospital Essen, Hufelandstrasse 55, 45147 Essen, Germany; 6 Department of Obstetrics and Gynecology, Faculty of Medicine and University Hospital Cologne, University of Cologne, Kerpener Straße 62, 50937 Cologne, Germany; 7 Department of Pediatrics, University of Würzburg, Josef-Schneider-Strasse 2, 97080 Würzburg, Germany; 8 Department of Psychology, University of Lübeck, Maria-Goeppert-Straße 9a, 23562 Lübeck, Germany

**Keywords:** Disease model, Circadian mechanisms, Neuroendocrine diseases, Neurodevelopmental disorders

## Abstract

During pregnancy, maternal endocrine signals drive fetal development and program the offspring’s physiology. A disruption of maternal glucocorticoid (GC) homeostasis increases the child’s risk of developing psychiatric disorders later in life. We here show in mice, that the time of day of antenatal GC exposure predicts the behavioral phenotype of the adult offspring. Offspring of mothers receiving GCs out-of-phase compared to their endogenous circadian GC rhythm show elevated anxiety, impaired stress coping, and dysfunctional stress-axis regulation. The fetal circadian clock determines the vulnerability of the stress axis to GC treatment by controlling GC receptor (GR) availability in the hypothalamus. Similarly, a retrospective observational study indicates poorer stress compensatory capacity in 5-year old preterm infants whose mothers received antenatal GCs towards the evening. Our findings offer insights into the circadian physiology of feto-maternal crosstalk and assign a role to the fetal clock as a temporal gatekeeper of GC sensitivity.

## Abstract (web summary)

Antenatal glucocorticoid therapy is indicated for mothers at risk of preterm delivery. Here, the authors show that the circadian phase of antenatal glucocorticoid treatment affects the risk of behavioral disorders later in life in mice and in a retrospective observational study in human infants.

## Introduction

During fetal development, endogenous and exogenous factors program long-term physiology^1–5^. Epidemiological studies and animal experiments suggest that stress or circadian rhythm disruption (e.g. altered photoperiod, sleep deprivation) during pregnancy lead to a higher risk of developing psychiatric disorders later in life^2–7^. Interestingly, most rodent prenatal stress paradigms entail some degree of circadian disruption since the animals are manipulated during their normal rest (i.e. the light) phase. Similarly, circadian disruption itself often leads to activation of stress responses^7^.

At physiological concentrations, glucocorticoids (GCs)—mainly cortisol in humans, corticosterone (CORT) in mice—are essential drivers of fetal growth and tissue maturation^1,3^. At high concentrations maternal GCs cross the placenta and, via glucocorticoid receptor (GR) activation, regulate epigenetic processes to induce long-lasting changes in gene expression that are sustained over generations^1,8,9^. Interestingly, GCs display amongst the strongest daily rhythms in the endocrine system, peaking at the beginning of the active phase (i.e. the morning in humans and the evening in nocturnal rodents) to coordinate rhythmic functions of central and peripheral tissues^10^. Circadian rhythms in GC secretion result from a complex cooperation between the circadian master pacemaker located in the hypothalamic suprachiasmatic nucleus (SCN) and subordinate clocks along the hypothalamus-pituitary-adrenal (HPA) axis^11–13^. The SCN is entrained by external light and induces corticotropin-releasing hormone (CRH) and arginine vasopressin (AVP) release by the paraventricular nucleus of the hypothalamus (PVN). In turn, the PVN, controls the rhythmic secretion of adrenocorticotropic hormone (ACTH) from the pituitary and, consequently, GC production by the adrenal gland. Via autonomic pathways the SCN also synchronizes adrenal clocks regulating the time-of-day-dependent sensitivity of the steroidogenic machinery to ACTH stimulation^11^. This systemic coupling between the circadian and the stress system is reinforced at the molecular level by the reciprocal interaction between GR and the molecular clock machinery^14–18^.

Despite the well-established function of the circadian system in the regulation of adult physiology and behavior, possible interactions between the maternal and fetal circadian systems during pregnancy are far less understood^6,19–21^. In general, the developing fetal clocks are considered as additional subordinate oscillators dependent on maternal entrainment signals crossing the placenta^14,22,23^. Although not shown so far, the time of day when these signals reach the fetus may act, by itself, as a potential programming factor of the offspring’s physiology. However, the mechanistic examination of an interaction between the concentration of the programming signal (e.g. GCs) and the time of day when it reaches the fetus is challenging.

In human pregnancies, antenatal GC therapy is indicated for mothers at risk of preterm delivery between gestational weeks 24 and 34, without any specification of the administration time. GCs accelerate fetal lung maturation and reduce the risk of respiratory distress syndrome, the main cause of mortality in premature babies^24–26^. While substantially improving the short-term outcome of the newborns, antenatal GC treatment has also been associated with an increased vulnerability for developing stress-related disorders later in life^27,28^. Therefore, improving the benefit-to-risk ratio of antenatal GC treatment could have a lasting impact on the developmental trajectory of these infants.

We here hypothesize that the circadian phase/time of day of antenatal GC treatment might affect the risk of developing behavioral disorders later in life. Leveraging a mouse model and supplementing it translationally with human infant data, we show that maternal exposure to antenatal GCs out-of-phase compared to the physiological GC rhythm has more profound effects on programming the offspring’s/infant’s behavior than when given in-phase. This temporal difference depends on the fetal circadian clock.

## Results

### The time of antenatal GCs predicts behavior in the offspring

**Fig. 1:**
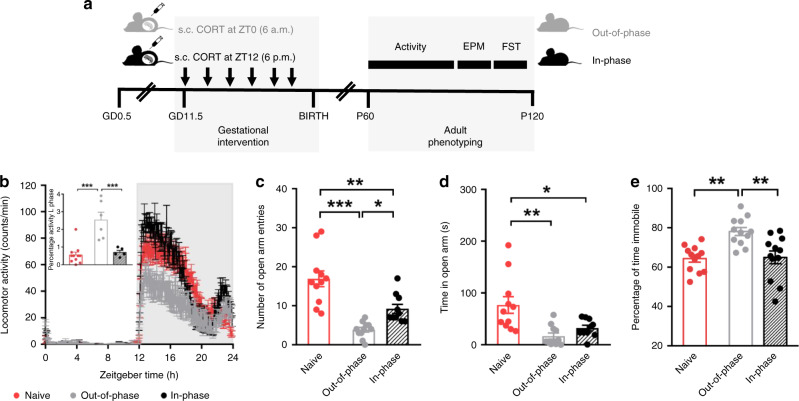
The time of maternal exposure to GCs predicts the offspring’s behavior. **a** Scheme of the gestational intervention by subcutaneous (s.c.) injections of corticosterone (CORT) either at Zeitgeber time 0 (ZT0, 6 a.m) or at ZT12 (6 p.m). Long-term effects on the offspring’s circadian stress system were assessed at behavioral level from postnatal day 60 (P60) to P120. To confirm the effect of the preterm manipulation itself (including handling and CORT administration from gestational day (GD) 11.5 until birth), we also included a naive group of adult mice (always in red) whose mothers were left undisturbed during their whole pregnancy. **b** Circadian pattern of locomotor activity in 12-h light:12-h dark cycle conditions and percentage of activity during the light (L) phase (inset). The running-wheel counts/min of 10 days were averaged for each individual animal (naive *n* = 11, out-of-phase *n* = 6, in-phase *n* = 5). **c** Number of open arm entries in the elevated plus maze (EPM) (naive *n* = 11, out-of-phase *n* = 11, in-phase *n* = 10). **d** Time spent in the open arm of the EPM (naive *n* = 11, out-of-phase *n* = 11, in-phase *n* = 10). **e** Percentage of time spent immobile in the forced swim test (FST) (*n* = 12 for all groups). All behavioral tests were performed once. All data are expressed as means ± SEM. Data in **b** (inset), **c**–**e** were analyzed by one-way ANOVA; **b** *F*(2,19) = 17.60, *p* < 0.0001; **c** *F*(2,29) = 22.33, *p* < 0.0001; **d** *F*(2,29) = 8.79, *p* = 0.001; **e** *F*(2,33) = 9.78, *p* = 0.0005; followed by Sidak’s multi-comparison test. *p < 0.05, **p < 0.01, ***p < 0.001. Source data are provided as a Source Data file.

In order to assess whether the time of maternal GC exposure influences the offspring’s behavior in the long term, we injected pregnant wild-type mice daily with corticosterone (CORT) at two different times of the day during late pregnancy (gestational day (GD) 11.5 to birth) (Fig. 1a). One group of pregnant mice was subcutaneously (s.c.) injected at the beginning of the light phase (ZT0, Zeitgeber time 0, referring to the time of day when the lights were switched on in the animal facility, in our case 6 a.m.). A second group of pregnant mice was injected at the end of the light phase (ZT12, i.e. the time of day when the light was switched off in the animal facility, in our case 6 p.m.) (Fig. 1a). As in most nocturnal species, GC blood levels in unstressed mice peak around ZT12. Therefore, by injecting CORT at ZT0, the maternal exposure occurred out-of-phase compared to the physiological diurnal GC rhythm (out-of-phase group; always shown in gray). Similarly, by injecting CORT at ZT12 the maternal exposure occurred in phase with the physiological diurnal GC rhythm (in-phase group; always shown in black) (Fig. 1a). The offspring from treated mothers were weaned at postnatal day 21 and left undisturbed until tested for stress-related behavioral responses starting at the age of 60 days (P60). Of note, the only experimental difference between the two treatment groups was the timing of GC exposure. To confirm the effect of the preterm manipulation itself (including handling and GC administration) on stress system programming, for every experiment we also included a naive group of adult mice (always shown in red) whose mothers were left undisturbed during their whole pregnancy, weaned at postnatal day 21 and left undisturbed until tested.

The offspring of the out-of-phase group showed disturbed locomotor activity patterns with increased activity during the light/rest phase and decreased activity during the dark/active phase (Fig. 1b). When tested in the elevated plus maze (EPM), out-of-phase offspring showed less open arm entries and spent less time in the open arms, suggesting higher levels of anxiety-like behavior (Fig. 1c, d and Supplementary Fig. 1a). In line with this, they spent more time immobile in the forced swim test (FST) indicating a reduced resistance to stress (Fig. 1e and Supplementary Fig. 1b) compared to in-phase offspring. Comparing these behavioral outcomes with the naive group confirmed that the prenatal manipulation had an effect by itself; however, the CORT exposure out-of-phase showed stronger programming effects.

**Fig. 2:**
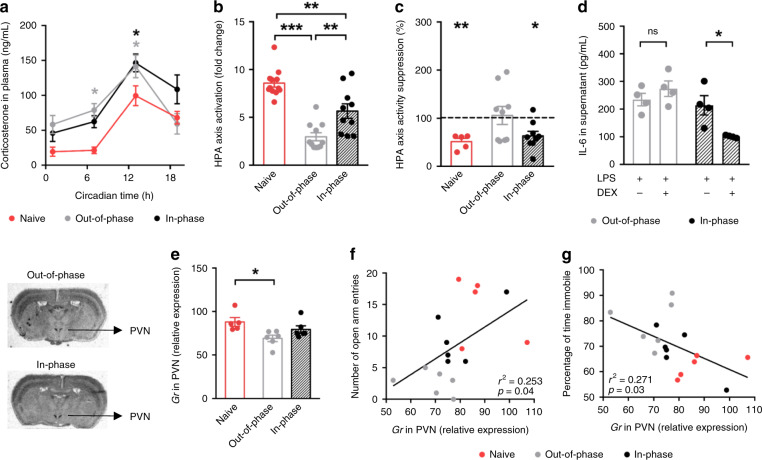
The time of maternal exposure to GCs predicts the offspring’s HPA axis function. Scheme of the gestational intervention is shown in Fig. 1a. Long-term effects on the offspring’s circadian stress system were assessed at systemic (**a**–**d**) and molecular (**e**–**g**) levels. To confirm the effect of the preterm manipulation itself (handling and corticosterone (CORT) administration from gestational day (GD) 11.5 until birth) on hypothalamic-pituitary-adrenal (HPA) axis function, we included a naive group of adult mice (in red) whose mothers were left undisturbed during their whole pregnancy. **a** Circadian profile of CORT in plasma in constant darkness, (naive ZT1 *n* = 7, ZT7 *n* = 7, ZT13 *n* = 10 and ZT19 *n* = 8; out-of-phase ZT1 *n* = 9, ZT7 *n* = 7, ZT13 *n* = 11 and ZT19 *n* = 8; in-phase ZT1 *n* = 11, ZT7 *n* = 10, ZT13 *n* = 11, and ZT19 *n* = 7). Gray or black asterisks indicate differences between the naive group and out-of-phase or in-phase, respectively. **b** HPA axis activation as fold change of CORT in plasma before and after FST (naive *n* = 12, out-of-phase *n* = 11, in-phase *n* = 10). **c** Dexamethasone (DEX) suppression of CORT production expressed as percentage of vehicle treatment (dotted line) (naive *n* = 5, out-of-phase *n* = 9, in-phase *n* = 9). CORT was measured in duplicates. **d** Glucocorticoid receptor (GR) sensitivity assessed by lipopolysaccharide (LPS)-induced interleukin −6 (IL−6) production in whole blood with/without DEX (*n* = 4/treatment/group). IL-6 measurements were run in duplicates. **e** *Gr* relative mRNA expression in the hypothalamic paraventricular nuclei (PVN) by in situ hybridization (three sections were quantified and averaged/mice, naive *n* = 5, out-of-phase *n* = 6, in-phase *n* = 6), on the left, representative images show the quantified region. **f**, **g** Linear correlation between, *Gr* expression in PVN and number of open arm entries (**f**) and the percentage of time immobile (**g**). Data in **b**, **e** were analyzed by one-way **b** *F*(2,30) = 28.84, *p* < 0.0001; **e** *F*(2,14) = 4.85, *p* = 0.0251) and data in **a** by two-way ANOVA (treatment effect *F*(2,93) = 9.28, *p* = 0.0002) followed by Sidak’s multi-comparison test. Data in **c** were analyzed by two-sided unpaired *t*-test for each group independently (Naive VEH vs DEX, *t* = 3.881, df = 9, *p* = 0.0037; in phase vehicle (VEH) vs DEX, *t* = 2.26, df = 16, *p* = 0.0382). Data in **d** were analyzed by two-sided Mann–Whitney test for each group (*p* = 0.0286). *p < 0.05, **p < 0.01, ***p < 0.001. Source data are provided as a Source Data file.

Alterations in stress and anxiety-related behaviors are often associated with a dysfunction of the HPA axis^29^. Thus, we further characterized HPA axis regulation in the three groups. Plasma GC levels and GC metabolites in feces were overall higher in prenatally GC exposed offspring compared to naive mice (Fig. 2a and Supplementary Figs. 1c–e), especially during the rest phase (Circadian time 0–12), regardless of the timing of maternal treatment. However, when the offspring’s stress system was challenged by 6 min of forced swimming (FST), the HPA axis’ response (showed as a positive fold change of GC levels in plasma before and after the stress event) was significantly poorer in the out-of-phase group compared to the in-phase group (Fig. 2b). From the comparison with the naive group, we can confirm that the prenatal manipulation had an effect on HPA axis function programming regardless of the manipulation time; however, it was stronger when the maternal GC exposure was out-of-phase.

Elevated GC levels during the rest phase (Fig. 2a), combined with a poor acute stress response (Fig. 2b), suggest an impairment of HPA axis reactivity which depends on the ability of CORT to inhibit its own production by a negative feedback mechanism. We performed a dexamethasone (DEX) suppression test to assess this possibility. Mice received a single 0.9% saline or DEX injection (100 μg/kg body weight in 0.9% saline, intraperitoneal injection) at ZT8 (8 h after lights on) and blood samples were taken 6 h later (at the time of the circadian CORT peak). DEX, as a synthetic GC analogue, suppresses CORT production through the inhibition of CRH and ACTH release from the hypothalamic PVN and the pituitary gland, respectively. DEX had a significant supressive effect (around 40% compared with saline injection, dotted line) on CORT production in the in-phase group (similar to its effect in naive mice). However, DEX did not significantly suppress HPA axis function in the out-of-phase group, in line with an impairment in the negative feedback mechanism in these mice (Fig. 2c). At the molecular level, HPA axis regulation relies on GR signaling. We tested the sensitivity of GR by an ex vivo assay in which peripheral blood from the offspring was incubated with lipopolysaccharide (LPS) to activate the secretion of interleukin 6 (IL-6). Co-incubation with DEX inhibits IL-6 release through the activation of GR. Thus, the degree of suppression of IL-6 production by DEX is an indication of GR sensitivity. While GR sensitivity was conserved for the in-phase group, offspring of mothers injected out-of-phase showed a significantly reduced GR sensitivity (Fig. 2d). In addition to the peripheral reduction in GR sensitivity, which could also occur at central levels, we detected a down-regulation of *Gr* mRNA at HPA axis regulatory centers such as the hypothalamic PVN and the dentate gyrus (DG) of the hippocampus (Fig. 2e and Supplementary Fig. 1f–h), further supporting an impaired negative feedback. Moreover, a significant linear correlation between the behavioral outcomes (from Fig. 1c, e) and *Gr* expression in the PVN was seen (Fig. 2f, g).

In summary, these data show that maternal exposure to CORT out-of-phase with regard to the maternal GC rhythm has stronger programming effects on the offspring’s behavior than the same dose, but given in-phase. This difference in behavior is likely explained by a difference in the offspring’s HPA axis regulation found at systemic and molecular levels, as shown previously by others^2,30–32^.

The established model of GC-dependent prenatal programming states that antenatal treatment increases maternal concentrations of GCs that cross the placenta and, via GR in fetal target tissues, activate the epigenetic machinery responsible for long-lasting changes in gene expression that are sustained over generations^1,8,9,33^. We reasoned that the influence of the time of maternal exposure on the offspring’s HPA axis programming could be caused by either a differential amount of CORT reaching fetal tissues or a differential GC sensitivity of fetal tissues at different times of the day.

### Fetal hypothalamic GR activity is diurnally regulated

**Fig. 3:**
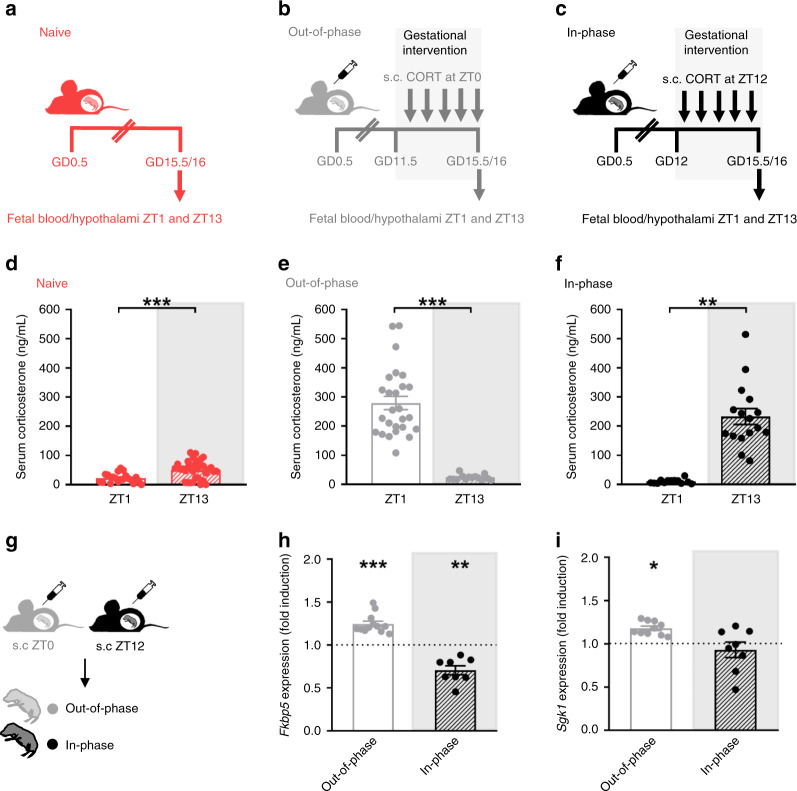
Fetal hypothalamic GR transcriptional activity is diurnally regulated. **a**–**c** Scheme of the prenatal timed corticosterone (CORT) intervention. Mothers were left undisturbed (**a**) or injected subcutaneously (s.c) with CORT at Zeitgeber time 0 (ZT0, 6 a.m) (**b**) or at ZT12 (6 p.m) (**c**) from gestational day (GD 11.5). On GD 15.5–16.5 fetal blood and hypothalami were collected at ZT1 and ZT13. **d** Corticosterone levels in fetal serum from undisturbed mothers at ZT1 and ZT13 (*n* = 20 ZT1 and *n* = 33 ZT13). **e** Corticosterone in fetal serum from mothers injected at ZT0 (out-of-phase, *n* = 26 ZT1 and *n* = 14 ZT13) and **f** Corticosterone in fetal serum from mothers injected at ZT12 (in-phase, *n* = 13 ZT1 and *n* = 16 ZT13). Corticosterone measurements were done in duplicates. **g** Scheme of the prenatal timed CORT intervention, the fetal hypothalamus was dissected 1 h after maternal injection. **h**, **i** mRNA relative expression of the glucocorticoid receptor (GR) targets FK506 binding protein 5 (*Fkbp5*) (h) and Serum glucocorticoid-regulated kinase 1 (*Sgk1*) (i) in the hypothalamus of fetus from wild-type mothers. For **h** and **i** gene expression is shown as fold induction after CORT treatment (at ZT0 or ZT12) relative to naive fetus (dotted line) (*n* = 9 naive ZT1, *n* = 12 out-of-phase ZT1, *n* = 12 naive ZT13, *n* = 8 in-phase ZT13). qPCRs for gene expression were run in duplicates. Data are expressed as means ± SEM. Differences of corticosterone levels at both time points (**d**–**f**) and fold induction after CORT injection (**h**, **i**) were analyzed by two-sided unpaired *t*-test **d** *t* = 3.89, df=51, *p* = 0.0003; **e** *t* = 8.15, df=38, *p* < 0.0001; **f** *t* = 7.313, df=27, *p* < 0.0001; **h** *Fkbp5*: Naive ZT1 vs out-of-phase ZT1 *t* = 3.44, df=19, *p* = 0.0027; Naive ZT13 vs in-phase ZT13 *t* = 4.39, df=18, *p* = 00003. **i** *Sgk1*: Naive ZT1 vs out-of-phase ZT1 *t* = 3.29, df=17, *p* = 0.0043. *p < 0.05, **p < 0.01, ***p < 0.001. Source data are provided as a Source Data file.

As shown before by others^21^, in naive conditions circadian CORT levels are 10 times higher in the mother than in the fetus due to CORT inactivation by placental 11β-hydroxysteroid dehydrogenase type-2 (11β-HSD2)^34,35^ (Supplementary Fig. 2a, b). However, in our paradigm, 1 h after injection (at ZT1 or ZT13), we noted a sharp increase of fetal and maternal CORT levels in blood independent of the treatment time (Fig. 3d–f and Supplementary Fig. 2c–e, respectively). This indicates that CORT levels in the mother are high enough to saturate the placental 11β-HSD2, resulting in similarly high levels of CORT reaching fetal tissues at both time points.

Therefore, the influence of the time of maternal exposure relates to a time-dependent sensitivity of fetal tissues to CORT. We tested this possibility by assessing the diurnal difference of GR transcriptional activity in the fetal hypothalamus, which expresses high levels of GR during this period of development^1,2,23,36^. One hour after CORT injections to the mother (at ZT0 or ZT12) on gestational days (GDs) 15.5 and 16.5 (Fig. 3a–c) the expression levels of two early and GR-specific target genes^37^, *Fkbp5* (FK506 binding protein 5) and *Sgk1* (serum glucocorticoid-regulated kinase 1) were elevated only after the out-of-phase, but not the in-phase CORT injection (Fig. 3g–i) compared to the naive condition (dotted line). These data supported our model of a time-of-day-dependent regulation of GR transcriptional activity in the fetus.

Such time-dependent regulation of GR activation could result from an interaction between the GC-GR signaling pathway and the circadian clock. The cellular circadian clockwork is based on a set of clock genes including *Bmal1* (brain and muscle aryl hydrocarbon receptor nuclear translocator-like 1), *Per1*/*2* (period 1/2), and *RevErbα* (reverse erythroblastoma alpha) organized in a system of interlocked transcriptional-translational feedback loops^38–41^. Time-of-day information is translated into physiological signals through rhythmic regulation of downstream clock-controlled genes^42,43^. If the fetal clock was involved in regulating time-of-day-dependent GR activity, we would expect that, by genetically removing the fetal clock, the activation of GC targets after maternal injection would become independent of exposure time. However, the functionality of the fetal clock during development is not clear^44–46^. Rhythmic expression of clock genes has been detected as early as GD13^47^, but it is still not known whether these oscillations are endogenously generated or driven by maternal rhythmic signals, and we sought to investigate this first.

### Fetal hypothalamic rhythms of GR and REVERBα are anti-phasic

**Fig. 4:**
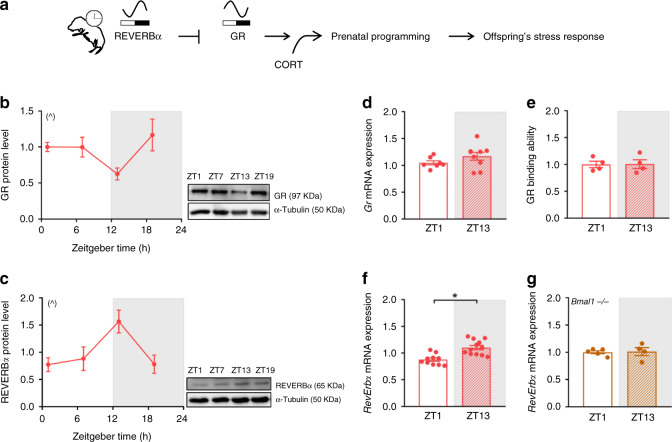
In the fetal hypothalamus, diurnal rhythms of GR and REVERBα are anti-phasic. **a** Working model of reverse erythroblastoma alpha (REVERBα)-mediated regulation of rhythmic glucocorticoid receptor (GR) protein amount and prenatal programming of the offspring’s stress response. **b**, **c** Circadian profile of GR (**b**) and REVERBα (**c**) protein in fetal hypothalami obtained from naive mothers euthanized every 6 h (at Zeitgeber time (ZT) 1, 7, 13 and 19) during gestational day (GD) 15.5 to 16.5, (GR *n* = 6 ZT1, *n* = 5 ZT7, *n* = 5 ZT13, *n* = 5 ZT19 and REVERBα *n* = 6 for all time points). Representative Western blot results are shown; samples were run in duplicates and α-Tubulin was used as loading control. **d** *Gr* mRNA expression in fetal hypothalami from naive pregnant mothers euthanized at ZT1 (*n* = 7) and ZT13 (*n* = 8). **e** GR binding to glucocorticoid receptor binding elements (*GRE*s) assessed in fetal hypothalami from naive pregnant mothers (*n* = 4 for both time points). **f**, **g** *RevErbα* mRNA expression in wild-type (*n* = 10 ZT1 and *n* = 12 ZT13) (**f**) and brain and muscle aryl hydrocarbon receptor nuclear translocator-like 1 (*Bmal1*^*−/−*^) (**g**) fetal hypothalamus (*n* = 5 ZT1 and *n* = 4 ZT13) from naive mothers. qPCRs for gene expression and GR binding assays were run in duplicates. All data are expressed as means ± SEM. In **b**, **c** significant circadian rhythmicity was determined by CircWave v1.4 which fits a standard cosine function to an averaged de-trended data assuming a period of 24 h, a *p* < 0.05 was considered significant (^, **b** *r*^2^ = 0.37, *p* = 0.05; **c** *r*^2^ = 0.38, *p* = 0.028). In **f** data were analyzed by two-sided unpaired *t*-test (**f** *t* = 4.35, df=20, *p = 0.0003). Source data are provided as a Source Data file.

If the fetal clock would differentially regulate GR transcriptional activity depending on the time of day, four levels of fetal clock-dependent control appear possible: rhythms in GR expression, in GR availability, in GR activation or in GR binding to GC regulatory elements (GREs). Amongst the GR-regulating clock proteins described so far^12,15–18^, REVERBα would be a good candidate because it is the earliest clock protein to oscillate in the fetal hypothalamus^48^ and interacts with the heat shock protein HSP90 and GR in the cytosol to reduce GR stability, its translocation to the nucleus and subsequent activity (Fig. 4a)^17^. Diurnal rhythms of GR and REVERBα were anti-phasic at the protein level in the fetal hypothalamus (Fig. 4b, c). In contrast, no diurnal regulation of *Gr* was seen at mRNA levels nor for GR’s ability to bind DNA in naive conditions (Fig. 4d, e). With the exception of *Gr* mRNA levels (Supplementary Fig. 3a), these diurnal differences were similar even after CORT treatment (Supplementary Fig. 3b, c).

### Fetal clocks control GC sensitivity by rhythmic REVERBα

**Fig. 5:**
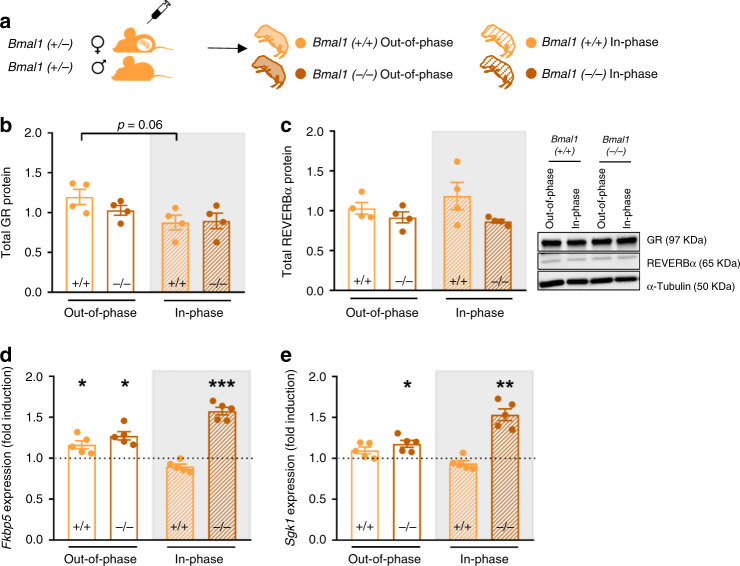
The fetal hypothalamic clock controls GC sensitivity through expression of REVERBα. **a** Brain and muscle aryl hydrocarbon receptor nuclear translocator-like 1 (*Bmal1*^−/−^ and *Bmal1*^*+/+*^), clock deficient and wild-type fetus, respectively were obtained from mating *Bmal1*^*+/−*^ heterozygous couples aiming to test the clock-dependent induction of hypothalamic glucocorticoid receptor (GR) targets after corticosterone (CORT) injection. **b**, **c** GR and Reverse erythroblastoma alpha (REVERBα) protein in fetal hypothalami (out-of-phase Zeitgeber time (ZT)1 *Bmal1*^+/+^ and ^−/−^, in-phase ZT13 *Bmal1*^+/+^ and ^−/−^, *n* = 4 for all groups). Representative Western blot results are shown; samples were run in duplicates and α-Tubulin was used as loading control. **d**, **e** mRNA relative expression of the GR targets FK506 binding protein 5 (*Fkbp5*) and Serum glucocorticoid-regulated kinase 1 (*Sgk1*) in the hypothalami of fetus from CORT injected *Bmal1*^−/+^ mothers mated to males of the same genotype. The dotted line represents the expression levels of fetus from naive mothers of the respective genotype and time point (naive and out-of-phase ZT1 *Bmal1*^+/+^ and ^−/−^, naive and in-phase ZT13 *Bmal1*^+/+^ and ^−/−^, *n* = 5 for all groups). qPCRs for gene expression were run in duplicates. All data are expressed as means ± SEM. In **b** data were analyzed by two-sided Mann–Whitney test (**b**) out-of-phase ZT1 vs out-of-phase ZT13, *p* = 0.06. In **d**, **e** data were analyzed by two-sided unpaired *t*-test (**d** *Fkbp5*: naive ZT1^+/+^ vs out-of-phase ZT1^+/+^, *t* = 2.22, df=8, *p* = 0.05; naive ZT1^−/−^ vs out-of-phase ZT1 ^−/−^, *t* = 2.33, df=8, *p* = 0.04; naive ZT13^−/−^ vs in-phase ZT13^−/−^, *t* = 6.53, df=8, *p* = 0.0002; **e** *Sgk1*: naive ZT1^−/−^ vs out-of-phase ZT1^−/−^, *t* = 2.80, df=8, *p* = 0.02; naive ZT13^−/−^ vs in-phase ZT13^−/−^, *t* = 4.23, df = 8, *p* = 0.002). *p < 0.05, **p < 0.01, ***p < 0.001. Source data are provided as a Source Data file.

To assess whether REVERBα could be the clock protein controlling GR levels, and by that the diurnal transcriptional activity in the fetal hypothalamus, we first confirmed that the rhythmic expression of *RevErbα* seen in wild-types (Fig. 4f) was lost in clock-deficient *Bmal1*^−/−^ fetuses (Fig. 4g). We next bred *Bmal1*^+/−^ heterozygous couples and compared the diurnal control of GR transcriptional activity between *Bmal1*^*+/+*^ and (clock-deficient) *Bmal1*^−/−^ fetuses (Fig. 5a). In the absence of fetal *Bmal1*, GR and REVERBα protein levels were comparable at ZT1 and ZT13 (Fig. 5b, c) and, consequently, the expression of the GR target genes *Fkbp5* and *Sgk1* was induced not only after the injection at ZT0 (out-of-phase), but also after injection at ZT12 (in-phase), showing a loss of time-dependent GR transcriptional activity regulation (Fig. 5d, e).

In summary, these experiments suggest that the circadian rhythm of the clock protein REVERBα could be responsible for reducing the amount of GR at ZT13 in the fetal hypothalamus, which would explain the time-of-day dependence of the induction of GR targets after maternal GC treatment. When high levels of CORT reach the fetus out-of-phase with the mother’s rhythm, more GR is available resulting in an increased sensitivity of the fetal stress axis to be programmed.

It could still be possible that the maternal clock would be somehow involved in this time-of-day-dependent regulation of GR transcriptional activity. If so, one would expect that by genetically removing the maternal clock GR transcriptional activity in the fetal hypothalamus after CORT would be independent of the time of injection. To test this, we bred clock-deficient *Per1/2* double mutant^49^ females to wild-type (WT) males (Supplementary Fig. 4a). Similar to what we had observed in wild-types (Fig. 3h, i), the expression of GR targets was elevated in the fetal hypothalamus only after out-of-phase injection (Supplementary Fig. 4b, c), suggesting that the maternal clock has little impact on the time-dependent activation of GR.

### Out-of-phase GC exposure alters behavior in preterm infants

**Fig. 6:**
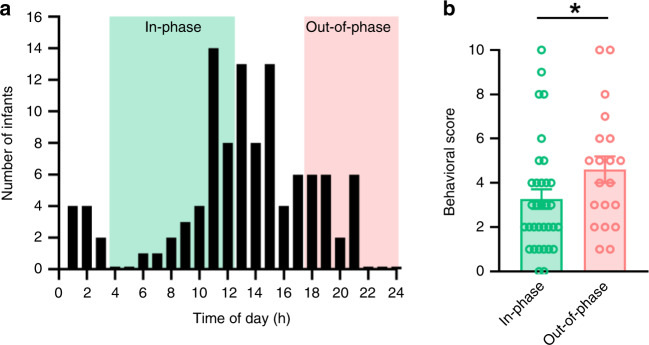
Preterm infants who received antenatal GCs out-of-phase show altered behavior. **a** Distribution of the number of 5-year-old preterm infants included in the analysis. Data were collected in three centers included in the German Neonatal Network; Lübeck, Cologne and Essen. Depending on the difference (in hours) between the time of maternal physiological cortisol peak (estimated at 8:00,^74^) and the time of antenatal betamethasone injection, preterm infants were divided into two groups; the in-phase group, injected between 4:00 and 12:00 (green shading) and the out-of-phase group, injected between 18:00 and 0:00 (pink shading). **b** A compound behavioral score (0 to 10) was used to quantify stress compensation capacity, infants with high score are reported to have more features indicating poor stress compensation capacity^51^. In total, 53 behavioral scores of 5-year-old preterm infants from the three centers were included in the in-phase (*n* = 33) and out-of-phase groups (*n* = 20) (i.e. 54 children out of *n* = 107 belong to the middle group whose mothers were injected from 12:00 to 18:00 and from 0:00 to 4:00). Data are expressed as means ± SEM; *indicates *p* = 0.031 [*β* = .65, *t* (49) = 2.18, partial *η*^2^ = 0.08; two-sided, uncorrected *p*-value for parameter estimate resulting from an ordinary-least squares linear regression model predicting the behavioral score from in-phase/out-of-phase antenatal GCs while adjusting for mode of delivery, the only confounding factor; see Table 1]. Note that this parameter estimates for the out-of-phase/in-phase difference holds when including data from all preterm infants into the model, including the additional *N* = 54 from the middle group [two-sided, uncorrected *p* = 0.035, *β* = .63, *t* (101) = 2.14, partial *η*^2^ = 0.05; identical model specifications]. Source data are provided as a Source Data file.

As mentioned above, in human pregnancies, antenatal GC therapy is indicated since the early 1990s to mothers at risk of preterm delivery. Despite being extremely beneficial in the short-term, antenatal GCs increase the vulnerability to develop stress-related disorders later in life^27,28^. We performed a retrospective observational analysis to determine whether the time of maternal GC exposure was a predictor of the behavioral outcome of 5-year old preterm infants from a population-based cohort of the German Neonatal Network (GNN). 107 preterm infants with complete follow-up from three participating centers—Lübeck, Cologne, and Essen—were included. Based on the time of maternal treatment, preterm infants were stratified in two groups, in-phase (*n* = 33) and out-of-phase (*n* = 20) with the maternal cortisol rhythm (Fig. 6a, Table 1). The nondescript middle group (*n* = 54) was excluded. A compound behavioral score was used to quantify stress compensation capacity for each child, according to a questionnaire based on the German Health Interview and Examination Survey for Children and Adolescents^50,51^ (Fig. 6b). A simple paired comparison showed that preterm infants administered antenatally with GCs out-of-phase have higher parent-rated scores (i.e., a weaker stress compensatory capacity; Cohen’s d = 0.52, or a 40% score increase) than preterm infants administered in-phase (Fig. 6b).

**Table 1:** Cohort description.

	In-phase	Out-of-phase
Group size	33	20
Gestational age (weeks)	27.3 ± 2	26.6 ± 2
Birth weight (g)	995 ± 258	915 ± 259
Small for gestational age (%)	12.1	20
Mode of delivery		
Spontaneous	3	1
Planned C-section	29	13
Emergency C-section	1	6
Gender (m/f)	15/18	12/18

In total, 53 5-year-old preterm infants from Lübeck, Cologne and Essen were included in the in-phase and out-of-phase groups (i.e., 54 out of *n* = 107 children fell into a nondescript middle group). Data describing both groups are expressed as means ± SD. No statistical differences were found between the groups when analyzed by two-sided Mann–Whitney test for confounder variables such as birth weight, gestational age, small for gestational age and gender. Note however the potentially confounding association of mode of delivery with in-phase/out-of-phase GCs administration (*χ*^2^(2) = 7.96, *p* = 0.02). Accordingly, mode of delivery was included in the statistical model reported in Fig. 6b. Source data are provided as a Source Data file.

A linear regression model predicting the behavioral score from in-phase/out-of-phase antenatal GCs while adjusting for the confounder mode of delivery (Table 1) confirmed this relationship. Notably, the difference in behavioral scores between in-phase and out-of-phase preterm infants held when including data from all 107 children (i.e., including infants from the nondescript middle group) in the model (*p* = 0.035, *β* = 0.63, *t* (101) = 2.14, partial *η*^*2*^ = 0.05). When dissecting the results for specific questionnaire items (Supplementary Fig. 5 and Supplementary Table 1), preterm infants whose mothers received antenatal GCs out-of-phase (i.e. late during the day) proved more quick-tempered than preterm infants who received antenatal GC in-phase (Cohen’s *d* = 0.72, *p* = 0.014). Although not statistically significant, infants from the out-of-phase group tended to be more hyperactive (*d* = 0.28, *p* = 0.33), nervous (*d* = 0.28, *p* = 0.33) and anxious (*d* = 0.08, *p* = 0.8), as well as afraid of new situations easily losing self-confidence (*d* = 0.51, *p* = 0.8). In summary, these retrospective data corroborate our experimental mouse data where out-of-phase GC exposure during pregnancy correlates with impaired stress-coping behavior later in life.

## Discussion

Leveraging a mouse model, the current study sought to show that maternal exposure to antenatal GCs out-of-phase compared to the physiological diurnal GC rhythm has more profound effects on programming the offspring’s behavior than when given in-phase. This temporal difference was found to depend on the fetal circadian clock, and it resonated with observational data from a longitudinal study on antenatal GC administration in humans.

Coupling between the circadian clock and stress regulatory systems is achieved by a complex crosstalk, from the systemic to the molecular level^12–14^. Adaptations of both systems during pregnancy are essential to maintain physiological homeostasis in response to external stimuli and internal demands to coordinate fetal development^6,21^. Most of the current knowledge on circadian system function during pregnancy derives from experiments in rodents. The fetal circadian system develops gradually during pregnancy while being entrained (and programmed) by maternal body temperature, feeding and hormonal cycles^19,20^. Chronodisruption induced by, e.g., altering photoperiod during pregnancy is able to program long-term metabolism^52^ and behavior^53^ in a very similar way as prenatal stress^2,30,52,54–56^. Most prenatal stress paradigms entail certain degrees of circadian disruption because animals are subjected to different manipulations during their normal rest/light phase^57^. Therefore, the mechanistic examination of an interaction between the concentration of the programming signal (e.g. GCs) and the time of day when it reaches the fetus is rather difficult to assess. Our experimental paradigm allowed us to overcome this issue providing evidence that the circadian timing of antenatal GC administration is a predictor of stress-related behavioral phenotypes later in life.

In our mouse model, maternal exposure to CORT out-of-phase compared to the endogenous GC rhythm led to a disturbed 24-h activity pattern, increased anxiety and reduced resistance to stress compared to offspring of mothers exposed to the same dose but in-phase with GC rhythm. We have explained this difference in the offspring’s behavior by a differential regulation of the HPA axis. On the molecular level, HPA axis regulation relies on GR signaling. GCs bind two intracellular receptors, MR (mineralocorticoid receptor) and GR. Because MR has a high affinity for GCs, it is constitutively active, relaying tonic information. GRs, in turn, are only activated at peak GC concentrations, conveying phasic responses, e.g. at circadian peaks around the beginning of the active phase or during stress situations^31^. Thus, according to the concentration of CORT we were able to measure in fetal blood (comparable with GC peak concentrations) and the fact thatt MR shows low expression in the hypothalamus, we considered GC-GR signaling to be essential in determining the effects of antenatal GC exposure^58^. In line with this, offspring whose mothers were injected out-of-phase showed reduced GR sensitivity and down-regulation of *Gr* mRNA in the PVN, which may explain the observed dysfunctional HPA axis regulation. As suggested by others, high GC levels reaching fetal tissues induce epigenetic modifications on key genes involved in HPA axis regulation, which are then responsible for the long-term effects^9,32,33,57^. Differential DNA methylation of *Crh*^59^, *Avp*^60^, *GR*^61^, and *Fkbp5*^62^ was found in prenatally stressed rodents compromising circadian and stress-induced function of the HPA axis.

Interestingly, our results reveal that the fetus’ stress-circadian system is more susceptible to be programmed when CORT is given in the maternal rest phase, indicating the presence of a circadian gating mechanism. The molecular clockwork is present in essentially all cells, but the nature of clock-controlled genes is highly tissue-specific^63^. In order to generate physiologically meaningful rhythms, these tissue clocks have to be properly aligned with each other and the external time. Therefore, the mammalian circadian system is organized in a hierarchical manner directed by a master pacemaker in the SCN. The response of peripheral tissues to systemic signals is often different at different times of the day, i.e. it shows circadian gating, and depends on local clocks^64^.

Recent work from our lab and others reported rhythmic expression of clock genes in fetal tissues (SCN, liver and kidney) in vitro at an early gestational day (GD13)^44,47,65^. However, it is still under discussion whether these oscillations take place in vivo and to which extent they are independent from maternal entrainment signals^45,46^. It has been proposed that the fetal circadian clock could be entrained by maternal rhythmic signals similar to peripheral tissue clocks in adults^23^. Thus, we hypothesized that embryo clocks could gate the response to systemic maternal signals. We reasoned that the influence of the time of maternal exposure to CORT on the offspring’s HPA axis programming could be due either to a differential amounts of CORT reaching fetal tissues or a differential sensitivity of fetal tissues at different times of the day.

In our experimental model CORT levels rose sharply in the fetal blood after maternal injection independent of treatment time. However, two GR target genes were induced only after an out-of-phase injection, indicating that a time-of-day-dependent regulation of GR transcriptional activity was, in fact, gated at the level of the fetal tissue.

GR expression and activity are regulated by several clock proteins in adult tissues^12,15–18^. REVERBα was recently reported as the earliest clock protein oscillating with a 24-h period in the fetal hypothalamus^48^. It interacts with the heat shock protein HSP90 and GR in the cytosol, reducing GR stability^17^. We found that in the fetal hypothalamus, diurnal rhythms of GR and REVERBα are anti-phasic at the protein level and absent in clock-deficient fetuses. In the latter, time-of-day-dependent regulation of GR transcriptional activity was also lost. Together, these data suggest that the fetal clock acts as a gatekeeper of GC sensitivity of the fetal hypothalamus, time-dependently modulating the programming effects of antenatal GC treatment and determining the risk for long-lasting adverse behavioral effects. However, our data cannot fully exclude a role of the maternal clock in the long-term GC programming since several other factors related to the maternal arrhythmic behavior (e.g. nursing, feeding, activity) could play a programming role^49^.

In the human observational study, very similar to what we observed in mice, synthetic GCs (betamethasone) injected out-of-phase in mothers at risk of preterm delivery were associated with an increased susceptibility to behavioral impairment in 5-year old preterm infants. Despite being one of the most important antenatal therapies to improve the outcome of the newborns, our data suggests that the negative long-term effects of antenatal GCs could be improved by injecting GCs at the right time. This possibility would be of main importance since, due to the lack of good predictors for preterm delivery, a percentage of pregnant women receive antenatal GCs, but then the babies are born full term. Although the translational potential of our findings seems promising, there are several limitations. The absence of data from the mothers during pregnancy is a clear weakness of our study. Several maternal conditions are known to have an impact on the babies’ development such as recent or ongoing GC treatment, psychiatric or metabolic diseases among several others. Therefore, maternal data need to be carefully collected in future prospective studies. In our experiments in mice we exposed mothers to corticosterone, while in the clinical setting synthetic GCs like dexamethasone or betamethasone are used instead. Therefore, the translation of our mouse data to the human situation requires to take this difference into account. Even though betamethasone and corticosterone have structural differences that can influence their bioavailability, both GCs have been described as full agonists of the GR^66^.

Despite the small number and a selection bias of preterm infants from three German Neonatal Network (GNN) sites, our findings suggest that optimizing the time of antenatal GC treatment may reduce the risk of negative long-term consequences on behavior. Giving antenatal GCs in the morning would be, under the right maternal circumstances, possible to implement in the clinic. In order to underscore these results mechanistically, it will be of interest to correlate injection time with behavioral scores in a cohort of infants whose mothers were injected with GCs but who were ultimately born full term.

## Methods

### Mouse models and housing conditions

All mice were housed under a 12-h light, 12-h dark (LD) cycle at 22 ± 2 °C and a relative humidity of 60 ± 5% with *ad-libitum* access to food (Supplementary Table 2) and water. For the gestational corticosterone (CORT) treatment, pregnant mice (C57BL/6J, *Bmal1*^+/−^ heterozygous knock-out mice (B6.129S4(Cg)-Arntltm1Weit/J) and Per1/2-doble mutants (B6.Cg-Per1tm1BrdTyrc-Brd/J & B6.Cg-Per2tm1Brd Tyrc-Brd/J) on a C57BL/6 J background) were subcutaneously injected with CORT either at the beginning (ZT0, Zeitgeber time 0 refers to the time of the day when the lights were switched on in the animal facility, in our case 6 a.m.) or at the end (ZT12, refers to the time of the day when the lights were switched off in the animal facility, in our case 6 p.m.) of the rest phase. Injections were performed daily from gestational day (GD) 11.5 until birth for experiments included in Figs. 1 and 2 and Supplementary Fig. 1, or until GD 15.5–16 for experiments included in Figs. 3, 4 and 5 and Supplementary Figs. 2–4. For mating, adult females were individually housed overnight in the presence of an experienced male. On the next day (GD 0.5), vaginal plugs were checked, and females were immediately separated and singly housed. Corticosterone (Supplementary Table 2) was dissolved in polyethylene glycol 400 (Supplementary Table 2) at a concentration of 20 mg/mL, sonicated on ice for 2 min and sterile filtrated. Injections were given at a concentration of 50 mg/kg body weight. The out-of-phase group received CORT every day at 6 a.m. (ZT0) and the in-phase group at 6 p.m. (ZT12). A naive group of mice was also assessed to confirm the influence of the prenatal manipulation itself.

### Behavioral tests

All offspring tested were males whose mothers were injected with corticosteone (as explained above), weaned at postnatal day 21 and left undisturbed until adulthood (2–4 months old) when the behavioral, systemic and molecular assessments of the stress axis were performed. The authors are aware of the importance of gender comparison; however, female offspring were not tested here. In addition to the strong interaction between behavior and cycling sex hormones levels, it is experimentally not possible to test all females at the same estrous cycle stage^67^. A maximum of 2 offspring per litter were included in each cohort for circadian locomotor activity assessment, anxiety-like and stress coping behavior assessment, fecal corticoid measurements in feces and HPA axis negative feedback assessment. At the end, all offspring from each group were assigned to each time point. A total of 21 and 17 litters were used from CORT injected mothers at ZT0 and ZT12, respectively.

Circadian locomotor activity was assessed by wheel-running activity in LD conditions (300 lx) for 2 weeks in individual cages, wheel revolutions were counted every minute (*n* = 5–11)^68^. Data collection and analysis were conducted using ClockLab software (Supplementary Table 2). The behavioral tests were performed only once per mouse in a soundproof room during the light phase (ZT4–8). The videos were analyzed using ANY-maze software (Supplementary Table 2). The EPM apparatus was illuminated with a white LED light source (200–230 lx). The animals were acclimatized to the room at least 30 min before starting the test. Mice were placed in the center of the maze facing a closed arm and allowed to move freely for 10 min. The number of entries and the time spent in the open arms was recorded (*n* = 10–11). To perform the FST, a 5-L glass beaker was filled with 3 L of warm tap water (25 ± 2 °C). The apparatus was illuminated with a white LED light source (200–230 lx). The groups were represented equally in every FST session. The test length was 6 min, but only the last 4 min of the test were analyzed for immobility behavior (*n* = 12).

### Hypothalamic-pituitary-adrenal (HPA) axis function in the offspring

Corticosterone was measured by radioimmunoassay (Supplementary Table 2) according to the manufacturer’s instructions either in serum or plasma depending on the experiment, in duplicates. Circadian profiles of plasma corticosterone were assessed in samples collected from mice kept in constant darkness (DD) at 6-h intervals on the second day after lights off (*n* = 7–11). Samples were collected in EDTA-coated tubes (Supplementary Table 2) and plasma isolated by centrifugation at 240 × *g*, 20 min, 4 °C. Fecal samples were collected from single-housed mice (*n* = 6) in wire bottom cages at 4-h intervals after an initial acclimatization for 72 h and stored at −80 °C until use. Fecal corticoid extraction was performed as previously described^69^. For corticosterone measurements in pregnant females, trunk blood was collected from mice kept in LD conditions at 6-h intervals or at ZT1 or ZT13 (*n* = 5–14) depending on the experiment in EDTA-coated tubes. Blood samples from the fetus were collected in capillary tubes and serum isolated by centrifugation at 240 × *g*, 20 min, 4 °C (*n* = 13–33). For corticosterone measurements after acute HPA axis activation and dexamethasone suppression test, blood samples were taken from the animal’s cheek in EDTA-coated tubes as described above. HPA axis activation by acute stress was assessed as the fold change between corticosterone levels before and 10 min after the FST session (*n* = 10–12). HPA axis negative feedback was assessed by a dexamethasone suppression test. Mice received a single injection of either saline or dexamethasone (i.p. 100 μg/kg b.w. in saline) (Supplementary Table 2) at ZT8 and blood samples were taken 6 h later (*n* = 5–9). Blood samples were collected and processed as detailed above and the HPA axis suppression effect of DEX was calculated as percentage compared to the saline injected mice. GR sensitivity was assessed by an ex vivo assay. Peripheral blood was incubated with LPS (Supplementary Table 2) to activate the secretion of interleukin 6 (IL-6). Blood samples were collected at ZT7 in EDTA-coated tubes. A 50-μL aliquot was incubated with 100 ng/mL of LPS or 1 μM DEX + LPS for 12 h at 37 °C. After incubation, samples (*n* = 4) were centrifuged (10,000 × *g* for 10 min) and IL-6 production quantified by ELISA (Supplementary Table 2). In presence of dexamethasone (DEX), the suppression of IL-6 production was used as an indicator of GR sensitivity. mRNA expression of glucocorticoid receptor (*Gr*) in the paraventricular nucleus (PVN) of the hypothalamus and the hippocampal formation (*n* = 5–12) was assessed by in situ hybridization (ISH) using ^35^S-rUTP (Supplementary Table 2) labeled probes on frozen coronal sections (12 µm). Tissue sections were fixed in 4% PFA in PBS and proteins were degraded by proteinase K treatment (20 µg/mL). Background signal was reduced by an acetylation step (50 mM acetic anhydride in 0.1 m TEA). Hybridization was performed overnight at 50 °C with cRNA anti-sense riboprobes transcribed from linearized plasmid DNA for Gr (forward primer: 5′ -AGGTCGACCAGCCGTCCAGA-3′; reverse primer: 5′-AAGCTTGCCTGGCAATAAAC-3′). Non-ligated RNA was removed by RNase digestion and several washing steps^70^. Quantification was performed by densitometric analysis of autoradiograph films using Quantity One 1-D analysis software (Supplementary Table 2) in three sections per animal, which were averaged.

### Gene expression analysis in fetal hypothalamus

All the experiments were run in male fetuses, only pregnancies with at least five fetuses were included. Fetal sex and genotype were determined by PCR as previously described^71,72^. Briefly, DNA was isolated 1 h at 55 °C with shaking from the fetal tail in 20 µL of 50 mM Tris, pH 8, 2 mM NaCl, 10 mM EDTA, 1% SDS containing 0.5 mg/mL of proteinase K. After 1:10 dilution with water, proteinase K was inhibited by incubation for 10 min at 95 °C. One microliter of DNA was used in 20 µL of PCR reaction containing 1x ammonium buffer, dNTPs, MgCl_2_ and Taq polymerase (Supplementary Table 2). Sex was determined by PCR (10 min at 94 °C, 33 cycles of (40 s at 94 °C, 60 s at 50 °C, 60 s at 72 °C) and 5 min 72 °C). The presence of IL-3 indicated females (544 bp) and the presence of IL-3 (544 bp) and SRY (402 bp) indicated males. The following primers were used at a concentration of 20 µM IL-3 (5′-GGGACTCCAAGCTTCAAT- 3′ and 5′-TGGAGGAGGAAGAAAAGCAA- 3′) and SRY (5′-TGGGACTGGTGACAATTGTC- 3′ and 5′-GAGTACAGGTGTGCAGCTCT- 3′). Fetal genotype was determined by PCR for *Per1* (3 min at 94 °C, 33 cycles of (30 s at 94 °C, 30 s at 65 °C, 1 min at 72 °C) and 10 min at 72 °C), for *Per2* (15 min at 94 °C, 37 cycles of (30 s at 94 °C, 30 s at 55 °C, 1 min at 72 °C) and 10 min at 72 °C) and *Bmal1* (3 min at 94 °C, 37 cycles of (30 s at 94 °C, 1 min at 59 °C, 1 min at 72 °C) and 5 min at 72 °C). The following primers were used *Per1*(5′ -AGAACTGAGGACCCAAGCTG- 3′; 5′-TTGCCCTACAGCCTCCTGAGT-3′ and 5′-GGGGAACTTCCTGACTAGGG-3′, giving bands of 600 bp (wild-type) or 400 bp (mutant). *Per2* (5′-GAACACATCCTCATTCAAAGG-3′; 5′- CGCATGCTCCAGACTGCCTTG-3′ and 5′ - GCTGGTCCAGCTTCATCAACC-3′, giving bands of 380 bp (wild-type) or 120 bp (mutant). *Bmal1* (5′-ACTGGAAGTAACTTTATCAAACTG-3′; 5′-CTGACCAACTTGCTAACAATTA-3′ and 5′-CTCCTAACTTGGTTTTTGTCTGT-3′, giving bands of 330 bp (wild-type) or 570 bp (mutant).

Fetal hypothalami were dissected and stored in RNAlater (Supplementary Table 2) or snap frozen at −80 °C. Total RNA was isolated using Allprep DNA/RNA/miRNA Universal kit (Supplementary Table 2) and reverse-transcribed to cDNA using random hexamer primers and the High Capacity cDNA Reverse Transcription Kit (Supplementary Table 2) according to the manufacturer’s instructions. qPCR reactions were carried out in duplicates for each sample using GoTaq qPCR Master Mix (Supplementary Table 2) on a CFX96 Real-Time PCR Detection System (Supplementary Table 2). The amplification efficiency of the target genes was comparable with the housekeeping gene *Eef1a1* (Elongation factor 1-alpha 1). Relative mRNA expression was calculated using the ΔΔCT method. Primer sequences are included in the supplementary material (Supplementary Table 2). For gene expression analysis sample sizes were between 4 and 12 samples/group. To assess gene expression in wild-types and *Per1/2* double heterozygous fetuses, a maximum of two male fetuses per litter in control conditions and after CORT injection were included. For gene expression in *Bmal1*^+*/+*^ and ^−/−^ fetuses we used only 1 male of each genotype per litter.

### Protein levels in fetal hypothalamus

Hypothalami were dissected from male fetuses, snap frozen in liquid nitrogen (N_2_) and conserved at −80 °C. Protein levels were assessed by Western blot (*n* = 4–6). Tissue was homogenized in 20 mM HEPES buffer, pH 7.5 containing 5 mM NaF, 10 μM Na_2_MoO_4_, 0.1 mM EDTA, and 2% Nonidet P-40. An aliquot of the total homogenate was used to assess protein content using BCA Protein Assay kit (Supplementary Table S2). An aliquot containing 30 μg of protein was incubated with 5X loading buffer at 95 °C for 8 min. Samples were resolved by 10% SDS-PAGE at 100 V and then transferred to PVDF membranes (Supplementary Table 2) for 1 h at 100 V and 4 °C. The membrane was blocked with 5% (w/v) blocker (Supplementary Table 2) in 25 mM Tris, pH 7.4, NaCl 138 mM and 0.1% (w/v) Tween-20 at 25 °C for 1 h and then incubated overnight at 4 °C with either anti-GR (1:1,000), anti-REVERBα (1:1,000) or anti-αTubulin (1:1,000) antibodies (Supplementary Table 2). When REVERBα and GR were developed from the same membrane a stripping step of 1 h at 37 °C was performed (Supplementary Table 2), followed by 1 h incubation in blocking solution. Antibody reactions were visualized by ECL chemiluminescence (Supplementary Table 2) after incubation for 1 h at room temperature with the respective HRP-coupled secondary antibody (Goat anti-Rabbit HRP (1:20,000) and Horse anti-Mouse HRP (1:3,000), Supplementary Table 2). Scans were obtained using the ChemiDoc Touch Imaging System using the optimal auto-exposure option to avoid saturation of the signal (Supplementary Table 2) and quantified with Image Lab software (Supplementary Table 2). To assess protein levels, a maximum of two (wild-types) or one (*Bmal1*^+*/+*^ and ^−/−^) male fetuses per litter were included in both naive and after CORT conditions.

### GR binding in fetal hypothalamus

GR binding was assessed using the TRANS AM GR activity kit (Supplementary Table 2). Hypothalami from male fetuses were dissected and immediately frozen in liquid N_2_ (*n* = 4). The assay was performed in duplicates following the manufacturer’s instructions. Only one male fetus per litter was included in naive and after CORT conditions.

Overall, for the experiments in offspring a total of 21 and 17 litters were included from CORT injected mothers at ZT0 and ZT12, respectively. For the experiments with fetuses a total of 11 (naive ZT1), 5 (naive ZT7), 13 (naive ZT13), 6 (naive ZT19), 14 (CORT out-of-phase ZT1), 7 (CORT out-of-phase ZT13), 9 (CORT in-phase ZT1), and 8 (CORT in-phase ZT13) litters from wild-type mothers; 4 (naive ZT1, CORT out-of-phase ZT1 and CORT in-phase ZT13) and 6 (naive ZT13) litters from *Per1/2* double mutant mothers and 8 (naive ZT1), 7 (naive ZT13), 10 (CORT out-of-phase ZT1) and 12 (CORT in-phase ZT13) litters from *Bmal1*^+/−^ mothers were included in the whole study.

### Retrospective observational analysis from the German Neonatal Network (GNN) cohort

The GNN is a population-based observational multicenter cohort study enrolling very low birth weight (VLBW) infants at neonatal intensive care units (NICUs) throughout Germany. Study data were gathered from infants born between January 1st, 2009 and December 31st, 2013. For the purpose of this study, we retrospectively collected data on the timing of antenatal synthetic GC (betamethasone, 8 or 12 mg) injections—at least two injections 24 h appart—given to the mother between weeks 24 and 34 of gestation in Lübeck, Cologne, or Essen. Exclusion criteria were dexamethasone treatment (5 cases; since the doses are given every 12 h), single betamethasone treatments (1 case), birth before gestational week 24 (18 cases) and incomplete or unreliable 5-year questionnaires (10 cases). Of 141 cases 107 preterm infants met all criteria.

Depending on the difference (in hours) between the time of maternal physiological cortisol peak (estimated at 8:00,^73^) and the time of antenatal betamethasone injection, preterm infants were divided into two groups; the in-phase group (33), injected between 4:00 and 12:00 and the out-of-phase group (20), injected between 18:00 and 0:00. Fifty four children out of *n* = 107 belong to the middle group whose mothers were injected from 12:00 to 18:00 and from 0:00 to 4:00. During the 5-year follow-up infants were examined by the same GNN study team (a physician trained in neonatology and two study nurses) at all sites. Parents provided a written informed consent to answer questions about previous medical history and current medical needs as well as to complete a questionnaire concerning detailed information on the children’s social background, illnesses, general development and behavior. Participants (infants and parents) and hospital care givers were blinded to the group assignment. A compound behavioral score (0 to 10) was used to quantify stress compensation capacity for each child. Questionnaires were based on the German Health Interview and Examination Survey for Children and Adolescents^50,51^. The single-item answers are plotted for both groups (Supplementary Fig. 5a–e and Supplementary Table 1). The introductory question was: Which of the following descriptions holds true for your child? (Please consider your child’s behavior during the last 6 months). To calculate the behavioral score for both groups, we assigned values to the answers Yes = 2, somewhat or a bit = 1 and No = 0 and summed them up. The descriptors used were: (a) My child is often impatient, hyperactive, cannot sit still. (b) My child has frequent fits of rage and is quick-tempered (choleric). (c) My child is constantly nervous and fidgety. (d) My child is afraid of new situations and easily loses self-confidence. (e) My child has many anxieties and gets scared easily. A scanned version of the questionnaire is available as Supplementary Material 4.

### Statistical analysis

All statistical analyses were carried out using GraphPad Prism 8.0, CircWave v1.4 and Jamovi 1.1.9.0 (Fig. 6 and Supplementary Fig. 5) and data were plotted as means ± SEM. Mann–Whitney (two-sided), unpaired *T*-tests (two-sided), 1-way and 2-way ANOVAs with Sidak’s multiple-comparison tests were employed as appropriate following confirmation of test assumptions. Normality was tested by D’Agostino-Pearson, Shapiro–Wilk, and Kolmogorov–Smirnov tests. Linear correlation between behavioral outcomes and *Gr* expression in the PVN was assessed by Pearson’s correlation test (Fig. 2f, g). Additionally, a *χ*^2^ test for association to test for potential confounding factors of in-phase vs. out-of-phase preterm infant group membership (Table 1) and a general linear model adjusting for mode of delivery as a confounder when regressing behavioral summed scores on group (Fig. 6, Supplementary Fig. 5 and Supplementary Table 1) were employed. Circadian rhythmicity was assessed using CircWave v1.4^73^. Sample size was determined using G-power analysis software 3.1.9.1, University of Düsseldorf, Germany. *P*-values below 0.05 were considered statistically significant.

### Study approval

All experiments in mice were ethically approved by the Committee on Animal Health and Care of the Government of Schleswig-Holstein (V 242–7224.122-4(45-4/15) and V 242–7604/2017 (37-3/17) and were performed according to international guidelines on the ethical use of animals. The GNN is supported by the German Federal Ministry for Education & Research (BMBF; code: 01ER1501; https://www.gesundheitsforschung-bmbf.de/de/deutsches-fruhgeborenen-netzwerk-german-neonatal-network-gnn-3798.php) and approved by the University of Lübeck ethics committee (08–022) and the institutional review boards of all participating sites. The study protocol can be accessed at https://www.vlbw.de/ (in German). English translations of the study protocol and the informed consent form are provided in the Supplementary Material 2 and 3.

### Reporting summary

Further information on research design is available in the Nature Research Reporting Summary linked to this article.

## Supplementary information

Supplementary Information

Peer Review File

Reporting Summary

## Data Availability

All raw data of human and mouse experiments included in the main figures and the supplementary are provided as a Source Data file.

## Source data

Source Data
