# Supplementary material for: The circadian phase of antenatal glucocorticoid treatment affects the risk of behavioral disorders
Source: Nat Commun. 2020 Jul 17;11:3593. doi: 10.1038/s41467-020-17429-5 (PMC7367845; doi:10.1038/s41467-020-17429-5)
Supplement: Supplementary file 1 — Supplementary Information [file 41467_2020_17429_MOESM1_ESM.pdf]

Supplementary information

THE CIRCADIAN PHASE OF ANTENATAL GLUCOCORTICOID TREATMENT AFFECTS THE RISK OF BEHAVIORAL DISORDERS

Astiz et al.

Supplementary figures

Supplementary Fig 1:

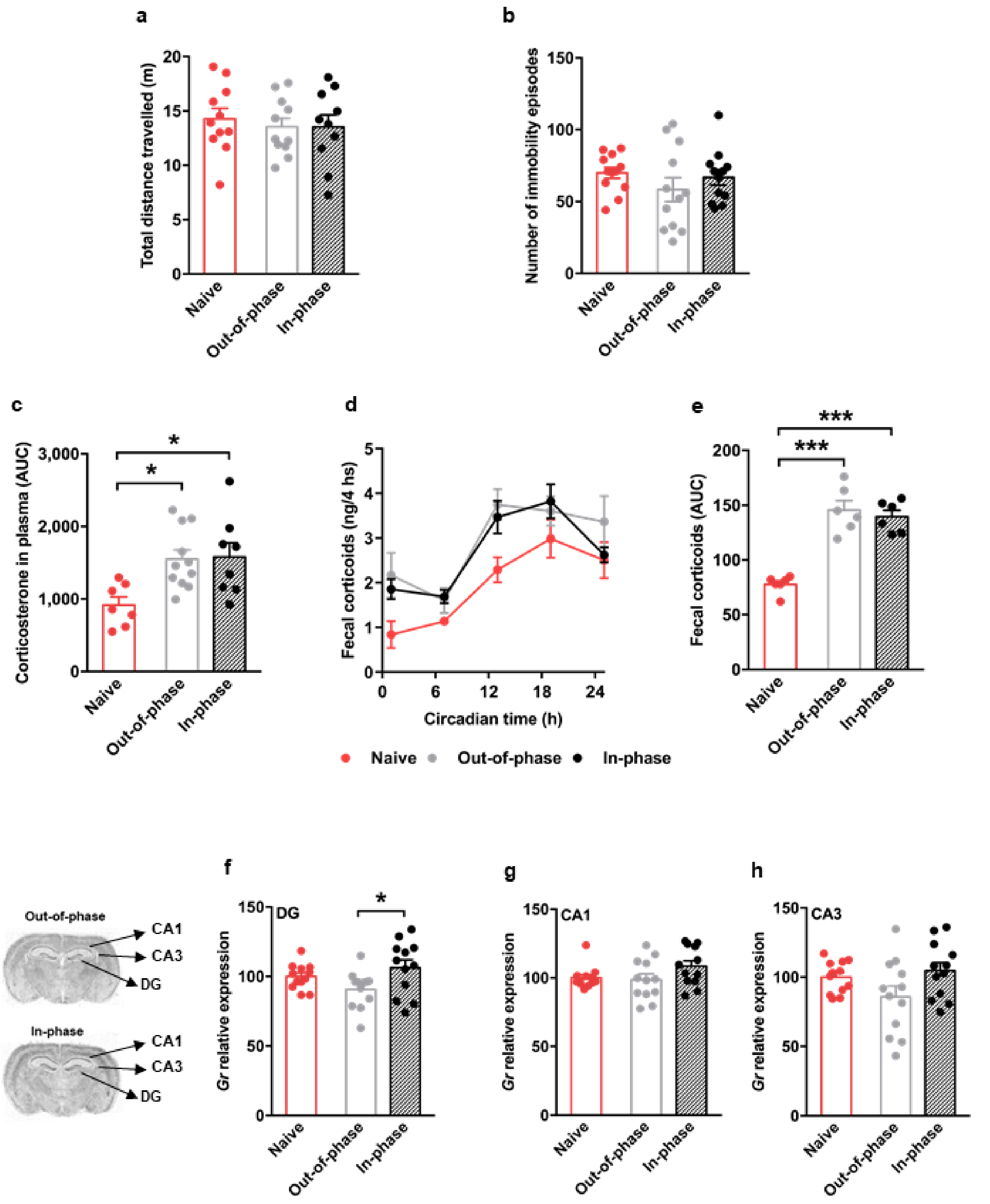

(a) Total distance travelled in the elevated plus maze (EPM) in m (naive n=11, out-of-phase n=11, in-phase n=10). (b) Number of immobility episodes in the forced swim test (FST) (n=12 for all groups).

All behavioral tests were performed once. (c) Area under the curve (AUC) of circadian profiles of plasma corticosterone (CORT) (Fig. 1f) (naive n=7, out-of-phase n=11, in-phase n=8 mice). (d) Circadian profiles of corticoids in feces, (naive n=6 per time point/group). (e) Area under the curve (AUC) of circadian profiles of corticoids in feces (Supplementary Fig. 1d), (n=6 group). Corticosterone measurements were done in duplicates. (f-h) Glucocorticoid receptor (*Gr*) relative expression at the hippocampal formation in dentate gyrus (DG), *cornu armonis 1* and *3* (CA1 and CA3), respectively, determined by *in situ* hybridization (3 sections were quantified and averaged/mice, n=12 for all the groups). On the left, representative images show the quantified region. Data are expressed as means ± SEM and analyzed by one-way ANOVA (in c, e and f) followed by Sidak's adjusted test for multiple comparisons ((c) $F(2,23)=5.57$, $p=0.011$; (e) $F(2,15)=35.59$, $p<0.0001$; (f) $F(2,33)=3.34$, $p=0.047$. * $p<0.05$, *** $p<0.001$. Source data are provided as a Source Data file.

Supplementary Fig 2:

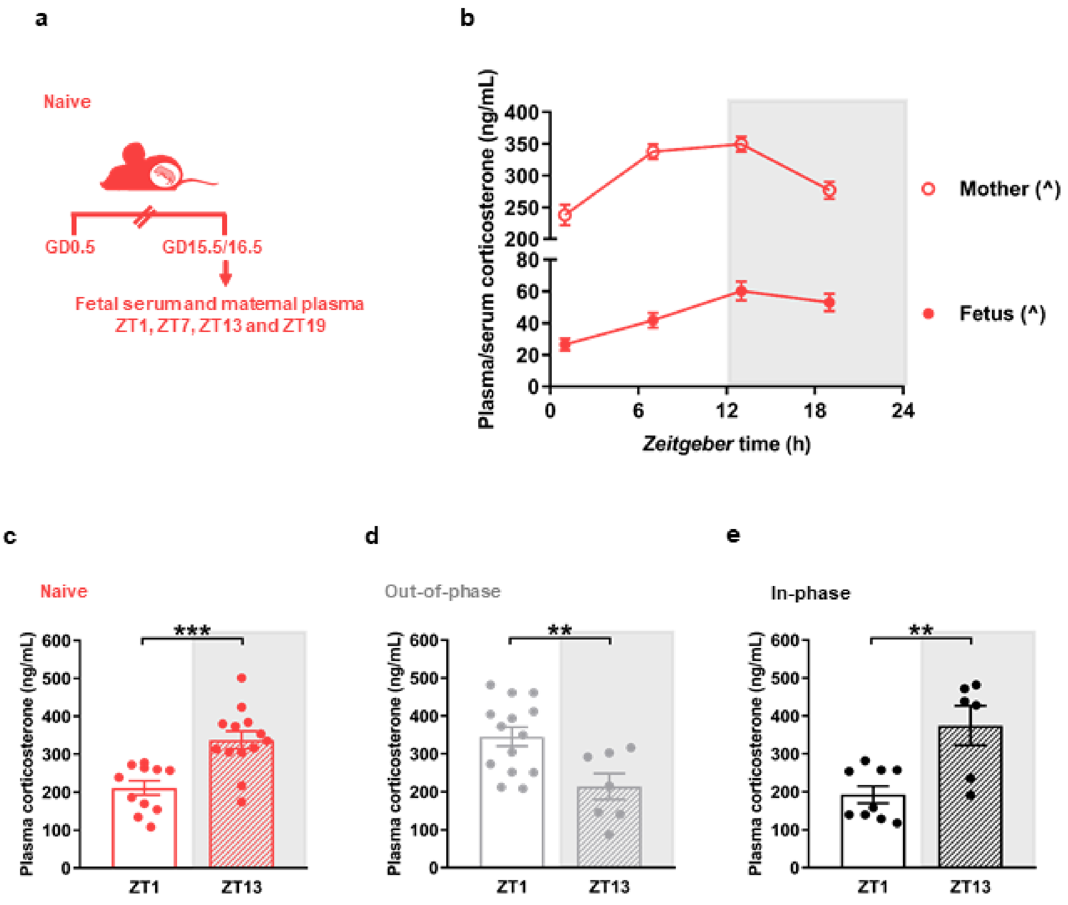

(a) Scheme of the sample collection procedure. Naive pregnant mice were euthanized every 6 h (*Zeitgeber* time (ZT) 1, 7, 13 and 19) during gestational day (GD) 15.5 to 16.5 to collect maternal and fetal blood. (b) Circadian profiles of maternal plasma and fetal serum corticosterone levels, n=16-23 fetus and n=5-13 mothers. Significant circadian rhythmicity was determined by CircWave v1.4 which uses a Monte Carlo surrogate approach to fit a standard cosine function to an averaged de-trended data assuming a period of 24 hr, a $p<0.05$ was considered significant (mother: r^2 =0.70, $p<0.001$; fetus: r^2 =0.21, ^ $p<0.001$). (c) Maternal corticosterone levels in plasma at ZT1 and ZT13 in naive pregnant mice (n=11 ZT1 and n=13 ZT13), (d) pregnant mice injected at ZT0 (n=14 ZT1 and n=7 ZT13) and (e) pregnant mice injected at ZT12 (n=9 ZT1 and n=6 ZT13). Corticosterone measurements were done in duplicates. Data are expressed as means ± SEM and analyzed by two-sided unpaired t-tests ((c) t=4.11, df=22, p=0.0005; (d) t=3.02, df=19, p=0.007; (e) t=3.63, df=13, p=0.003). **p<0.01, ***p<0.001. Source data are provided as a Source Data file.

Supplementary Fig 3:

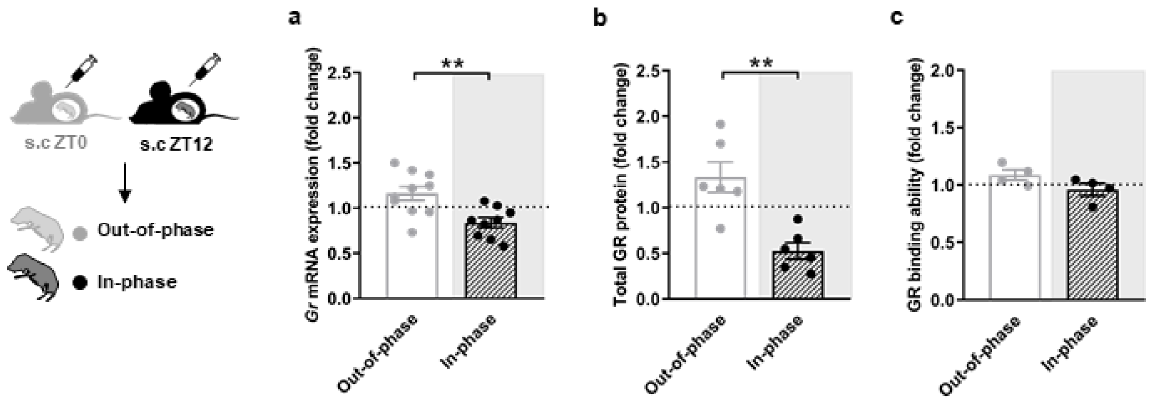

(a) Glucocorticoid receptor (*Gr*) mRNA expression in the hypothalamus of fetus from pregnant mice injected with corticosterone (CORT) at *Zeitgeber* time (ZT) 0 (out-of-phase) or at ZT12 (in-phase). The dotted line represents the levels of *Gr* mRNA in fetus from naive mothers (naive ZT1 n=7, out-of-phase ZT1 n=10, naive ZT13 n=8 and in-phase ZT13 n=9). (b) GR protein in whole fetal hypothalami assessed by Western blot. The dotted line represents the levels of GR protein in fetus from naive mothers (naive ZT1, out-of-phase ZT1, naive ZT13 and in-phase ZT13 all n=6). (c) GR binding to glucocorticoid receptor binding elements (*GRE*s) assessed in fetal hypothalami. The dotted line represents the GR binding ability in fetus from naive mothers (naive ZT1, out-of-phase ZT1, naive ZT13 and in-phase ZT13 all n=4). qPCRs for gene expression, western blot and GR binding assays were run in duplicates. All data are expressed as means ± SEM. Data were analyzed by two-sided unpaired t-tests ((a) t=3.386, df=17, p=0.0035; (b) t=4.257, df=10, p=0.0017). **p<0.01. Source data are provided as a Source Data file.

Supplementary Fig 4:

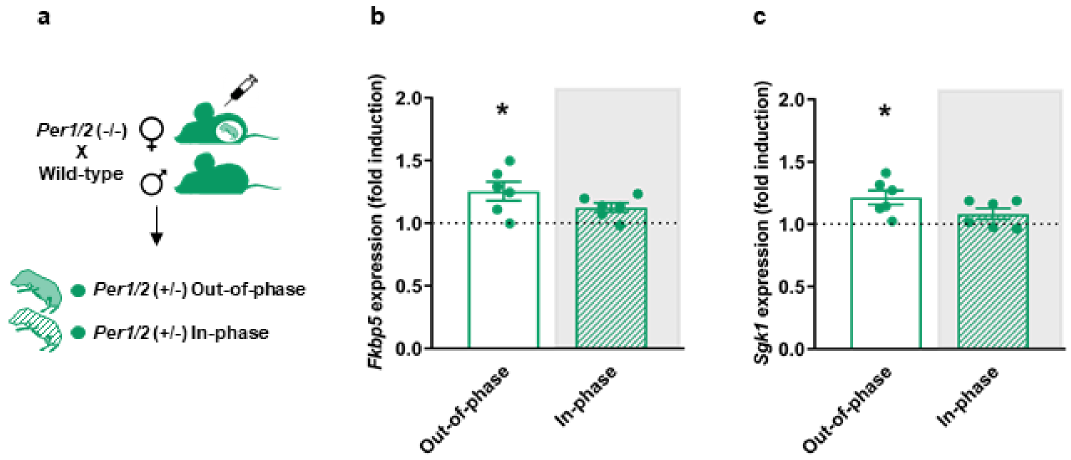

(a) Scheme of the prenatal timed corticosterone (CORT) intervention in *Period* (*Per*)*1/2* double knock-out pregnant mice mated to wild-type males. (b and c) mRNA relative expression of the glucocorticoid receptor (GR) targets FK506 binding protein 5 (*Fkbp5*) and Serum glucocorticoid-regulated kinase 1 (*Sgk1*) in the hypothalamus of double *Per1/2* heterozygous fetus. Gene expression is shown as fold induction after CORT treatment (at *Zeitgeber* time (ZT) 0 or ZT12) relative to naive fetus of the same genotype (dotted line) (n=6 for all groups naive ZT1, out-of-phase ZT1, naive ZT13 and in-phase ZT13). qPCRs for gene expression were run in duplicates. Data are expressed as means ± SEM. Data were analyzed by two-sided unpaired t-tests (b) *Fkbp5*: Naive ZT1 vs out-of-phase ZT1 t=2.68, df=10, p=0.023; (c) *Sgk1*: Naive ZT1 vs out-of-phase ZT1 t=2.58, df=9, p=0.031). *p<0.05. Source data are provided as a Source Data file.

Supplementary Fig 5:

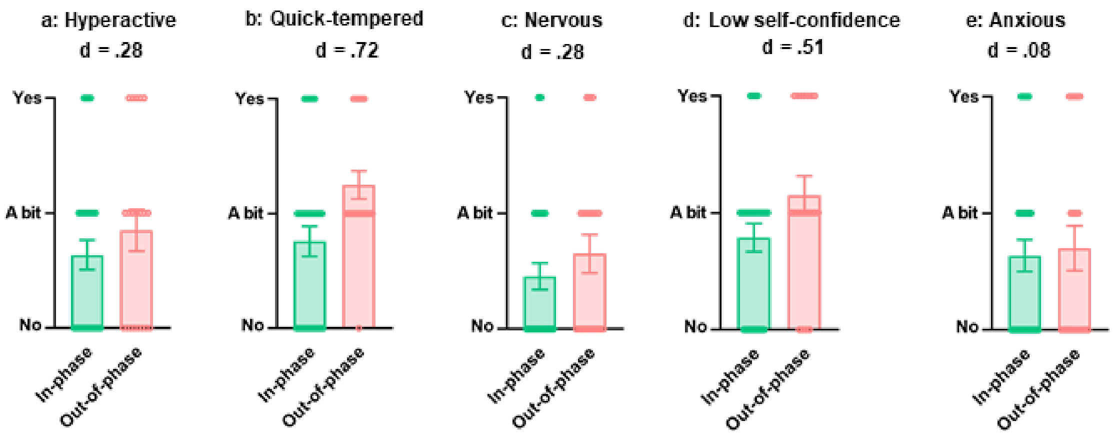

(a-e) Sub-scores as rated by the preterm infants' parents (from the three centers) to the questionnaire based on the German Health Interview and Examination Survey for Children and Adolescents are plot for both groups (in-phase n=33 and out-of-phase n=20) [50]. To calculate the behavioral score shown in Fig. 5b, we assigned values to the answers Yes=2, A bit=1 and No=0 to the features detailed below and summed all answers. The introductory question was: Which of the following descriptions hold true for your child? (Please consider your child' behavior within the last 6 months). (a) My child is often impatient, hyperactive, cannot sit still. (b) My child has frequent fits of rage and is quick-tempered (choleric). (c) My child is constantly nervous and fidgety. (d) My child is afraid of new situations and easily loses self-confidence. (e) My child has many anxieties and gets scared easily. The score obtained for each feature in both groups is expressed as mean ± SEM, and Cohen's d (standardized mean difference) is provided as an indication of effect size (a-e). Source data are provided as a Source Data file.

Supplementary tables

Supplementary Table 1: Statistic analysis of sub-scores shown in Supplementary Fig. 5.

							95% Confidence Interval		
		statistic	df	p	Mean difference	SE difference	Lower	Upper	Cohen's d
Hyperactive	Student's t	-0.980	51.0	0.33175	-0.2136	0.218	-0.651	0.2240	-0.2777
	Mann-Whitney U	282		0.34431	-2.04e−5		-1.000	3.32e-5	-0.2777
Quick-tempered	Student's t	-2.543	51.0	0.01406	-0.4924	0.194	-0.881	-0.1037	-0.7207
	Mann-Whitney U	207		0.01425	-1.000		-1.000	-7.64e−5	-0.7207
Nervous	Student's t	-0.990	51.0	0.32663	-0.1955	0.197	-0.592	0.2007	-0.2807
	Mann-Whitney U	282		0.32095	-6.72e−5		-1.000	4.82e-5	-0.2807
Low self-confidence	Student's t	-1.787	51.0	0.07980	-0.3621	0.203	-0.769	0.0446	-0.5065
	Mann-Whitney U	243		0.08449	-3.14e−5		-1.000	9.55e-5	-0.5065
Anxious	Student's t	-0.276	51.0	0.78389	-0.0636	0.231	-0.527	0.3998	-0.0781
	Mann-Whitney U	321		0.86265	-2.33e−5		-6.00e−6	1.28e-5	-0.0781

Supplementary Table 2: Antibodies, reagents, kits, real-time qPCR primer sequences and software used.

	Description	Catalog #	Company	Country
**Antibodies**				
Rabbit anti-GR	Dilution 1:1000	Ab183127	Abcam	Cambridge, UK
Rabbit anti-RevErbα	Dilution 1:1000	PA5-29865	Thermo Fisher Sci	Massachusetts, USA
Mouse anti-αTubulin	Dilution 1:1000	T5168	Sigma-Aldrich	Missouri, USA
Goat anti-Rabbit HRP	Dilution 1:20000	A9169	Sigma-Aldrich	Missouri, USA
Horse anti-Mouse HRP	Dilution 1:3000	7076S	Cell Signaling	Massachusetts, USA
**Reagents**				
Corticosterone	≥98.5% (HPLC)	27840	Sigma-Aldrich	Missouri, USA
Poly(ethylene glycol)	BioUltra, 400	91893	Sigma-Aldrich	Missouri, USA
Chow	3.457 Kcal/g	Mouse breeding	Ssniff	Soest, Germany
EDTA-coated tubes	Microvette CB300 EDTA	NC9141704	Fisher Sci.	New Hampshire, USA
Dexamethasone	Water soluble, suitable for cell culture	D2915	Sigma-Aldrich	Missouri, USA
Lipopolysaccharide	from Escherichia coli O111:B4	L2630	Sigma-Aldrich	Missouri, USA
Taq DNA polymerase	50 UI/µL	A111103	Ampliqon	Odense, Denmark
dNTP mix	10 µM	R0192	Thermo Fisher Sci.	Massachusetts, USA
UTPαS	[35S]- 1250 Ci/mmol, 12.5 mCi/ml, 250 µCi	NEG039H250UC	PerkinElmer	Massachusetts, USA
ECL films	Amersham Hyperfilm ECL	28906835	GE Healthcare, Life Sci.	Illinois, USA
RNAlater	RNA Stabilization Solution	AM7021	Thermo Fisher Sci.	Massachusetts, USA
PVDF membrane	Membranes for western blotting	3010040001	Sigma-Aldrich	Missouri, USA
Blotting-Grade Blocker	Nonfat dry milk for western blotting	1706404	Bio-Rad	California, USA

Restore Westen Blot buffer	Stripping buffer	21059	Thermo Fisher Sci.	Massachusetts, USA
Clarity Max Western ECL Substrate	ECL sustrate for HRP-conjugated antibody	1705062S	Bio-Rad	California, USA
**Kits**				
Corticosterone RIA	Radioimmune assay for mouse corticosterone	07-120103	MP Biomedicals	Eschwege, Germany
IL-6 ELISA	Enzyme immunoabsorbent assay for mouse IL-6	RAB0314	Sigma-Aldrich	Missouri, USA
Allprep DNA/RNA/miRNA Universal kit	RNA and DNA isolation kit	80224	Qiagen	Hilden, Germany
High Capacity cDNA Reverse Transcription Kit	cDNA synthesis	4368813	Thermo Fisher Sci.	Massachusetts, USA
GoTaq qPCR Master Mix	qPCR kit	A6002	Promega	Wisconsin, USA
BCA Protein Assay Kit	Protein concentration kit	23225	Thermo Fisher Sci.	Massachusetts, USA
TRANS AM GR activity kit	GR activity	45496	ActiMotif	California, USA

**Real-time quantitative PCR primer’s sequences**		
**Gene**	**Forward 5´- 3´**	**Reverse 5´- 3´**
Gr	CAAGGGTCTGGAGAGGACAA	TACAGCTTCCACACGTCAGC
RevErbα	AGCTCAACTCCCTGGCACTTAC	CTTCTCGGAATGCATGTTGTTC
Fkbp5	GGGTGTACGCCAACATGTTC	GAGGAGGGCCGAGTTCATT
Sgk1	CTTCTGGACTAATGATGATGCAGC	TGCCCTTTCCGATCACTTTC
Eef1α1	TGCCCCAGGACACAGAGACTTCA	AATTCACCAACACCAGCAGCAA

**Softwares**			
Image Lab™ Software	Western blot quantification	Bio-Rad	California, USA
Quantity One 1-D analysis software	ISH quantification	Bio-Rad	California, USA
CFX96 Real-Time PCR Detection System	qPCR detection and analysis	Bio-Rad	California, USA
GraphPad Prism 8	Statistic analysis	GraphPad software	California, USA
Microsoft Office 2016	Data analysis	Microsoft	Washington, USA
Any-maze	Behavioral tests analysis	Any-Maze	Dublin, Ireland
ClockLab software	Circadian data analysis	Actimetrics	Evanston, IL, USA

Supplementary Material

Supplementary Material 1: Study protocol of the human study (in German).

Supplementary Material 2: Study protocol of the human study (English translation).

Supplementary Material 3: Declaration of parents' consent to answer 5-year old follow-up questionnaires (in English).

Supplementary Material 4: Follow-up questionnaire content: scanned booklet with all questions of the 5-year old follow-up (in German).

Universität Lübeck

UNIVERSITÄTSKLINIKUM Schleswig-Holstein
Klinik für Kinder- und Jugendmedizin

Studienprotokoll 1/2008

Version 2 vom 14.6.2008

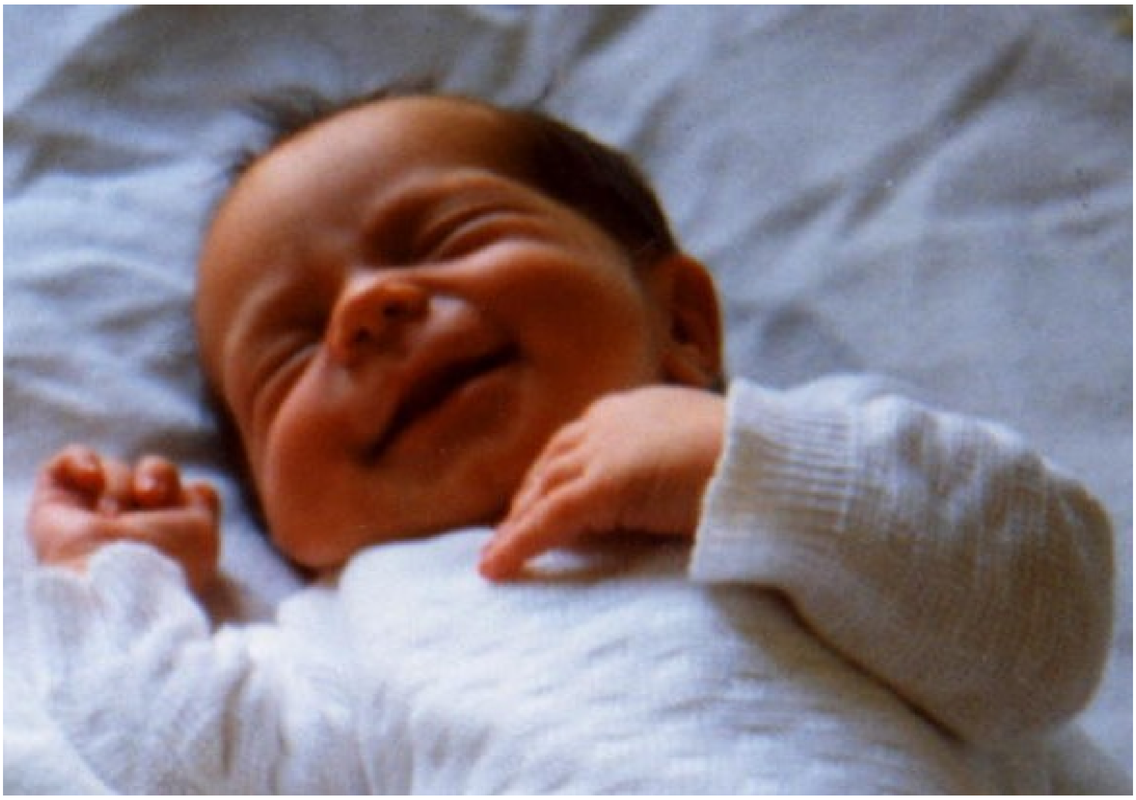

Deutsches Frühgeborenen-Netzwerk

German Neonatal Network (GNN)

<u>Verantwortlicher Studienleiter:</u>
PD Dr. med. Wolfgang Göpel
Universität Lübeck
Universitätsklinikum Schleswig-Holstein
Klinik für Kinder- und Jugendmedizin
Ratzeburger Allee 160, 23538 Lübeck
Tel.: 0451-500-2555
Fax: 0451-500-2590
Email: goepel@paedia.ukl.mu-luebeck.de

Inhaltsverzeichnis

		Seite
**1.**	**Zusammenfassung**	3
**2.**	**Projektmanagement und an der Studie beteiligte Institutionen**	5
**3.**	**Hypothesen und Ziele**	8
**4.**	**Stand der Forschung, eigene Vorarbeiten, Perspektive der Studie**	9
**5.**	**Studiendesign**	13
**6.**	**Ethik**	24
**7.**	**Kosten**	24
**8.**	**Ausgewählte Publikationen**	25
**9.**	**Anhänge: Dokumentationsbögen, Aufklärungs- und Einverständnisbögen**	26

1. Zusammenfassung

Der wesentliche Anteil von Mortalität und Morbidität bei Neugeborenen in Deutschland ist durch Erkrankungen einer kleinen Gruppe von Kindern bedingt: Frühgeborene mit einem Geburtsgewicht unter 1500 Gramm.

Wichtigstes Ziel für alle Personen, die Frühgeborene betreuen, ist die ungestörte körperliche und geistige Entwicklung dieser Kinder, die in der Regel erst in einem Alter von 5-6 Jahren beurteilt werden kann. Leider gibt es in Deutschland, im Gegensatz zu vielen anderen Ländern, kein neonatologisches Netzwerk, dass sich die Erforschung von Determinanten der langfristigen Entwicklung von Frühgeborenen zum Ziel gesetzt hat.

Ziel des vorliegenden Studienprotokolls ist die Etablierung eines deutschen Frühgeborenen-Netzwerks, das

- o die Effekte genetischer und klinischer Risikofaktoren
- o die Einflüsse zentrumsspezifischer Behandlungsstrategien und
- o die Auswirkungen randomisierter Studien

auf die langfristige Entwicklung von Frühgeborenen mit einem Geburtsgewicht unter 1500 Gramm untersucht.

Eine Reihe von Faktoren begünstigen dieses Vorhaben:

- o Im Jahr 2005 verpflichtete der Gemeinsame Bundesausschuss alle neonatologischen Abteilungen zur entwicklungsneurologischen Nachuntersuchung aller Frühgeborenen mit einem Geburtsgewicht unter 1500 Gramm im Alter von 2 Jahren.
- o Der im Jahr 2007 publizierte Kinder- und Jugendgesundheitssurvey (KiGGS), in dem eine repräsentative Stichprobe von 17641 deutschen Kindern im Alter von 0-17 Jahren untersucht wurde, stellt einen einzigartigen Pool von Vergleichsdaten für die Entwicklung Frühgeborener dar und bietet darüber hinaus eine Vielzahl von validierten Messinstrumenten zur Gesundheit von Kindern.
- o Ebenfalls im Jahr 2007 konnte in genomweiten Studien an Erwachsenen erstmals gezeigt werden, dass die zuverlässige Identifizierung von genetischen Einflussfaktoren für häufige Erkrankungen möglich ist.

Unsere Arbeitsgruppe arbeitet seit mehr als 10 Jahren an der Erforschung genetischer Risikofaktoren für Erkrankungen von Frühgeborenen. Unsere DNA-Bank umfasst mehr als 3000 DNA-Proben von Frühgeborenen mit einem Geburtsgewicht unter 1500 Gramm und ist damit eine der größten Frühgeborenen-DNA-Banken weltweit.
Wir erwarten, dass Umweltfaktoren (z.B. unterschiedliche Behandlungsstrategien verschiedener Neonatologien, soziale Herkunft etc.) stärkere Effekte auf die langfristige Entwicklung von Frühgeborenen haben, als die Auswirkungen genetischer Determinanten. Im Rahmen des vorliegenden Protokolls sollen deshalb nicht nur genetische Einflussfaktoren sondern auch die unterschiedlichen zentrumsspezifischen Behandlungsstrategien, der Einfluss des sozialen Umfelds und schließlich die Auswirkungen von neuen Therapieformen – wie z.B. die Verabreichung von Surfactant ohne Intubation – auf die langfristige Entwicklung von Frühgeborenen untersucht werden.
Ziel der Studie ist die bei der Geburt beginnende prospektive Dokumentation der klinischen Daten von 20.000 Frühgeborenen mit einem Geburtsgewicht unter 1500 Gramm, von denen DNA-Proben eingefroren werden und deren weitere körperliche und geistige Entwicklung im Alter von 2 und 6 Jahren untersucht wird.
Wir hoffen, durch diese dem Umfang nach in der deutschen Neonatologie bislang einzigartige Studie die langfristige Entwicklung der uns anvertrauten kleinen Patienten nachhaltig zu verbessern.

2. Projektmanagement

**Projektmanagement**		
**Name**	**Klinik/Institut**	**Funktion**
Prof. Dr. Wolfgang Göpel	Klinik für Kinder- und Jugendmedizin, Universität Lübeck	Principal Investigator, Verantwortlicher Studienleiter für Design, Management, Koordination und Analyse der Studie
Prof. Dr. Egbert Herting	Klinik für Kinder- und Jugendmedizin, Universität Lübeck	Studienleiter, Koordination, Vorstandsmitglied der Gesellschaft für Neonatologie und Pädiatrische Intensivmedizin (GNPI)
Prof. Dr. Andreas Ziegler	Universität Lübeck, Institut für Medizinische Biometrie und Statistik	Verantwortlich für Qualitätssicherung, Datenmanagement und Biometrie
Prof. Dr. Peter Nürnberg	Cologne Center for Genomics	Genome wide SNP-genotyping
Prof. Dr. Heribert Schunkert, Priv. Doz. Dr. Jeanette Erdmann	Univeristät Lübeck, Medizinische Klinik 2	Genotypisierung (Bestätigung assoziierter SNPs)
**Projektbeirat**		
Priv. Doz. Dr. Heike Rabe	Brighton and Sussex University Hospitals NHS Trust, UK	Vorstandsmitglied der European Society of Paediatric Research (ESPR)
Prof. Dr. Jens Möller	Kinderklinik Saarbrücken	Vorstandsmitglied der European Society of Neonatology and Paediatric Intensive Care (ESPNIC)
Prof. Dr. Wolke	University of Warwick, UK	Besondere wissenschaftliche Expertise bezüglich Nachbeobachtungsstudien von Frühgeborenen (e.g. EPICURE)
Dr. Bärbel-Maria Kurth	Robert-Koch Institut, Berlin	Besondere wissenschaftliche Expertise bezüglich Surveyuntersuchungen bei Kindern (KiGGS)
Prof. Dr. Ute Thyen	Universitätskinderklinik Lübeck	Vorstandsmitglied der Deutsche Gesellschaft für Sozialpädiatrie und Jugendmedizin
Prof. Dr. Ulrich Gembruch	Universität Bonn, Klinik für Frauenheilkunde und Geburtshilfe	Vorstandsmitglied der Deutschen Gesellschaft für Perinatale Medizin
Prof. Dr. Dr. Heiner Raspe	Universität Lübeck, Institut für Sozialmedizin	Besondere wissenschaftliche Expertise bezüglich Versorgungsforschung
Silke Mader	Bundesverband “Das frühgeborene Kind”	Vorstandsvorsitzende des Bundesverbandes “Das frühgeborene Kind”

Vorläufige Liste der teilnehmende Kliniken (weitere Kliniken haben ihre Teilnahme bereits zugesagt)

**Klinik**	**Leiter der Studie in der teilnehmenden Klinik**	**Ethik-Kommission**
Klinik für Kinder und Jugendmedizin<br>UKSH Campus Lübeck<br>Ratzeburger Allee 160<br>23538 Lübeck	Prof. Dr. W. Göpel<br>Prof. Dr. Egbert Herting	Ethik-Kommission der Medizinischen Fakultät<br>Universität Lübeck<br>Ratzeburger Allee 160<br>23538 Lübeck
Altonaer Kinderkrankenhaus<br>Bleickenallee 38<br>22763 Hamburg	Dr. Axel v.d.Wense	zur Weiterleitung an die Ethikkommission<br>Prof. Dr. Frank Riedel<br>Ärztlicher Direktor des Altonaer Kinderkrankenhauses<br>Bleickenallee 38<br>22763 Hamburg
Klinik für Kinder und Jugendmedizin<br>Winterberg 1<br>66119 Saarbrücken	Prof. Dr. Jens Möller	zur Weiterleitung an die Ethikkommission<br>Prof. Dr. Jens Möller<br>Chefarzt der Klinik für Kinder- und Jugendmedizin<br>Winterberg 1<br>66119 Saarbrücken
Klinik für Allgemeine Pädiatrie<br>Moorenstr. 5<br>40225 Düsseldorf	PD Dr. Thomas Höhn	Prof. Dr. Hans Gerd Lenard<br>Vorsitzender der Ethikkommission der Heinrich Heine Universität<br>Moorenstr. 5<br>40225 Düsseldorf
Klinik für Kinder- und Jugendmedizin<br>Am Hasenkopf 1<br>63739 Aschaffenburg	Dr. Christian Wieg	Ethik-Kommission der Medizinischen Fakultät<br>Josef Schneider Str. 2, D7<br>97080 Würzburg
Klinik und Poliklinik für Allgemeine Kinderheilkunde<br>Joseph-Stelzmann Str. 9<br>50924 Köln	Dr. Angela Kribs<br>Prof. Dr. Bernhard Roth	Ethik-Kommission der Medizinischen Fakultät<br>Joseph-Stelzmann-Str. 20<br>Forum/Gebäude 42<br>50931 Köln
Universitätskinderklinik Bochum<br>St. Josefs-Hospital<br>Alexandrinenstraße 5<br>44791 Bochum	Dr. Norbert Teig	Ethikkommission der Medizinischen Fakultät Ruhr-Universität<br>Bürkle-de-la-Camp Platz 1<br>44789 Bochum
Universitätskinderklinik Schleswig-Holstein<br>Klinik für Allgemeine Pädiatrie<br>Schwanenweg 20<br>24105 Kiel	PD Dr. Werner Nikischin	Ethikkommission der Medizinischen Fakultät Christian-Albrechts-Universität<br>Schwanenweg 20<br>24105 Kiel
Kinderkrankenhaus auf der Bult<br>Janusz-Korczak-Allee 12<br>30173 Hannover	Frau Prof. Dr. Evelyn Kattner	Ethikkommission der Medizinischen Hochschule Hannover<br>Carl-Neuberg-Straße 1<br>30625 Hannover
Universitätskinderklinik<br>Perinatalzentrum Magdeburg<br>Leipziger Straße 44<br>39120 Magdeburg	Dr. Katrin Buder	Ethikkommission der Medizinischen Fakultät Otto-von-Guericke-Universität<br>Leipziger Straße 44/Haus 28<br>39120 Magdeburg

Zentrum für Kinder- und Jugendmedizin<br>Ernst-Moritz-Arndt-Universität<br>Soldmannstrasse 15<br>17487 Greifswald	Priv. Doz. Dr. Helmuth Küster	Ethikkommission an der Medizinischen Fakultät<br>der Ernst-Moritz-Arndt-Universität Greifwald<br>Fleischmannstraße 8<br>17487 Greifswald
Kinderklinik Kassel<br>Mönchebergstrasse 41-43<br>34125 Kassel	Dr. Dirk Müller	Ethikkommission der Landesärztekammer Hessen<br>Im Vogelsgesang 3<br>60488 Frankfurt/Main
Universitätsklinik für Kinder und Jugendliche<br>Liebigstraße 20a<br>04103 Leipzig	Frau Dr. Corinna Gebauer	Ethikkommission an der Medizinischen Fakultät der Universität Leipzig<br>Härtelstraße 16-18<br>04107 Leipzig
Kinderklinik St. Hedwig<br>Steinmetzstrasse 1-3<br>93049 Regensburg	Prof. Dr. Hugo Segerer	Ethik-Kommission der Medizinischen Fakultät der Universität Regensburg<br>Geschäftsstelle: Zentrum für Klinische Studien<br>Franz-Josef-Strauß Allee 11<br>93053 Regensburg
Kinderklinik Eutin<br>Ostholstein Kliniken<br>Janusstrasse 22<br>23701 Eutin	Dr. Jochen Reese	Ethikkommission der Ärztekammer Schleswig-Holstein<br>Bismarckallee 8-12<br>23795 Bad Segeberg
Medizinische Hochschule Hannover<br>Kinderklinik I<br>Carl Neuberg Straße 1<br>30625 Hannover	Dr. Dördelmann, Frau PD Dr. Bohnhorst	Ethikkommission der Medizinischen Hochschule Hannover<br>Carl-Neuberg-Straße 1<br>30625 Hannover
Asklepios Klinik Barmbek<br>Neonatologie<br>Rübenkamp 220<br>22291 Hamburg	Dr. Reinhard Laux	Ethik-Kommission der Ärztekammer Hamburg<br>Heinrich-Hertz-Straße 125<br>22083 Hamburg
Klinikum Südstadt<br>Abteilung für Neonatologie<br>Südring 81<br>19059 Rostock	Dr. D. Olbertz	Ethikkommission bei der Ärztekammer Mecklenburg-Vorpommern<br>Humboldstraße 6<br>18055 Rostock
Evangelisches Krankenhaus Bielefeld<br>Klinik für Kinder- und Jugendmedizin<br>Grenzweg 10<br>33617 Bielefeld	Frau Dr. Ursula Weller	Ethikkommission der Medizinischen Fakultät der Westfälischen Wilhelms-Universität Münster<br>und der Ärztekammer Westfalen-Lippe<br>Von-Esmarch-Straße 62<br>48149 Münster
Klinikum Mannheim<br>Kinderklinik<br>Theodor Kutzer-Ufer 1-3<br>68167 Mannheim	Dr. Thomas Schaible	Medizinische Ethikkommission II der Fakultät für Klinische Medizin Mannheim<br>der Ruprecht-Karls-Universität Heidelberg<br>Maybachstaße 14-16<br>68169 Mannheim
Universitätsklinikum des Saarlandes<br>Klinik für Kinder und Jugendmedizin<br>66421 Homburg	Prof. Dr. Ludwig Gortner	Ethik-Kommission bei der Ärztekammer des Saarlandes<br>Faktoreistraße 4<br>66111 Saarbrücken
Universitätskinderklinik Ulm<br>Unterer Brühl 3<br>89081 Ulm	Prof. Dr. Helmuth Hummler	Ethikkommission der Universität Ulm<br>Helmholtzstraße 20<br>89081 Ulm

3. Hypothesen und Ziele

Ziel der Studie ist die Etablierung eines Deutschen Frühgeborenen-Netzwerks, das

- o die Effekte genetischer und klinischer Risikofaktoren
- o die Einflüsse zentrumsspezifischer Behandlungsstrategien und
- o die Auswirkungen randomisierter Studien

auf die langfristige Entwicklung von Frühgeborenen mit einem Geburtsgewicht unter 1500 Gramm untersucht.

Klinisch wichtige Endpunkte sind dabei neben der Frühgeburtlichkeit selbst und den etablierten relevanten kurzfristigen Outcomeparametern (Tod, Hirnblutung, Wachstumsretardierung, Sepsis mit positiver Blutkultur, Operationen aufgrund von Komplikationen) die körperliche und geistige Entwicklung der Frühgeborenen im Alter von 2 und 6 Jahren. Im einzelnen werden folgende Hypothesen geprüft :

- Es existieren genetische Risikofaktoren für die oben beschriebenen Endpunkte, die im Rahmen der Studie identifiziert werden.
- Präklinische, klinische und soziale Faktoren haben einen größeren Einfluss auf die oben beschriebenen Endpunkte als genetische Faktoren.
- Standardisierte Rückmeldung an die an der Studie teilnehmenden Zentren zu ihren Therapien und Behandlungsergebnissen im Vergleich zu allen anderen und zu besonders erfolgreichen Zentren führen zu einem vermehrten Einsatz der von erfolgreichen Zentren verwendeten Behandlungsmethoden und im zeitlichen Verlauf zu einer Angleichung der Therapieschemata und zu verbesserten Behandlungsergebnissen aller Zentren.
- In randomisierten Studien untersuchte Medikamente und Techniken beeinflussen die langfristige körperliche und geistige Entwicklung der Frühgeborenen.

4. Stand der Forschung, eigene Vorarbeiten, Perspektiven der Studie

In allen Populationen werden etwa 1% aller Neugeborenen zu früh und mit einem Geburtsgewicht unter 1500 Gramm geboren. In Deutschland werden diese Frühgeborenen alle auf Frühgeborenenintensivstationen behandelt, und ihre Verlaufs- und Outcomedaten werden sehr genau registriert. Im Jahr 2005 verpflichtete der Gemeinsame Bundesausschuss alle neonatologischen Abteilungen der höchsten Versorgungsstufe zur entwicklungsneurologischen Nachuntersuchung aller Frühgeborenen mit einem Geburtsgewicht unter 1500 Gramm.

Im Gegensatz zu vielen anderen Ländern, wie z.B. den USA, existiert in Deutschland kein neonatologisches Forschungsnetzwerk, dass diese Daten sammelt, auswertet und die kurzfristigen und insbesondere die langfristigen Effekte einer zu frühen Geburt (und der damit verbundenen Therapien) untersucht.

Die Behandlungseffekte bestimmter Therapieformen lassen sich beim Frühgeborenen – im Unterschied zu vielen anderen Patientengruppen - oft erst nach Jahren an der dann erfolgten weiteren körperlichen und geistigen Entwicklung dieser Kinder beurteilen. Therapien, die aufgrund der kurzfristigen Verbesserung von Surrogatparametern eingeführt wurden, haben sich gerade in der Neonatologie immer wieder langfristig als schädlich erwiesen. Als jüngstes Beispiel sei die postnatale Anwendung von Dexamethason zur Prophylaxe und Therapie der bronchopulmonalen Dysplasie genannt. Bis vor wenigen Jahren wurde Dexamethason aufgrund von kurzfristigen positiven Effekten auf den Gasaustausch bei einem Großteil der Frühgeborenen mit bronchopulmonaler Dysplasie eingesetzt. Sorgfältige Nachuntersuchungen von Frühgeborenen im Klein- und Schulkindalter zeigten, dass die in randomisierten Studien mit Dexamethason behandelten Frühgeborenen einen niedrigeren Intelligenzquotienten und eine höhere Rate an Cerebralparesen aufwiesen. Heute wird Dexamethason nur noch in Einzelfällen bei Frühgeborenen verwendet.

Ziel des vorliegenden Protokolls ist die Etablierung eines bundesweiten Netzwerks mit einem besonderen Schwerpunkt auf der Untersuchung der langfristigen Entwicklung von Frühgeborenen. Im einzelnen bestehen für dieses Projekt folgende Vorarbeiten und Perspektiven:

Genetische Risikofaktoren

Im Jahr 2007 publizierte erfolgreiche Untersuchungen zu genetischen Risikofaktoren für Volkskrankheiten wie Myokardinfarkt und Diabetes mellitus zeigen, dass

genomweite Analysen in der Lage sind, genetische Risikofaktoren für häufige Erkrankungen zu identifizieren.
Für Frühgeborene wäre die Identifizierung genetischer Risikofaktoren von besonders großem Wert. Diese Kinder werden alle von Geburt an auf speziellen neonatologischen Intensivstationen betreut, so dass Kinder mit besonderen Risiken rasch identifiziert und gegebenenfalls prophylaktisch behandelt werden können.
Voraussetzung für die Identifizierung genetischer Risikofaktoren sind allerdings große Patientenkohorten, von denen ausreichend große DNA-Mengen zur Verfügung stehen. Unsere Arbeitsgruppe untersucht seit mehr als 10 Jahren genetische Risiken für Erkrankungen Frühgeborener mit einem Geburtsgewicht unter 1500 Gramm. Unsere DNA- und Datenbank umfasst zur Zeit mehr als 3000 Frühgeborene mit einem Geburtsgewicht <1500 g und 700 am Termin geborene Kinder (Kontrollen). Von der Mehrzahl dieser Kinder liegen uns zusätzlich auch maternale DNA-Proben vor. Unsere Frühgeborenen-DNA-Bank ist damit eines der weltweit größten Projekte in diesem Forschungsbereich.

Einfluss präklinischer und sozialer Faktoren

Es ist bekannt, dass bestimmte präklinische (z.B. Geburt aufgrund eines Amnioninfektionssyndroms) und soziale Faktoren (z.B. Herkunft, Schulbildung der Mutter) einen großen Einfluss auf die langfristige Entwicklung von Frühgeborenen haben. Wir erwarten, dass diese Faktoren – die nur sehr schwer zu beeinflussen sind – einen größeren Einfluss auf die langfristige Entwicklung der Frühgeborenen haben als die oben beschriebenen genetischen Faktoren und einen etwa vergleichbaren Einfluss wie die unten beschriebenen zentrumsspezifischen Therapien. Bislang existieren keine Studien, die die kombinierten Effekte genetischer, sozialer und klinischer Faktoren untersuchen.

Zentrumsspezifische Therapien

Im Bezug auf die Häufigkeit der oben angeführten Endpunkte bestehen große Unterschiede zwischen einzelnen neonatologischen Abteilungen. Auf die Analyse dieser Differenzen und entsprechende Rückmeldungen an die an der Studie teilnehmenden Zentren wurde deshalb besonderes Gewicht gelegt. Wir erwarten, dass zentrumsspezifische Faktoren einen etwa gleich großen Einfluss auf die kurz- und langfristige Entwicklung von Frühgeborenen haben wie präklinische und soziale Faktoren, dass sie sich aber wesentlich besser beeinflussen lassen (siehe unten).

Benchmarking

Alle an der Studie teilnehmenden Kliniken erhalten nach jedem Monitoring Zentrumsberichte, in denen die klinischen Daten des eigenen Zentrums den Daten aller anderen Zentren und den Daten der besonders erfolgreichen Zentren (in Bezug auf die oben genannten kurz- und langfristigen Endpunkte) gegenübergestellt werden. Da wir auch eine Vielzahl von Therapiedaten erheben, können Zentren mit ungünstigen Outcomedaten sich sehr rasch über Unterschiede der Therapie im Vergleich zu Zentren mit guten Outcomedaten (immer bezogen auf den jeweiligen Endpunkt) informieren. Ferner werden wir den Zentren die Möglichkeit geben, spezifische Suchabfragen für ihre eigenen Datensätze im Vergleich zu allen anderen Datensätzen durchzuführen.

Wir wissen aus unserem laufenden Projekt, dass die von uns erstellten Zentrumsberichte von vielen Kliniken bereits jetzt als Qualitätsindikatoren für die tägliche Arbeit genutzt werden.

Klinische Studien

Ferner hat das hier beschriebene Frühgeborenen-Netzwerk zum Ziel, die Planung und Durchführung von Arzneimittelstudien bei Frühgeborenen zu fördern. In den vergangenen Jahrzehnten wurden an Kindern, insbesondere an Frühgeborenen, kaum durch die pharmazeutische Industrie gesponsorte klinische Arzneimittelstudien durchgeführt. Dies hat zur Folge, dass für den Großteil der auf einer neonatologischen Intensivstation eingesetzten Arzneimittel keine arzneimittelrechtliche Zulassung besteht. Dieser Missstand wurde durch den Gesetzgeber erkannt und führte zu Änderungen des Arzneimittelrechts, die Studien an Kindern und Neugeborenen in besonderem Maße fördern. Wir möchten mit unserem Projekt diesen Prozess von der klinischen Seite her unterstützen und der pharmazeutischen Industrie, besonders aber auch an klinischen Studien interessierten Wissenschaftlern einen logistischen Rahmen bieten, der die Durchführung von Arzneimittelstudien an Frühgeborenen erleichtert. Das Netzwerk wird dabei selbst nicht als Sponsor oder Klinisches Koordinierungszentrum auftreten.

Die enge Zusammenarbeit der Zentren im Rahmen unseres bereits bestehenden Projektes hat bereits zur Etablierung erster Zusatzstudien geführt. Als Beispiele seien zwei prospektive randomisierte Arzneimittelstudien zur Wirksamkeit von unter Spontanatmung verabreichten Surfactant bei Frühgeborenen genannt. In die seit September 2007 laufende Studie zur Vermeidung der Beatmung von Frühgeborenen

(Avoid mechanical ventilation, EudraCT 2006-006912-31, ISRCTN 05025922, Förderung durch Chiesi Pharmaceuticals, Studienleitung PD Göpel/Prof. Herting) konnten bereits 50 Frühgeborene eingeschlossen werden. Eine weitere klinische Studie zur Vermeidung einer chronischen Lungenschädigung durch Surfactantgabe ohne Intubation (Förderung durch DFG/BMBF, Studienleitung Dr. Kribs/Prof. Roth) wird noch im laufenden Jahr beginnen.

5. Studiendesign

5.1. Studientyp

Prospektive Kohortenstudie in die etwa 25% aller in Deutschland geborenen Frühgeborenen mit einem Geburtsgewicht unter 1500 Gramm eingeschlossen und über einen Zeitraum von 6 Jahren bezüglich ihrer körperlichen und neurologischen Entwicklung nachbeobachtet werden.

Einschlusskriterien:

- Geburt <= 36+6 Schwangerschaftswochen
- Geburtsgewicht < 1500 Gramm

Ausschlusskriterium:

- Verlegung des Kindes aus einer anderen Kinderklinik.

5.2. Endpunkte

Daten zu jedem einzelnen Patienten werden zu 3 Zeitpunkten gesammelt:

1. Während des unmittelbaren postnatalen Aufenthaltes in der Klinik.
2. Bei einer ambulanten Wiedervorstellung im Alter von 2 Jahren.
3. Bei einer ambulanten Wiedervorstellung im Alter von 6 Jahren.

Folgende relevante Endpunkte sind zur Analyse vorgesehen:

- Während des stationären Verlaufes
    - Tod
    - Sepsis mit positiver Blutkultur
    - Hirnblutung (Gradeinteilung nach I-III, zusätzlich Parenchymbeteiligung)
    - Bronchopulmonale Dysplasie (Sauerstoffbedarf mit 36+0 SSW nach NIH-Konsensus-Definition)
    - Operation wegen
        a. Persistierendem Ductus arteriosus
        b. Nekrotisierender Enterokolitis
        c. Hydrozephalus (Shuntanlage)
        d. Frühgeborenenretinopathie (Laser-/Kryotherapie)
    - Pathologischer Hörtest
    - Körpergewicht, Körperlänge und Kopfumfang unter der 10. Perzentile (bei Aufnahme und bei Entlassung)
- Zum Zeitpunkt der Vorstellung mit 2 Jahren:

- -3 Tod
- -3 Körpergewicht, Körperlänge und Kopfumfang unter der 10. Perzentile
- -3 Bayley Scales for infant Development II (Deutsche Version) MIDII oder PIDII < 70

•3 Die letzte Wiedervorstellung ist im Alter von 6 Jahren vorgesehen. Zu diesem Zeitpunkt werden folgende Endpunkte bestimmt:

- -3 Tod
- -3 Körpergewicht, Körperlänge und Kopfumfang unter der 10. Perzentile
- -3 Die Kaufmann Assessment Battery for Children (K-ABC, Deutsche Version): < 70
- -3 Strengths and difficulties questionnaire (Modul des KIGGS-Surveys)
- -3 Untersuchung der motorischen Leistungsfähigkeit, Visusprüfung, Prüfung des Stereosehens, Blutdruckmessung (Module des KIGGS-Surveys)

Instrumente zur Erfassung der Endpunkte:

Die klinischen Endpunkte während des stationären Aufenthaltes werden durch die in unserer laufenden Studie verwendeten Erhebungsbögen dokumentiert. Die Bayley-Scales testen die kognitive und motorische Entwicklung von Kindern im Alter von 1-42 Monaten. Mit dem Kaufmann ABC werden Intelligenz und erworbene Fertigkeiten von Kindern im Alter von 2-12 Jahren getestet. Bei dem „Strengths and difficulties questionaire“ handelt es sich um einen Elternfragebogen zum Verhalten von Kindern und Jugendlichen der im Rahmen der KIGGS-Studie eingesetzt wurde. Die weiteren Module des KIGGS-Surveys wurden ebenfalls an einer Stichprobe von mehreren tausend Kindern normiert. Sie bestehen aus 10 Aufgaben, mit denen Koordination, Kraft und Beweglichkeit getestet werden, einer einfachen Prüfung des Stereosehens, einer Visusprüfung und einer Blutdruckmessung.

Alle als Endpunkte formulierten klinischen Items sind klar definiert und werden in internationalen Studien als Endpunkte verwendet. Die Tests und Erhebungsbögen sind ebenfalls standardisiert und an großen Gruppen von gesunden Kindern validiert. Auch sie werden (bis auf einzelne Module des KIGGS-Surveys) in internationalen Studien als Standard eingesetzt und entsprechen darüber hinaus den Empfehlungen der deutschen Fachgesellschaften für die Nachuntersuchung von Frühgeborenen.

5.3. Erhebung von Daten mit möglichem Einfluss auf die Endpunkte

Neben diesen Instrumenten zur Endpunktbestimmung werden im Rahmen der Studie eine Vielzahl von Daten erhoben, die möglicherweise einen Einfluss auf die Endpunkte haben könnten.

- Während des stationären Aufenthaltes:
  - Präklinische Daten (Dokumentationsbogen 1),
  - alle Therapien während des stationären Aufenthaltes (Dokumentationsbogen 2 + 3)
  - KIGGS-Elternfragebogen (Fragen zum sozialen Umfeld, Ernährung etc.)
- Bei der Wiedervorstellung mit 2 und 6 Jahren
  - Ärztlicher Fragebogen zu Krankheiten
  - KIGGS-Elternfragebogen (Fragen zum sozialen Umfeld, Ernährung etc.)

Bei Geburt und im Alter von 2 und 6 Jahren werden die an einer großen Gruppe von Kindern normierten Fragebögen der KIGGS-Studie eingesetzt. (Einzelheiten unter http://www.kiggs.de).

Die Dokumentation der Daten des stationären Aufenthaltes und zum Zeitpunkt der Wiedervorstellung mit 2 Jahren erfolgt durch die Studienzentren. Die Erhebung Daten zum Umfeld (KIGGS-Elternfragebögen) und die Untersuchung im Alter von 6 Jahren erfolgt durch Personal der Studienzentrale.

5.4. Angestrebte Fallzahlen

Da wir für genetische Risikofaktoren die geringsten Effektstärken erwarten, orientiert sich die Fallzahlberechnung unserer Studie an den für die Identifizierung genetischer Risikofaktor notwendigen Zahlen. Genomweite Untersuchungen an Erwachsenen zeigen, dass 500-2000 betroffene Patienten notwendig sind, um genetische Risikofaktoren für eine einzelne Erkrankung zu identifizieren. Da die Häufigkeit der oben beschriebenen Endpunkte bei Frühgeborenen mit einem Geburtsgewicht unter 1500 Gramm zwischen 3 und 20 % liegt, ist zur Identifizierung genetischer Risikofaktoren eine Zahl von 20.000 prospektiv in die Studie eingeschlossener Kinder notwendig.

Ziel der vorliegenden Studie ist der Einschluss und die Nachbeobachtung von 20.000 Frühgeborenen mit einem Geburtsgewicht unter 1500 Gramm in einem Zeitraum von 12 Jahren.

Für eine so hohe Fallzahl ist die dauerhafte Teilnahme von etwa 50 großen neonatologischen Abteilungen an der Studie vorgesehen. Uns ist bewusst, dass die Teilnahme an dieser Studie für die beteiligten Zentren einen zusätzlichen Aufwand bedeutet. Wir haben deshalb von Anfang an auf eine möglichst einfache Durchführbarkeit der Studie im klinischen Alltag geachtet. Ferner ergeben sich folgende Vorteile für teilnehmende Zentren:

- Regelmäßige Berichte ermöglichen einen Vergleich der eigenen Therapie- und Outcomedaten mit anderen Zentren.
- Durch die Teilnahme an der Studie ist die nach der Richtlinie des Gemeinsamen Bundesausschusses über „Maßnahmen zur Qualitätssicherung der Versorgung von Früh- und Neugeborenen" vom 20.9.2005 notwendige externe Infektionssurveillance erfüllt.
- Für die Nachverfolgung von Patienten werden wir eine Anschriftendatenbank einrichten, die durch die Studienzentrale gepflegt wird. Die teilnehmenden Zentren werden über Anschriftenänderungen ihrer Frühgeborenen informiert. Dies vereinfacht die Einbestellungen von Frühgeborenen zur 2-Jahres-Untersuchung nach GBA erheblich.
- Um die teilnehmenden Zentren zu entlasten, und um eine möglichst objektive Beurteilung des langfristigen Verlaufs zu ermöglichen, erfolgt die 6-Jahres-untersuchung durch Personal der Studienzentrale. Wie auch bei den kurzfristigen Outcomedaten, erhalten die Zentren Rückmeldungen über die durch sie betreuten Patienten im Vergleich zu allen anderen Zentren.
- Für die Dokumentation der Daten bis zur 2-Jahresuntersuchung erfolgt eine pauschale Vergütung pro abgeschlossenem Patient an die teilnehmenden Zentren.

5.5. Studiendurchführung

5.5.1. Einschluss von Studienzentren

Aufgrund der hohen erforderlichen Fallzahlen wurde die Studie als multizentrische bundesweite Studie geplant. Da die Studienzentrale erhebliche Ressourcen in die Betreuung der einzelnen Zentren investiert, ist es unser Ziel, möglichst große neonatologische Abteilungen für die Mitarbeit in dieser Studie zu gewinnen, da dadurch einerseits die Anzahl der notwendigen Patienten schneller erreicht wird, zum anderen aber auch die Ressourcen der Studienzentrale gezielter eingesetzt werden können. Neonatologische Abteilungen, die weniger als 40 Frühgeborene <1500 g im Jahr

betreuen, sollten nur in Ausnahmefällen in diese Studie eingeschlossen werden. Der Einschluss von Studienzentren erfolgt durch den Studienleiter.

5.5.2. Schulung der Mitarbeiter in den Zentren

Vor Beginn des Patienteneinschlusses in dem jeweiligen Zentrum erfolgt eine Mitarbeiterschulung in dem teilnehmenden Zentrum durch einen Arzt der Studienzentrale zu Zielen und Ablauf der Studie. Im Rahmen dieser Schulung werden dem teilnehmenden Zentrum 50 vorgepackte Versandtaschen zum Patienteneinschluss und 50 Behälter zur Asservierung der Nabelschnurproben zur Verfügung gestellt.

5.5.3. Einschluss von Patienten und Dokumentation der klinischen Daten

Die von der Studienzentrale bereitgestellten Versandtaschen zum Patienteneinschluss enthalten neben einem ausführlichen Aufklärungsbogen, einer Einverständniserklärung und einem Fragebogen zu den klinischen Basisdaten des Frühgeborenen 4 sterile Abstrichröhrchen, mit denen bei dem Frühgeborenen und der Mutter des Kindes je 2 Mundschleimhautabstriche durchgeführt werden. Der Fragebogen zu den klinischen Basisdaten (Dokumentationsbogen 1 von 3) wird – zusammen mit den Mundschleimhautabstrichen – an unser Labor geschickt. Ferner wird von jedem an der Studie teilnehmenden Frühgeborenen ein kurzes Stück Nabelschnur (unmittelbar nach der Geburt) bei minus 20 Grad Celsius eingefroren. Nach Entlassung des Kindes werden die Datenerhebungsbögen 2 und 3 (von denen einer mit dem Neonatalerhebungsbogen identisch ist) ausgefüllt und an die Studienzentrale geschickt oder gefaxt. Das Flussdiagramm auf der folgenden Seite fasst den Einschluss von Patienten zusammen.

Flussdiagramm für teilnehmende Studienzentren

***Einschlusskriterien*:**

Frühgeborenes <= 36 + 6 SSW

Geburtsgewicht < 1500 g

Das Kind wurde nicht aus einer anderen Kinderklinik verlegt

Einschlusskriterien erfüllt ⇓

Aufklärungs- und Einverständnisbogen an die Eltern aushändigen

Einverständnis erteilt ⇓

Entnahme von je 2 Mundschleimhautabstrichen bei Mutter und Frühgeborenen

Ausfüllen Datenerhebungsbogen (1/3)

Abheften des Einverständnisbogens im Studienordner

Versand von Mundschleimhautabstrichen und Datenerhebungsbogen 1/3

In Datenerhebungsbogen 2/3 und 3/3 wird zunächst nur der Name des Kindes und das Geburtsdatum eingetragen. Die Bögen werden in der Akte des Kindes abgeheftet und nach Entlassung ausgefüllt an die Studienzentrale versandt.

Zusätzlich zu den Mundschleimhautabstrichen wird von jedem an der Studie teilnehmenden Frühgeborenen möglichst unmittelbar nach der Geburt ein kurzes Stück Nabelschnur (das z.B. beim Kürzen der Nabelschnur im Rahmen der Erstversorgung anfällt) bei minus 20 Grad Celsisus eingefroren.
Die Nabelschnurreste werden im Rahmen des Monitorings von einem Arzt der Studienzentrale abgeholt, müssen also nicht versandt werden. In halbjährlichen Abständen werden die erhobenen Daten durch die Studienzentrale geprüft und alle teilnehmenden Zentren erhalten einen schriftlichen Bericht, der sowohl zu den klinischen Prozeduren als auch den Outcomedaten im Vergleich mit den anderen an der Studie teilnehmenden Zentren Stellung nimmt.

Die detaillierte Erhebung von verabreichten Medikamenten und durchgeführten Prozeduren (Datenerhebungsbogen 2/3) ermöglicht uns die Untersuchung des Wechselspiels zwischen exogenen Einflussfaktoren und bestimmten genetischen Prädispositionen, ist darüber hinaus aber auch für die teilnehmenden Zentren von hohem Interesse, da sich aus den regelmäßigen Berichten der Studienzentrale zum jeweils durchgeführten Standard-Management einzelner Zentren und dem damit erzielten klinischen Outcome wichtige Anregungen ergeben können. Voraussetzung für die Vergleichbarkeit der Daten ist jedoch eine regelmäßige Kontrolle der Datenqualität. Der Patienteneinschluss und die Dokumentation der klinischen Daten ist Aufgabe der jeweiligen Zentren, die Eingabe der Daten in eine Datenbank erfolgt (doppelt kontrolliert) in der Studienzentrale.

5.5.4. Datenbanken und Datenschutz

Um die Frühgeborenen über einen Zeitraum von 6 Jahren verfolgen zu können, wird von der Studienleitung ein umfangreiches „Tracking-System“ etabliert. Für die gesamte Studie werden 2 Datenbanken angelegt. In der ersten Datenbank befinden sich die persönlichen Daten der Frühgeborenen (Geburtsdatum, Name, Anschrift, betreuende Klinik etc.) inklusive der Anzahl der ausgefüllten und gemonitorten Dokumentationsbögen. Um Rückfragen an die Eltern bei verstorbenen Kindern zu vermeiden, wird auch der etwaige Tod eines Kindes in dieser Datenbank dokumentiert. Die klinischen Daten der Patienten sind in einer zweiten, pseudonymisierten Datenbank hinterlegt, die lediglich über eine Nummer mit der personenbezogenen Datenbank verknüpft ist. Eine Rückmeldung von Daten aus der pseudonymisierten Datenbank an die Eltern, die betreuende Klinik oder andere Personen ist ausgeschlossen, da sonst die Anonymität der Studienteilnehmer nicht mehr gewahrt wäre. Umgekehrt können allerdings die Daten der pseudonymisierten Datenbank durch im Verlauf der Nachuntersuchungen erhobene Daten ergänzt werden.

Zur Pflege der Anschriftendatenbank werden alle an der Studie teilnehmenden Frühgeborenen und ihre Eltern zum Geburtstag des Kindes von der Studienzentrale mit einer Glückwunschkarte angeschrieben und über den Verlauf der Studie informiert. Bei unzustellbaren Anschreiben erfolgt eine Anschriftenermittlung über die betreuende Klinik oder die Einwohnermeldeämter.

5.5.5. Dokumentation der Nachuntersuchungen

Aufgrund der Verpflichtung im Rahmen der Empfehlungen des Gemeinsamen Bundesausschusses für die Versorgung von Frühgeborenen werden alle Frühgeborenen im Alter von 18-24 Monaten durch die teilnehmenden Zentren nachuntersucht. Die empfohlene Untersuchungsmethode ist der Bayley II Score, der durch die Zentren erhoben wird. Ferner werden die Kinder gemessen und gewogen und es findet eine standardisierte Erhebung von Krankheiten, Operationen und Medikamentengaben statt (KiGGS Erhebungsbogen). Schließlich werden die Eltern, ebenfalls mittels eines standardisierten Erhebungsbogen (KiGGS-Elternfragebogen) befragt.

Im Alter von 5-6 Jahren erfolgt eine 2. Nachuntersuchung. Diese Nachuntersuchung erfolgt durch einen Arzt der Studienzentrale. Die für diesen Zeitpunkt vorgesehenen entwicklungsneurologischen Testmethoden sind der Kaufmann ABC und das „Strengths and difficulties questionnaire“. Ferner werden die Kinder gemessen und gewogen und es werden relevante Daten zu Krankheiten, Operationen und Medikamentengaben (KIGGS-Fragebögen) erhoben. Erneut werden die Eltern mittels eines standardisierten Elternfragebogens befragt (KiGGS-Elternfragebogen). Detaillierte Dokumentationsbögen für diesen Untersuchungszeitpunkt sind in der vorliegenden Version des Studienprotokolls noch nicht hinterlegt, da sie den aktuellen Empfehlungen der Fachgesellschaften zum Untersuchungszeitpunkt (d.h. in 5-6 Jahren) entsprechen sollen.

5.5.5. Monitoring und Qualitätskontrolle

Da die hier beschriebene Studie zur Gewinnung von Daten für die Durchführung prospektiver klinischer Studien dient, lehnt sich das Studiendesign an die Empfehlungen zur „*Good Clinical Practice*“ an. Dies bedingt ein entsprechendes Monitoring der teilnehmenden Zentren.

5.5.5.1. Monitoringprozeduren vor Einschluss eines Studienzentrums

Vor dem Einschluss eines Zentrums in die Studie werden Kliniken, die an einer Teilnahme Interesse haben, befragt. Dieser Fragebogen dient zum einen der Sicherung einer ausreichenden Zentrumsgröße. Im Hinblick auf klinische Arzneimittelstudie werden aber auch für Medikamentenstudien wichtige Zentrumseigenschaften (z.B. Möglichkeit der Durchführung von Ultraschalluntersuchung, Röntgenaufnahmen, Laboruntersuchungen) abgefragt.

5.5.5.2. Monitoring im Verlauf der Studie

Alle Studienzentren werden in halbjährlichen Abständen durch einen Arzt der Studienzentrale besucht. Inhalt des Monitorings ist die Überprüfung der Vollständigkeit der klinischen Daten anhand der Quellendaten (Patientenakten), die Überprüfung der Einschlussrate von Patienten, die die Einschlusskriterien der Studie erfüllen anhand der Quellendaten (in der Regel Stationsbuch) sowie eine zahlenmäßige Erhebung der Parameter Tod, Entbindungsmodus, Beatmung, laser- bzw. kryokoagulationspflichtige ROP, operationspflichtige NEC bzw. Ileumperforation, VP-Shuntanlage, Gestationsalter, Geburtsgewicht, Sepsis mit positiver Blutkultur, Operation eines persistierenden Ductus arteriosus, IVH-Grad, PVL, BPD und pathologischer Hörtest für alle Frühgeborenen, die die Einschlusskriterien der Studie zwar erfüllen, jedoch nicht in die Studie aufgenommen wurden. Die Dokumentation dieser Daten erfolgt in Form einer Zählliste und dient einer möglichst guten Vergleichbarkeit der einzelnen Zentren. Über jeden Besuch wird von dem jeweiligen Monitor ein schriftlicher Bericht verfasst, der in dem Studienordner des teilnehmenden Zentrums und in der Studienzentrale abgeheftet wird. Das Monitoring der Nachuntersuchungsdaten mit 2 Jahren erfolgt analog durch Mitarbeiter der Studienzentrale.

Nach Kontrolle der Daten durch den Monitor werden alle vollständigen Datensätze und die dazugehörigen DNA-Proben pseudonymisiert.

5.5.6. Probenverarbeitung und Laboruntersuchungen

Zu jedem an der Studie teilnehmenden Patienten gehören 5 einzeln abgenommene DNA-Proben: jeweils 2 Mundschleimhautabstriche von Mutter und Kind, die im Rahmen der Einsendung des ersten Dokumentationsbogens an die Studienzentrale verschickt werden, und ein kurzes Stück tiefgefrorene Nabelschnur, dass zunächst in den teilnehmenden Zentren gelagert und im Rahmen der Monitoringvisite vom Arzt der Studienzentrale abgeholt wird. Da die Qualität und Menge der Nabelschnur-DNA wesentlich höher ist als die Qualität der Mundschleimhaut-DNA, wird von den Mundschleimhautproben zunächst nur eine mütterliche DNA-Probe isoliert. Die übrigen Mundschleimhautabstriche werden eingefroren. Nach den Monitoringvisiten wird aus den tiefgefrorenen Nabelschnurproben für jedes Frühgeborene DNA isoliert und in 3 Portionen geteilt (1 Probe für genomweite Untersuchungen, 1 Probe für die selektive Untersuchung interessanter Polymorphismen, 1 Probe als Reserve und Kontrolle). Sollte von einzelnen Frühgeborenen keine Nabelschnur vorliegen, wird die DNA aus

den Mundschleimhautabstrichen isoliert und als Probe für die Untersuchung interessanter Polymorphismen eingefroren.

Genomweite Untersuchungen machen erst ab einer sehr hohen Fallzahl von etwa 500 von einer spezifischen Erkrankung (z.B. Sepsis) betroffener Frühgeborener einen Sinn, so dass wir erste genomweite Untersuchungen erst ab dem 2. Studienjahr planen. Im Vorfeld dieser genomweiten Untersuchungen werden wir Kandidatengen-Polymorphismen untersuchen, die in genomweiten Studien an Erwachsenen (z.B. Risikogene für Diabetes mellitus und ihr Einfluss auf die Häufigkeit einer postnatalen Insulintherapie bei Frühgeborenen) oder in Kandidatengen-Untersuchungen an Frühgeborenen prädiktiv für Erkrankungen des Frühgeborenen waren.

5.7. Fallzahlen und biometrisches Konzept

Genetische und Risikofaktoren

An Endpunkten für die ersten genomweiten Untersuchungen sind geplant:

- Frühgeburtlichkeit
- Late-onset-Sepsis mit positiver Blutkultur
- Hirnblutung des Frühgeborenen
- Wachstumsretardierung (Geburtsgewicht unter der 10. Perzentile)
- Mentale und psychomotorische Retardierung im Alter von 2 Jahren

Für alle diese Endpunkte erwarten wir aufgrund von Voruntersuchungen eine Häufigkeit von mindestens 20%, so dass wir für diese Erkrankungen mit hoher Wahrscheinlichkeit bereits nach 6 Jahren valide genetische Risikofaktoren identifizieren können. Ausreichend große Fallzahlen für seltenere Erkrankungen des Frühgeborenen (z.B. nekrotisierende Enterokolitis, operationspflichtiger Ductus arteriosus, laser/kryotherapiepflichtige Frühgeborenenretinopathie, einen pathologischen Hörtest im Neugeborenenalter etc.) dürften nach etwa 8-10 Jahren Projektlaufzeit vorliegen. Genetische Risikofaktoren für eine geistige Entwicklungsverzögerung oder Behinderung, die sich zuverlässig erst in der Nachuntersuchung im Alter von 6 Jahren bestimmen lässt, erwarten wir nach 9-12 Jahren Projektlaufzeit.

Als Kontrollgruppe werden wir für die genomweiten Untersuchungen eine Gruppe Frühgeborenen auswählen, bei denen es im klinischen Verlauf zu keinerlei Komplikationen gekommen ist, und die im Alter von 2 Jahren eine normale körperliche und geistige Entwicklung aufweisen, obwohl sie mit einem extrem niedrigen Geburtsgewicht (<1000 Gramm) und niedrigem Gestationsalter (< 29 Schwangerschaftswochen) geboren wurden. Aus allen Frühgeborenen, die diese Kriterien erfüllen, wird zufällig eine Kontrollgruppe von 1000 Kindern für genomweite Untersuchungen erstellt.

Hintergrund der Erstellung einer solchen Gruppe von Super-Frühgeborenen (d.h. keine Erkrankung trotz extrem hohen Krankheitsrisiko) ist die Erwartung, dass in dieser Gruppe Risikoallele seltener und protektive Allele gegen Erkrankungen von Frühgeborenen häufiger auftreten als in der Normalbevölkerung, während wir in der Gruppe von Frühgeborenen mit bestimmten Erkrankungen (z.B. Sepsis) eine Häufung von Risikoallelen und ein selteneres Auftreten von protektiven Allelen im Vergleich zur Normalbevölkerung erwarten. Insgesamt ergibt sich somit eine höhere Differenz der

Allelhäufigkeit, so dass prädiktive Polymorphismen leichter detektiert werden können. Vergleiche mit publizierten genomweiten Datensätzen von Gesunden sind ebenfalls vorgesehen.

6. Ethik

Das Studienprotokoll wird von allen Ethik-Kommissionen der an der Studie teilnehmenden Kliniken beraten. Die Studienzentrale ist die Klinik für Kinder und Jugendmedizin der Universität Lübeck. Das Votum der Ethikkommission der medizinischen Fakultät der Universität Lübeck findet sich im Anhang.

7. Kosten

Alle von uns durchgeführten Untersuchungen und Datenerhebungen sind sowohl für die teilnehmenden Studienzentren als auch für die an der Studie teilnehmenden Patienten kostenfrei.

8. Ausgewählte Publikationen unserer Arbeitsgruppe

Ahrens P, Kattner E, Köhler B, Härtel Ch, Seidenberg J, Segerer H, Möller J, Göpel W. Mutations of Genes Involved in the Innate Immune System as Predictors of Sepsis in Very Low Birth Weight Infants. Ped Res 2004, 55: 652-56
Commentary: Genetic Screening for Susceptibility to Infection in the NICU Setting. Ped Res 2004, 55: 546-48

Göpel W, Kim D, Gortner L. Prothrombotic mutations as a risk factor for preterm birth.
Lancet 1999; 353:1411-2

Göpel W, Gortner L, Kohlmann T, Schultz C, Möller J.Low prevalence of large intraventricular haemorrhage in VLBW-infants carrying the factor-V-Leiden or prothrombin-G20210A muatation
Acta paediatrica 2001; 90: 1021-24

Göpel W, Ludwig M, Junge AK, Kohlmann T, Diedrich K, Möller J. Positive selection pressure for the factor V Leiden mutation due to improved fetal implantation. Lancet 2001; 358: 1238-39

Göpel W, Kattner E, Seidenberg J, Kohlmann T, Segerer H, Möller J. The effect of the Val34Leu polymorphism in the factor XIII gene in infants with a birth weight below 1500 g
J Pediatr 2002; 140:688-92

Härtel Ch, Finas D, Ahrens P, Kattner E, Schaible Th, Müller D, Segerer H, Albrecht K, Möller J, Diedrich K, Göpel W. Polymorphisms of genes involved in innate immunity – association to preterm delivery. Mol Hum Reprod 2004; 10:911-5.

Härtel C, von Otte S, Koch J, Ahrens P, Kattner E, Segerer H, Möller J, Diedrich K, Göpel W. Polymorphisms of haemostasis genes and preterm delivery. Thromb Haemost 2005; 94;88-92

Göpel W, Härtel C, Ahrens P, König I, Kattner E, Kuhls E, Küster H, Möller J, Roth B, Segerer H, Wieg C, Herting E. Interleukin-6-174-genotype, sepsis and cerebral injury in very low birth weight infants. Genes Immun 2006; 7:65-8

Härtel C, König I, Köster S, Kattner E, Kuhls E, Küster H, Möller J, Müller D, Roth B, Segerer H, Wieg C, Herting E, Göpel W. Haemostasis genes and primary outcome of very-low-birth-weight-infants. Pediatrics 2006; 118:683-9

Göpel W. A spitting image of the lungs (Comment).
Pediatrics 2006, 117:2285-6.

Hartel C, Puzik A, Göpel W, Temming P, Bucsky P, Schultz C. Immunomodulatory effect of vitamin C on intracytoplasmic cytokine production in neonatal cord blood cells. Neonatology 2007; 91:54-60.

Härtel C, Schultz C, Herting E, Göpel W. Genetic association studies in VLBW infants exemplifying susceptibility to sepsis – recent findings and implications for future research. Acta Paediatr 2007 96:158-65.

Härtel C, Osthues I, Rupp J, Haase B, Röder K, Göpel W, Herting E, Schultz C. Characterization of the host inflammatory response to Staphylococcus epidermidis in neonatal whole blood. Arch Dis Child Fetal Neonatal Ed. 2008; (Epub ahead of print).

Spiegler J, Kattner E, Vochem V, Küster H, Möller J, Müller D, Kribs A, Segerer H, Wieg C, Nikischin W, v.d.Wense A, Gebauer C, Herting E, Göpel W. Very low birth weight infants as a model to study genetic influences on neonatal weight gain. J Pediatr Gastr Nutr 2008, 46:113-6.

Deutsches Frühgeborenen-Netzwerk
German Neonatal Network

Erklärung zur Teilnahme an der Studie
Declaration of Commitment of Participating Centers

Klinik:____
(Institution)

Name des teilnehmenden Arztes:______________________________
(Name of investigator)

Einschlusskriterien (inclusion criteria):
Frühgeborene mit einem (preterm infants with)

- Geburtsgewicht < 1500 Gramm (birth weight < 1500 grams)
- Gestationalter <=36+6 Schwangerschaftswochen (gestational age <= 36+6 weeks)

Ausschlusskriterium (exclusion criteria):

- Verlegung des Kindes aus einer anderen Kinderklinik (transfer from another hospital with Paediatric Department)

Anzahl der Patienten pro Jahr, die obige Kriterien erfüllen und in die Studie eingeschlossen werden können:
(Number of patients expected to be recruited for the study (per year) under the above mentioned criteria):

N= __________ **Patienten/Jahr** (patients/year)

Ich kenne das Protokoll für die oben genannte Studie. Mir ist bekannt, dass das Protokoll die Sammlung von DNA-Proben, die Dokumentation des klinischen Verlaufs und die Dokumentation der Nachuntersuchung im Alter von 2 Jahren (entsprechend den Empfehlungen des Gemeinsamen Bundesausschusses) vorsieht. Ich bin über die Höhe der vorgesehenen Dokumentationspauschale für jeden vollständig dokumentierten Patienten (50€) informiert.
(I declare to be informed about the study protocol.)

Es bestehen keine wirtschaftlichen oder andere konfligierenden Interessen der teilnehmenden Klinik oder Person im Zusammenhang mit der Studie.
(No conflicts of interests with regard to the study are declared.)

____

Unterschrift	(Name und Anschrift oder Stempel)
(Signature)	(Name and address)

Studie „*Deutsches Frühgeborenen-Netzwerk (German Neonatal Network, GNN)*"

Aufklärungsbogen für Eltern

Liebe Eltern,

Ihr Kind wurde mit einem Geburtsgewicht unter 1500 Gramm und weit vor dem errechneten Geburtstermin geboren. Wir wissen, dass die allermeisten Frühgeborenen, auch wenn sie mit einem so geringen Geburtsgewicht zur Welt kommen, nach einer den Eltern oft sehr lang erscheinenden Zeit, aus dem Krankenhaus entlassen werden können. Leider gibt es kaum Daten über die langfristige Entwicklung von sehr kleinen Frühgeborenen in Deutschland. Insbesondere weiß man nicht, welchen Einfluss die zu frühe Geburt selbst, aber auch die Therapie im Krankenhaus, die genetische Veranlagung des einzelnen Kindes und spätere Umweltfaktoren auf die langfristige körperliche und geistige Entwicklung von Frühgeborenen haben.

Wozu dient die Studie?

Wichtigstes Ziel unserer Untersuchung ist es, Faktoren zu finden, die einen günstigen Einfluss auf die langfristige Entwicklung von Frühgeborenen haben. Nur wenn man weiß, welche Form der Therapie, welche genetische Eigenart und welche Umweltfaktoren einen günstigen Einfluss auf die langfristige Entwicklung der Frühgeborenen haben, kann man diese Therapien und Umweltfaktoren gezielt einsetzen und vielleicht auch Medikamente entwickeln, die besonders günstige genetische Eigenschaften nachahmen. Umgekehrt kann man natürlich ungünstige Faktoren vermeiden. Unsere Studie dient also der zukünftigen besseren Behandlung von sehr kleinen Frühgeborenen. Die im Rahmen der Studie registrierten Daten werden ausschließlich zu diesem Zweck erhoben und verwendet.

Wie wird die Studie durchgeführt?

Von allen an unserer Studie teilnehmenden Frühgeborenen werden zu drei Zeitpunkten Daten erhoben: während des stationären Aufenthaltes nach der Geburt, im Alter von 2 Jahren und im Alter von 6 Jahren.

Während des stationären Aufenthaltes werden die wesentlichen klinischen Daten Ihres Kindes (z.B. sein Geburts- und Entlassungsgewicht) von den Ärztinnen und Ärzten registriert. Um auch eventuelle genetische Einflüsse auf die Entwicklung von Frühgeborenen untersuchen zu können, benötigen wir jeweils 2 Mundschleimhautabstriche von Mutter und Kind und einen kurzen Rest der kindlichen Nabelschnur. Die Abstriche werden mit einem sterilen Wattetupfer gemacht und bedeuten auch für sehr kleine Kinder keine Belastung. Der Nabelschnurrest fällt beim Kürzen des Nabels bei der ersten Versorgung des Kindes an und würde sonst verworfen werden. Aus dem verwendeten Material werden keine anderen Untersuchungen durchgeführt, und die Datenschutzbestimmungen werden eingehalten. Für die Studie finden zu keinem Zeitpunkt Blutentnahmen statt.

Im Alter von 2 Jahren wird die motorische Entwicklung Ihres Kindes getestet. Diese Untersuchung ist für alle Frühgeborenen mit einem Geburtsgewicht unter 1500 Gramm

empfohlen, es handelt sich als nicht um eine Untersuchung, die extra für unsere Studie durchgeführt wird. Im Alter von 6 Jahren ist schließlich eine Untersuchung der geistigen und körperlichen Entwicklung (inklusive Sehtest) vorgesehen. Die Nachuntersuchungen mit 2 und 6 Jahren finden in der Klinik statt, in der Ihr Kind von Anfang an betreut wurde, Sie haben also keine langen Anfahrten. Das Ergebnis der Nachuntersuchung wird sofort mit Ihnen besprochen.

Ferner werden wir Ihnen zu allen drei Zeitpunkten (also während Ihr Kind noch im Krankenhaus ist, im Alter von 2 Jahren und im Alter von 6 Jahren) einen Elternfragebogen zusenden. Diese Elternfragebogen wurde schon von mehr als 17000 Eltern beantwortet, deren Kinder keine Frühgeborenen sind. So wird es uns durch diese Studie möglich werden herauszufinden, ob sich Frühgeborenen bezüglich ihrer Ernährung, der Häufigkeit von Arztbesuchen etc. von nicht zu früh geborenen Kindern unterscheiden. Damit Sie diese Fragebögen in Ruhe ausfüllen können, werden wir sie als pseudonymisierte (d.h. nur mit einer Nummer gekennzeichnete) Bögen direkt an Ihre Privatadresse versenden.

Wer nimmt an der Studie teil und wie lange werden die Daten gespeichert?

Bei unserer Studie handelt es sich um die größte bisher in Deutschland durchgeführte Studie zur langfristigen Entwicklung von Frühgeborenen. An unserer Studie nehmen 50 neonatologische Abteilungen in ganz Deutschland teil. Wir planen, über einen Zeitraum von 10 Jahren etwa 20000 Frühgeborene in unsere Studie einzuschließen und rechnen damit, die anonymisierten Daten über einen Zeitraum von mindestens 18 Jahren zu speichern, da erst dann die letzen in die Studie eingeschlossenen Kinder nachuntersucht sind.

Wie werden die Daten geschützt?

Der Schutz der persönlichen Daten der an der Studie teilnehmenden Frühgeborenen ist für uns ganz besonders wichtig. Aus diesem Grund werden wir alle Daten nur pseudonymisiert speichern (d. h. alle personenbezogenen Daten wie Name und Anschrift der teilnehmenden Kinder werden durch eine Nummer ersetzt). Deshalb können wir Ihnen auch keine einzelnen Untersuchungsergebnisse der genetischen Untersuchungen mitteilen.

Damit wir Sie zu den Nachuntersuchungen rechtzeitig einladen können, möchten wir Sie um die Erlaubnis bitten, Ihre Anschrift zu speichern. Wir werden Ihre Anschrift ausschließlich mit der Sie betreuenden Kinderklinik bzw. dem Meldeamt abgleichen und an keinen Dritten weitergeben. Sowohl die Anschriftendatei als auch die Datei mit den klinischen Daten ist durch spezielle Kennwörter und Programme geschützt, so dass niemand Zugriff auf Ihre Daten hat.

Es ist uns besonders wichtig, Sie über den Verlauf unserer Studie zu informieren. Wir möchten Sie deshalb um Ihr Einverständnis bitten, dass wir Ihnen einmal im Jahr (zum Geburtstag Ihres Kindes) ein kurzes Informationsschreiben zusenden.

Nach der Untersuchung im Alter von 6 Jahren werden alle Daten vollständig anonymisiert, d.h. Ihre persönlichen Daten werden vollständig gelöscht.

Die Teilnahme an dieser Studie ist natürlich freiwillig, und falls Sie nicht an der Studie teilnehmen erwachsen Ihnen oder Ihrem Kind hieraus keinerlei Nachteile. Sie können natürlich

Ihre Teilnahme an der Studie auch zurückziehen. In diesem Fall werden alle Daten (persönliche und klinische) gelöscht und alle Proben vernichtet. Dies ist allerdings nur bis zur Untersuchung im Alter von 6 Jahren möglich, da zu diesem Zeitpunkt alle persönlichen Daten ohnehin vollständig gelöscht werden. Falls Sie Ihre Teilnahme an der Studie zurückziehen möchten schreiben Sie bitte an das „Deutsche Frühgeborenen Netzwerk, Klinik für Kinder- und Jugendmedizin der Universität Lübeck, 23538 Lübeck".

Wie werden die Ergebnisse der Studie veröffentlicht?

Grundsätzlich werden keine Daten zu einzelnen Kliniken oder einzelnen Kindern veröffentlicht oder an Dritte weitergegeben. Alle Kliniken, die an der Studie teilnehmen erhalten regelmäßige Berichte über die von ihnen betreuten Frühgeborenen. Um möglichst viele interessierte Ärztinnen und Ärzte, aber auch andere Interessierte, über die Ergebnisse unserer Arbeit zu informieren, werden wir die Gesamtergebnisse der Studie in wissenschaftlichen Fachzeitschriften und im Internet veröffentlichen.

Wie wird die Studie finanziert?

Die Studie wird ausschließlich durch öffentliche Fördermittel durch das Bundesministerium für Bildung und Forschung finanziert. Es ist möglich, dass sich in Zukunft eine kommerzielle Nutzung der Forschungsergebnisse ergibt, an deren Ertrag die an der Studie teilnehmenden Forscher und Universitäten beteiligt sind. Für diesen Fall verzichten Sie mit Ihrer Einwilligung auf finanzielle Ansprüche, die sich eventuell mit der Vermarktung der Untersuchungsergebnisse ergeben könnten.

Wer ist verantwortlich für den Inhalt und die Durchführung der Studie?

Viele Ärztinnen und Ärzte haben an den Inhalten dieser Studie mitgearbeitet. Der Studieninhalt wurde mit Vertretern der Fachgesellschaften für Neonatologie, Perinatologie und Sozialpädiatrie und Experten für die statistische Auswertung von Daten beraten. Darüber hinaus haben wir viele Eltern von Frühgeborenen zu unserem Studienvorhaben befragt und die Studie mit dem Bundesverband „Das frühgeborene Kind" e.V., der größten Vereinigung von Eltern Frühgeborener in Deutschland, abgestimmt. Für die Durchführung der Studie ist der Studienleiter Prof. Dr. Wolfgang Göpel (Klinik für Kinder- und Jugendmedizin, Universität Lübeck, 23538 Lübeck) verantwortlich. Tritt im Rahmen der Studiendurchführung ein Schaden auf, der den Studienteilnehmern durch das schuldhafte Verhalten eines Beschäftigten des Universitätsklinikums Schleswig-Holstein (UK-SH) zugefügt wurde, haftet die gesetzliche Haftpflicht des UK-SH.

Wir hoffen, durch diese Studie kleinen Frühgeborenen in Zukunft noch besser helfen zu können. Falls Sie zu der Studie noch Fragen haben, wenden Sie sich bitte an die/den Ihr Kind behandelnde(n) Ärztin/Arzt bzw. an einen der unterzeichnenden Ärzte der Studienleitung.

Ärztin/Arzt des teilnehmenden Zentrums

Prof. Dr. med. W. Göpel
(Studienzentrale Universitäts-
kinderklinik Lübeck)

Studie „*Deutsches Frühgeborenen-Netzwerk (German Neonatal Network, GNN)“*

Einverständniserklärung

Hiermit erkläre/n ich/wir mein/unser Einverständnis zur Teilnahme meines(r)/unseres(r) Kindes(r)

____ geb. am ____________________

an der Studie „Deutsches Frühgeborenen-Netzwerk (German Neonatal Network)“

Ich habe die schriftliche Patienteninformation zur oben genannten Studie erhalten, gelesen und verstanden. Ich wurde ausführlich – mündlich und schriftlich – über das Ziel und den Verlauf der Studie aufgeklärt.

Ich wurde darauf hingewiesen, dass die Teilnahme meines Kindes freiwillig ist und dass ich das Recht habe, diese jederzeit ohne Angabe von Gründen zu beenden, ohne das mir oder meinem Kind dadurch Nachteile entstehen.

Ich bin darüber aufgeklärt und einverstanden, dass die klinischen Verlaufsdaten meines Kindes und meine Anschrift an die Studienzentrale (Kinderklinik der Universität Lübeck) weitergeleitet werden und dass meine Anschriftendaten (z.B. im Fall eines Wohnortwechsels) mit der mein Kind behandelnden Klinik oder dem Einwohnermelderegister abgeglichen werden.

Ich bin darüber informiert, dass die entnommenen Proben ausschließlich in anonymisierter Form zur Erforschung von Einflussfaktoren für Erkrankungen des Frühgeborenen und Frühgeburtlichkeit genutzt werden und dass eine Befundmitteilung der genetischen Daten weder an mich noch an die mein Kind behandelnden Ärzte erfolgen kann.

Ich/wir hatte/n Gelegenheit, alle meine/unsere Fragen zu stellen, diese wurden zufriedenstellend und vollständig beantwortet.

____

Ort, Datum Unterschrift des/der Erziehungsberechtigten

____

Ort, Datum Aufklärender Arzt/Ärztin (Name) Aufklärender Arzt/Ärztin (Unterschrift)

Datenbogen bitte ausfüllen und zusammen mit je zwei Mundschleimhautabstrichen von Mutter und Kind an die Studienzentrale (Klinik für Kinder und Jugendmedizin, Priv. Doz. Dr. W. Göpel, Frühgeborenenstudie, 23538 Lübeck) senden.

Kinderklinik NN

Studie *„Deutsches Frühgeborenen-Netzwerk (German Neonatal Network, GNN)“*

Dokumentationsbogen bei Einschluss in die Studie (1/3)

Initialen Vorname/Nachname ____/_____
Geburtstag
Nabelschnurrest eingefroren?
£ ja £ nein

1. Geschlecht

£ weiblich
£ männlich

2. Mehrling

£ nein £ ja
falls ja:
Mehrling Nr. _____ von ______
Bei Zwillingen:
£ monozygot £ dizygot £ unklar

3. Geburt

Geburtsmodus:
£ spontan £ Sectio
APGAR 5 und 10 Minuten: ___ / ____
Nabelarterien-ph:______

4. Geburtsgewicht und Gestationsalter

__________ Gramm
errechneter Geburtstermin: ___. ___. _______
oder
Gestationsalter: _____Wochen + ____Tage

5. CRIB-Score

Maximaler BE in den ersten 12h: ___________
Minimaler Sauerstoff in den ersten 12h:_______
Maximaler Sauerstoff in den ersten 12h:_______
angeborene Fehlbildungen:
£ keine £ nicht lebensbedrohlich
£ akut lebensbedrohlich (ISTA, CHARGE, Fallot, Hydrops, Omphalozele, OI, VACTERL, Harnstoffzyklusdefekt, Lungenhypoplasie, Prune belly, polyzystische Nierendegeneration)

6. „Inborn“ (Geburt in Klinik mit neonatologischer Intensivstation)

£ nein £ ja

7. Lungenreifebehandlung vor der Geburt

£ nein £ ja
falls ja,**:**
£ Betamethason
£ Dexamethason
£ anderes Medikament und zwar ___________

8. Tokolysebehandlung vor der Geburt

£ nein £ ja
falls ja:
£ Magnesium
£ Fenoterol (Partusisten®)
£ Atosiban (Tractotile®)
£ anderes Medikament und zwar ____________

9. Antenatale Antibiotische Therapie

£ nein £ ja
falls ja:
£ Cephalosporine
£ Metronidazol (Clont®)
£ Ampicillin und andere Penicilline
£ Imipenem/Meropenem
£ sonstige______________________________

10. Ursache der zu frühen Geburt

£ unhemmbare Wehen
£ V.a. Amnioninfektionssyndrom
£ EPH-Gestose
£ HELLP
£ pathologisches CTG
£ Wachstumsretardierung und pathologische Doppler
£ Placentalösung
£ sonstige______________________________

11. Herkunftsland der Mutter

£ Deutschland
£ übriges Europa einschl. Russland
£ Naher Osten einschl. Türkei und Nordafrika
£ Asien
£ Afrika
£ sonstige______________________________

12. Alter der Mutter

_________Jahre

13. Vorausgegangene Geburten

£ nein £ ja
falls ja:
Frühgeburten (vor der 37+0 SSW)?
£ nein £ ja

Hiermit wird die Richtigkeit der Angaben bestätigt

Unterschrift (dokumentierende Ärztin/Arzt)

Datenbogen bitte nach Entlassung des Kindes ausfüllen und an die Studienzentrale (Klinik für Kinder und Jugendmedizin, Priv. Doz. Dr. W. Göpel, Frühgeborenenstudie, 23538 Lübeck) senden.

Kinderklinik NN

Studie „*Deutsches Frühgeborenen-Netzwerk (German Neonatal Network, GNN)*"

Dokumentationsbogen Behandlung (2/3)

Name des Kindes

Geburtstag

1. Prophylaktische Medikamente:

Vitamin K : £ oral £ i.m. £ i.v. £ s.c.
weitere:
£ Teicoplanin/Vancomycin zur Prophylaxe der grampositiven Sepsis
£ systemisch verabreichtes Fluconazol zur Prophylaxe von Pilzinfektionen
£ Ibuprofen £ Indomethacin zur Prophylaxe eines persistierenden Ductus arteriosus
£ Erythropoeitin zur Prophylaxe einer Frühgeborenenanämie
£ ____________________zur Prophylaxe von ____________________

2. Antibiotische Therapie

£ nein £ ja
falls ja:
£ Penicillin £ Ampicillin/Amoxicillin
£ Cefotaxim £ Imipenem
£ Gentamicin £ Tobramycin
£ Erythromycin £ Vancomycin
£ Teicoplanin £ Metronidazol
£ ____________________

3. Katecholamintherapie

£ nein £ ja
falls ja:
£ Dopamin £ Dobutamin
£ Noradrenalin £ Adrenalin
£ ____________________

4. Analgetika und Sedativa

£ nein £ ja
Falls ja:
£ Midazolam (Dormicum®) £ Diazepam
£ Morphin £ Fentanyl
£ ____________________

5. Surfactant

£ nein £ ja
falls ja, wie oft**:**
£ 1 x £ 2 x £ 3 x £ __ x
Surfactantgabe ohne Intubation £ ja £ nein

6. Diuretika

£ nein £ ja
falls ja:
£ Furosemid (Lasix®)
£ Hydrochlorothiazid
£ Spironolacton (Aldactone®)
£ ____________________

7. Inhalative Medikamente

£ nein £ ja
falls ja:
£ Salbutamol (Sultanol®)
£ Ipratropiumbromid (Atrovent®)
£ Budenosid (z. B. Pulmicort®)
£ ____________________

8. Transfusionen

Blut: £ keine £ 1 £ 2 £ 3 £ ___(n)
FFP: £ keine £ __ (n) **TK**: £ keine £ ___(n)

9. Atemanaleptische Medikamente

£ nein £ ja
falls ja:
£ Theophyllin £ Coffein
£ Doxapram £ ____________

10. Sonstige Medikamente

£ Insulin £ Vitamine (außer Vit. D)
£ Eisen £ ____________________
£ ____________________
£ ____________________

11. Wurden Medikamente wg. NW abgesetzt?

£ nein £ ja
falls ja:__________ wg.________________

12. Zentral-venöse Katheter

£ nein £ ja
falls ja:
£ Nabelvenenkatheter
£ Polyurethaneinschwemmkatheter
£ Siliconeinschwemmkatheter
Entfernung wegen Komplikationen??
falls ja, bitte Kathetertyp angeben**:**
£ Dislokation (______________________)
£ Katheterinfektion (______________________)
£ Thrombose (______________________)

13. Arterielle Katheter:

£ nein £ ja
falls ja: £ Nabel £ periphere Arterienkatheter
Kam es in Assoziation mit arteriellen Kathetern zu Extremitätennekrosen?
£ nein £ ja

14. Atemunterstützung:

£ nein £ ja
falls ja
- konventionelle Beatmung £ nein £ ja
- Hochfrequenzbeatmung £ nein £ ja
- mononasaler CPAP £ nein £ ja
- binasaler CPAP £ nein £ ja
  Pneumothorax £ nein £ ja

falls Pneumothorax bitte Beatmung/CPAP-Form zum Zeitpunkt des Pneus angeben:__________

15. Nahrungsaufbau

Beginn am ___. Lebenstag.
Vollständig oral am ___. Lebenstag

Hiermit wird die Richtigkeit der Angaben bestätigt Unterschrift (dokumentierende Ärztin/Arzt)

Datenbogen bitte nach Entlassung des Kindes ausfüllen und an die Studienzentrale (Klinik für Kinder und Jugendmedizin, Priv. Doz. Dr. W. Göpel, Frühgeborenenstudie, 23538 Lübeck) senden.

Kinderklinik NN

Studie „*Deutsches Frühgeborenen-Netzwerk (German Neonatal Network, GNN)*“

Dokumentationsbogen Verlauf (3/3)

Dieser Datenerhebungsbogen entspricht den Punkten 5/6, 14-16 und 19-24 des bundeseinheitlichen Neonatalerhebungsbogens. Alternativ kann deshalb statt diesem Blatt auch ein Ausdruck des Bogens verwendet werden. Bitte achten Sie in diesem Fall jedoch auf das vollständige Ausfüllen der oben genannten Felder und insbesondere auf die korrekte Angabe etwaiger operativer Prozeduren (siehe 9.)

Name des Kindes

Geburtstag

1. Gestationsalter:

errechneter Geburtstermin: ___. ___. _______
oder
Gestationsalter: _____Wochen + ____Tage

2. Aufnahme

Körpertemperatur bei Aufnahme:______ ° C
Gewicht bei Aufnahme: ___________Gramm
Länge: ________cm
Kopfumfang: ________cm

3. Sauerstoffzufuhr

£ nein £ ja
falls ja:
Beginn: ___..___ ______
endgültige Beendigung:___ ___ ________

4. Maschinelle Beatmung (nicht CPAP)

£ nein £ ja
falls ja, Beginn: ___. ___. ______
endgültige Beendigung:___. ___. ________
endg. Beendigung jegl. Atemhilfe:___. ___. ____

5. Sepsis

£ nein £ ja
falls ja, Beginn: ___. ___. ________
Erreger in Blut, Liquor oder Urin:

kein Keimnachweis (00)	£
Staphyl. aureus (31)	£
Staphyl. epidermidis (32)	£
Streptokok. Gruppe B (33)	£
Strep. Gruppe D (Enterokokken) (34)	£
Strep. Andere Gruppen (35)	£
Pneumokokken (36)	£
E. coli (37)	£
Klebsiella (38)	£
Enterobakter (39)	£
Serratia (40)	£
Proteus (41)	£
Pseudomonas aeruginosa (42)	£
Listeria monocytogenes (43)	£
Anaerobe Kokken (44)	£
Bacteroides-Arten (45)	£
Candida (46)	£
andere Erreger (47)	£

Typische klinische Symptomatik £ nein £ ja
Höchstes gemessenes CRP: ____mg/dl
Höchster ermittelter I/T.Quotient: ______
wenn 2. Sepsis: Erreger lt. Kat.: ______
wenn 3. Sepsis: Erreger lt. Kat.: ______

6. Schädelsonogramm

£ nein £ ja
falls ja:
IVH: £ nein £ ja
Falls ja: max. Stadium

Grad 1: subependymal	£
Grad 2: < 50% Ventrikellumen	£
Grad 3: > 50% Ventrikellumen	£
Grad 4: intraparenchymatös	£

Periventrikuläre Leukomalazie £ nein £ ja

7. Ophthalmologische Untersuchung

£ nein £ ja
wenn ja, Retinopathie: £ nein £ ja
wenn ja, max. Stadium:______

8. apparatives Hörscreening

£ nein £ ja
falls ja: £ auffällig £ unauffällig

9. Operationen

operative Ligatur eines PDA	£ nein	£ ja
Laser- bzw. Kryotherapie bei ROP	£ nein	£ ja
abdominelle Operation bei NEC	£ nein	£ ja
abdominelle Operation bei fokaler intestinaler Perforation	£ nein	£ ja
Anlage eines VP-Shunts	£ nein	£ ja
inguinale Herniotomie	£ nein	£ ja
sonstige Operationen	£ nein	£ ja

____

10. Entlassung:

Gewicht bei Entlassung: ___________Gramm
Länge: ________cm
Kopfumfang: ________cm

Aufenthalt beendet am ____ ____ ________
Entlassung

Nach Hause	£
Geburtshilfe	£
andere Klinik	£
Tod	£

wenn verstorben: Ursache:_________________

11. weitere relevante Diagnosen (ICD10):

_________ _________ _________
_________ _________ _________
_________ _________ ________

Hiermit wird die Richtigkeit der Angaben bestätigt. Unterschrift (dokumentierende Ärztin/Arzt)

Definitionen zu den Erhebungsbögen 1-3 (eingegangen wird nur auf nicht selbst erklärende Items):

Bogen 1

5. Lungenreifebehandlung vor der Geburt:
Hier ist „ja“ anzukreuzen auch falls nur eine Gabe von Glucokortikoiden an die Mutter erfolgte, d.h. die Lungenreifebehandlung muss nicht abgeschlossen sein.
Die Lungenreifebehandlung darf auch mehrere Wochen zurückliegen (auch in diesem Fall ist „ja“ anzukreuzen).

6. Tokolyse:
Im Gegensatz zur Lungenreifebehandlung werden nur Tokolysen, die in den letzten 24 Stunden vor der Geburt eingesetzt wurden, dokumentiert.
Es ist allerdings nicht erforderlich, dass die Tokolyse über 24 h gegeben wurde. Eine einmalige Gabe, bzw. der Beginn einer Dauerinfusion mit Mg oder Fenoterol wird immer als „ja“ dokumentiert.
Eine vor Tagen oder Wochen gegebene Tokolyse wird nicht dokumentiert.
Eine etwaige orale Gabe von Magnesium wird ebenfalls nicht dokumentiert.

7. Antibiotika: Wie bei der Tokolyse werden nur die in den letzten 24 h vor der Geburt eingesetzten Antibiotika dokumentiert, diese allerdings auch bei nur einmaliger Gabe.

8. Ursachen der zu frühen Geburt
Hier dürfen mehrere Items angekreuzt werden. Grundsätzlich sollten alle Items angekreuzt werden, die mit zur Frühgeburt beigetragen haben.

> Beispiel 1: Frühgeburt bei EPH-Gestose, Wachstumsretardierung und pathologischen Dopplern: Hier wird sowohl EPH-Gestose als auch Wachstumsretardierung und pathologische Doppler dokumentiert
> Beispiel 2: Frühgeburt wegen EPH-Gestose (mütterliche Indikation): auch bei gleichzeitig bestehender Wachstumsretardierung würde hier nur EPH-Gestose kodiert.

10. Vorausgegangene Geburten: Hier werden nur Geburten (definiert als Totgeburten über 500g Geburtsgewicht oder Lebendgeburten) als „ja“ definiert. Aborte in der Frühschwangerschaft werden nicht als vorausgegangene Geburt gewertet.

Bogen 2:
Grundsätzlich soll durch diesen Bogen die Behandlung des einzelnen Patienten möglichst vollständig dokumentiert werden. Im Vordergrund stehen dabei allerdings die im jeweiligen Zentrum angewandten Therapien, d.h. Medikamente, die in einer anderen Klinik (in die das Kind z.B. kurzfristig wegen einer Operation verlegt wurde) gegeben wurden, werden nicht dokumentiert. Ebenso werden Medikamente, die das Kind in anderen Abteilungen erhält (z.B. Inhalationsnarkotika während einer Operation) nicht dokumentiert. Findet eine Operation auf der Neugeborenenintensivstation statt und wird das Kind während der OP von diesem Team verantwortlich betreut, so werden die gegebenen Medikamente allerdings dokumentiert.
Grundsätzlich löst bereits die einmalige Gabe eines Medikamentes die entsprechende Dokumentation aus (z.B. einmalige Gabe von Fentanyl zur Intubation = Fentanyl „ja“).
Das Item „vollständiger oraler Nahrungsaufbau“ ist definiert als der Lebenstag, an dem die enterale Nahrungsmenge mindestens 150 ml/kg Körpergewicht beträgt.

Bogen 3:
Die Definitionen dieses Bogens folgen dem Neonatalerhebungsbogen. Dies ist insbesondere für das Item 5 (maschinelle Beatmung) von Bedeutung: CPAP-Zeiten werden hier nur bei vorangegangener maschineller Beatmung kodiert (als „endgültige Beendigung jeder Atemhilfe“). Falls ein Kind primär mit CPAP versorgt wurde, wird dies nur auf dem Bogen 2 dokumentiert (allerdings ohne Dauer der CPAP-Anwendung).

University of Lübeck

UNIVERSITY CLINICS Schleswig-Holstein (UKSH)
Clinic for Pediatrics and Adolescent Medicine

Study protocol 1/2008

Version 2 / 14th of June, 2008

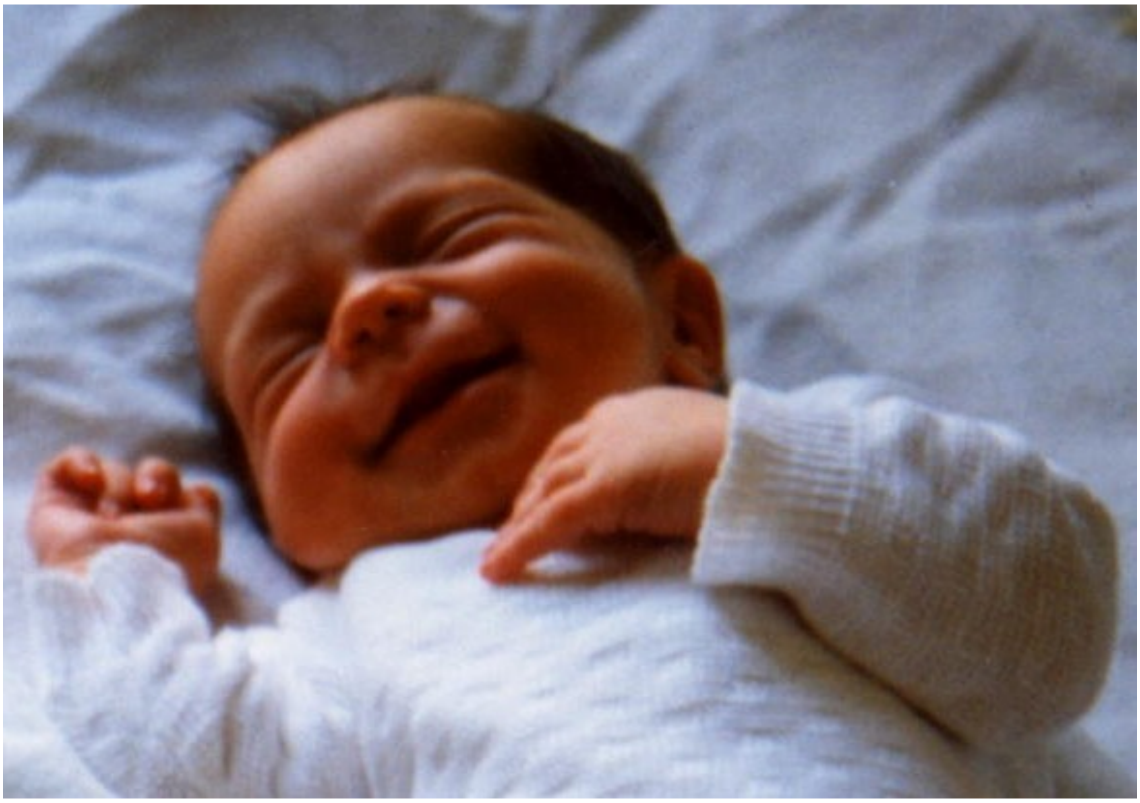

Deutsches Frühgeborenen-Netzwerk

German Neonatal Network (GNN)

Responsible coordinator:
Prof. Wolfgang Göpel and Prof. Egbert Herting, University of Lübeck
University Clinics Schleswig-Holstein
Clinic for Pediatrics and Adolescent Medicine
Ratzeburger Allee 160, D-23538 Lübeck
Tel.: +49-(0)451-500-2555
Fax: +49-(0)451-500-2590
Email: wolfgang.goepel@uksh.de

Content

		Page
1.	Summary	3
2.	Project management	5
3.	Hypotheses and aims	7
4.	State-of-the-art, own preliminary work and perspectives of the study	7
5.	Study design	9
6.	Ehtics	18
7.	Costs	18
8.	Selected publications of the research group	18
9.	Declaration of participating clinics	19
10.	Information sheet for parents	20
11.	Consent form	23
12.	Documentation sheet 1/3	24
13.	Documentation sheet 2/3	26
14.	Documentation sheet 3/3	27
15.	Definitions for the documentation sheets	28
16.	Parent questionnaire (6a follow-up)	30

1. Summary

The biggest fraction of mortality and morbidity in newborn children in Germany is caused by diseases in a small subgroup of children: preterm deliveries with a birth weight below 1,500 g. The most important goal for all persons in care of preterm babies is the undisturbed physical and cognitive development of these children, which usually is assessible at the age of 5-6 years. Unfortunately, in Germany no neonatological network exists to systematically analyze the determinants of the long-term development of preterm babies. Hence, the main aim of this study protocol is the establishment of such a German neonatal network (GNN), that

- analyzes the effects of genetic and clinical risk factors,
- the impact of center-specific treatment strategies, and
- the influence of randomized studies on the long-term development of preterm babies with a birth weight below 1,500 g.

A number of factors support this proposal:

- In 2005, the *Gemeinsame Bundesausschuss* (Joint Committee of the Federal Government) obliged all neonatal hospital departments to include specific follow-up examinations of all preterm babies with a birth weight of below 1,500 g at the age of 2 years.
- The 2007 Children and Youth Survey (Kinder- und Jugendgesundheits-Survey (KiGGS), which characterized a representative sample of 17,641 German children aged 0-17 years, serves as a unique reference data pool for the development of preterm babies and further provides a toolbox of validated measures for children's health.
- In 2007, genome-wide studies in adults have for the first time shown that a reliable identification of risk factors for common diseases has become technically possible.

Our study group has since than more than 10 years been working on describing genetic risk factors for neonatal pathologies. Our DNA data base comprises mote that 10,000 DNA samples of preterm babies with a birth weight below 1,500 g. It is one of the largest of its kind world-wide.

We expect that environmental factors (such as different treatment regiments in different neonatal departments, social background etc.) will have stronger effects on the long-term development of preterm babies than genetic determinants. Within the framework of this protocol we not only aim at studying the genetic regulation, but also the influence of center-specific treatment paradigms, of social environments and of novel therapeutic approaches – such as the application of surfactant without intubation – on the long-term development of preterm babies.

The main aim of the study is the prospective documentation of clinical data of 20,000 preterm babies with a birth weight of below 1,500 g starting from birth on and including the collection of DNA samples and the assessment of physical and cognitive development at the age of 2 to 6 years.

We hope that this – with regard to size so far unique study in German neonatology – will enable us to significantly improve the long-term development of our small patients.

2. Project management and participating clinics

**Project management**		
**Name**	**Clinic/institute**	**Function**
Prof. Dr. Wolfgang Göpel	Klinik für Kinder- und Jugendmedizin, Universität Lübeck	Principal investigator, responsible for design, management, coordination, and data analysis
Prof. Dr. Egbert Herting	Klinik für Kinder- und Jugendmedizin, Universität Lübeck	Study coordinator, Member of the council of the German Society for Neonatology and Pediatric Intensive Care (GNPI)
Prof. Dr. Andreas Ziegler	Universität Lübeck, Institut für Medizinische Biometrie und Statistik	Responsible for quality control, data management, and biometrics
Prof. Dr. Peter Nürnberg	Cologne Center for Genomics	Genome-wide SNP genotyping
Prof. Dr. Jeanette Erdmann	Univeristät Lübeck, Institut für Kardiogenetik	Genotyping (confirmation of associated SNPs)
**Project advisory board**		
Priv. Doz. Dr. Heike Rabe	Brighton and Sussex University Hospitals NHS Trust, UK	Council member of the European Society of Pediatric Research (ESPR)
Prof. Dr. Jens Möller	Kinderklinik Saarbrücken	Council member European Society of Neonatology and Pediatric Intensive Care (ESPNIC)
Prof. Dr. Dieter Wolke	University of Warwick, UK	Specific scientific expertise in preterm neonatal studies (e.g. EPICURE)
Dr. Bärbel-Maria Kurth	Robert-Koch Institut, Berlin	Specific scientific expertise in survey studies in children (e.g. KiGGS)
Prof. Dr. Ute Thyen	Universitätskinderklinik Lübeck	Council member of the German Society for Social Pediatrics and Youth Medicine (DGSJ)
Prof. Dr. Ulrich Gembruch	Universität Bonn, Klinik für Frauenheilkunde und Geburtshilfe	Council member of the German Society for Perinatal Medicine (DGPM)
Prof. Dr. Dr. Heiner Raspe	Universität Lübeck, Institut für Sozialmedizin	Specific scientific expertise in medical care research
Silke Mader	EFCNI	Council member of the European Foundation for the Care of Newborn Infants

Preliminary list of participating clinics

Clinic	PI at the participating clinic	Ethical commission
Klinik für Kinder und Jugendmedizin<br>UKSH Campus Lübeck<br>Ratzeburger Allee 160<br>23538 Lübeck	Prof. Dr. W. Göpel<br>Prof. Dr. Egbert Herting	Ethik-Kommission der Medizinischen Fakultät<br>Universität Lübeck<br>Ratzeburger Allee 160<br>23538 Lübeck
Altonaer Kinderkrankenhaus<br>Bleickenallee 38<br>22763 Hamburg	Dr. Axel v.d.Wense	zur Weiterleitung an die Ethikkommission<br>Prof. Dr. Frank Riedel<br>Ärztlicher Direktor des Altonaer Kinderkrankenhauses<br>Bleickenallee 38<br>22763 Hamburg
Klinik für Kinder und Jugendmedizin<br>Winterberg 1<br>66119 Saarbrücken	Prof. Dr. Jens Möller	zur Weiterleitung an die Ethikkommission<br>Prof. Dr. Jens Möller<br>Chefarzt der Klinik für Kinder- und Jugendmedizin<br>Winterberg 1<br>66119 Saarbrücken
Klinik für Allgemeine Pädiatrie Moorenstr. 5<br>40225 Düsseldorf	Prof. Dr. Thomas Höhn	Prof. Dr. Hans Gerd Lenard Vorsitzender der Ethikkommission der Heinrich Heine Universität<br>Moorenstr. 5<br>40225 Düsseldorf
Klinik für Kinder- und Jugendmedizin Am Hasenkopf 1<br>63739 Aschaffenburg	Prof. Dr. Christian Wieg	Ethik-Kommission der Medizinischen Fakultät<br>Josef Schneider Str. 2, D7 97080 Würzburg
Klinik und Poliklinik für Allgemeine Kinderheilkunde<br>Joseph-Stelzmann Str. 9<br>50924 Köln	PD Dr. Angela Kribs<br>Prof. Dr. Bernhard Roth	Ethik-Kommission der Medizinischen Fakultät<br>Joseph-Stelzmann-Str. 20<br>Forum/Gebäude 42<br>50931 Köln
Universitätskinderklinik Bochum<br>St. Josefs-Hospital<br>Alexandrinenstraße 5<br>44791 Bochum	Dr. Norbert Teig	Ethikkommission der Medizinischen Fakultät Ruhr-Universität<br>Bürkle-de-la-Camp Platz 1<br>44789 Bochum
Universitätskinderklinik Schleswig- Holstein<br>Klinik für Allgemeine Pädiatrie<br>Schwanenweg 20<br>24105 Kiel	PD Dr. Werner Nikischin	Ethikkommission der Medizinischen Fakultät Christian-Albrechts-Universität<br>Schwanenweg 20<br>24105 Kiel
Kinderkrankenhaus auf der Bult Janusz-Korczak-Allee 12<br>30173 Hannover	Frau Prof. Dr. Evelyn Kattner	Ethikkommission der Medizinischen Hochschule Hannover<br>Carl-Neuberg-Straße 1<br>30625 Hannover
Universitätskinderklinik<br>Perinatalzentrum Magdeburg<br>Leipziger Straße 44<br>39120 Magdeburg	Dr. Katrin Buder	Ethikkommission der Medizinischen Fakultät Otto-von-Guericke-Universität<br>Leipziger Straße 44/Haus 28<br>39120 Magdeburg

Zentrum für Kinder- und Jugendmedizin Ernst-Moritz-Arndt-Universität Soldmannstrasse 15 17487 Greifswald	Priv. Doz. Dr. Helmuth Küster	Ethikkommission an der Medizinischen Fakultät der Ernst-Moritz-Arndt-Universität Greifwald Fleischmannstraße 8 17487 Greifswald
Kinderklinik Kassel Mönchebergstrasse 41-43 34125 Kassel	Dr. Dirk Müller	Ethikkommission der Landesärztekammer Hessen Im Vogelsgesang 3 60488 Frankfurt/Main
Universitätsklinik für Kinder und Jugendliche Liebigstraße 20a 04103 Leipzig	Frau Dr. Corinna Gebauer	Ethikkommission an der Medizinischen Fakultät der Universität Leipzig Härtelstraße 16-18 04107 Leipzig
Kinderklinik St. Hedwig Steinmetzstrasse 1-3 93049 Regensburg	Prof. Dr. Hugo Segerer	Ethik-Kommission der Medizinischen Fakultät der Universität Regensburg Geschäftsstelle: Zentrum für Klinische Studien Franz-Josef-Strauß Allee 11 93053 Regensburg
Kinderklinik Eutin Ostholstein Kliniken Janusstrasse 22 23701 Eutin	Dr. Jochen Reese	Ethikkommission der Ärztekammer Schleswig-Holstein Bismarckallee 8-12 23795 Bad Segeberg
Medizinische Hochschule Hannover Kinderklinik I Carl Neuberg Straße 1 30625 Hannover	Dr. Dördelmann, Frau PD Dr. Bohnhorst	Ethikkommission der Medizinischen Hochschule Hannover Carl-Neuberg-Straße 1 30625 Hannover
Asklepios Klinik Barmbek Neonatologie Rübenkamp 220 22291 Hamburg	Dr. Reinhard Laux	Ethik-Kommission der Ärztekammer Hamburg Heinrich-Hertz-Straße 125 22083 Hamburg
Klinikum Südstadt Abteilung für Neonatologie Südring 81 19059 Rostock	Dr. D. Olbertz	Ethikkommission bei der Ärztekammer Mecklenburg-Vorpommern Humboldstraße 6 18055 Rostock
Evangelisches Krankenhaus Bielefeld Klinik für Kinder- und Jugendmedizin Grenzweg 10 33617 Bielefeld	Frau Dr. Ursula Weller	Ethikkommission der Medizinischen Fakultät der Westfälischen Wilhelms-Universität Münster und der Ärztekammer Westfalen-Lippe Von-Esmarch-Straße 62 48149 Münster
Klinikum Mannheim Kinderklinik Theodor Kutzer-Ufer 1-3 68167 Mannheim	Dr. Thomas Schaible	Medizinische Ethikkommission II der Fakultät für Klinische Medizin Mannheim der Ruprecht-Karls-Universität Heidelberg Maybachstaße 14-16 68169 Mannheim
Universitätsklinikum des Saarlandes Klinik für Kinder und Jugendmedizin 66421 Homburg	Prof. Dr. Ludwig Gortner	Ethik-Kommission bei der Ärztekammer des Saarlandes Faktoreistraße 4 66111 Saarbrücken
Universitätskinderklinik Ulm Unterer Brühl 3 89081 Ulm	Prof. Dr. Helmuth Hummler	Ethikkommission der Universität Ulm Helmholtzstraße 20 89081 Ulm

3. Hypotheses and aims

The main goal of the study is the establishment of a German Neonatal Network (GNN) that analyses

- the effects of genetic risk factors,
- the effects of center-specific treatment strategies, and
- the results of randomized studies

on the long-term development of preterm babies born with a weight of less than 1,500 g. Clinically important endpoints are the preterm birth itself and the established relevant short-term outcome parameters (death, cerebral hemorrhage, growth retardation, sepsis with positive blood culture, surgeries due to complications), the physical and mental development of preterm infants aged 2 and 6 years. In detail, the following hypotheses are examined:

- There are genetic risk factors for the endpoints described above that are identified in the study.
- Preclinical, clinical and social factors have a greater impact on the endpoints described above as genetic factors.
- Standardized feedback to the study's participating hospitals on their treatment and treatment outcomes compared to others and, particularly, other highly successful centers will result in increased application of treatments used by successful centers, and, in the long run, in therapeutic harmonization and improved treatment outcomes across all centers.
- Drugs and techniques studied in randomized trials affect the long-term physical and mental development of preterm babies.

4. State-of-the-art, own preliminary work, and perspectives of the study

In all populations about 1% of all newborns are born pretermly and with a birth weight below 1,500 grams. In Germany, these preterm infants are all treated in tertiary level neonatal intensive care units, and their course and outcome data are very accurately recorded. In 2005, the Joint Committee of the Federal Government obliged all neonatal departments of the highest care level to include developmental neurological follow-up examinations of all preterm babies with a birth weight below 1,500 grams. In contrast to many other countries, such as the US, there is no neonatological research network in Germany that collects and analyzes these data and examines the short-term and, in particular, the long-term effects of preterm birth (and associated therapies). The treatment effects of certain forms of therapy in preterm babies – unlike in many other patient groups – can be evaluated only years later by assessing the successful physical and mental development of these children. Therapies that have been introduced because of a short-term improvement of surrogate parameters have repeatedly proven to be harmful in the long term, especially in neonatology. As a recent example, the postnatal use of dexamethasone for the prophylaxis and treatment of bronchopulmonary dysplasia

should be mentioned. Until a few years ago, dexamethasone was used in a large proportion of preterm babies with bronchopulmonary dysplasia for its short-term positive effects on gas exchange. Careful follow-up studies in preterm babies in infancy and school age showed that preterm infants treated in randomized trials with dexamethasone showed a lower intelligence quotient and a higher rate of cerebral palsy. Today, dexamethasone is only used occasionally in preterm babies. The aim of the present protocol is to establish a nationwide network with a special focus on studying the long-term development of preterm babies. The following preliminary data and perspectives exist:

Genetic risk factors

Studies on genetic risk factors for common diseases such as myocardial infarction and diabetes mellitus published in 2007, show that genome-wide analyzes are able to identify genetic risk factors for common diseases. For preterm babies the identification of genetic risk factors would be of great value. These children are all cared for from birth in special neonatal intensive care units, so that children with special risks can be identified quickly and, if necessary, treated prophylactically. Prerequisite for the identification of genetic risk factors, however, are large patient cohorts, of which sufficiently large amounts of DNA are available. For more than 10 years, our group has been studying genetic risks for preterm babies born under 1,500 grams. Our DNA biobank currently includes samples of more than 10,000 preterm babies with a birth weight of <1,500 g and 7,000 term-born children (controls). For the majority of these children we also have maternal DNA samples. Our preterm DNA bank is, thus, one of the world's largest projects in this field.

Influence of preclinical and social factors

It is well-known that certain preclinical (e.g. birth due to an amniotic infection syndrome) and social factors (e.g. origin, maternal education) have a major impact on the long-term development of preterm babies. We expect that these factors - which are often very difficult to influence - have a greater impact on the long-term development of preterm infants than the genetic factors described above and have a similar impact as the center-specific therapies described below. So far there are no studies investigating the combined effects of genetic, social and clinical factors.

Center-specific therapies

With regard to the frequency of the above-mentioned endpoints, there are great differences between individual neonatal departments. Special emphasis was therefore placed on analyzing these differences and providing feedback to the participating study centers. We expect center-specific factors to have approximately the same impact on the short- and long-term development of preterm infants as preclinical and social factors. They, however, can be much better influenced (see below).

Benchmarking

All clinics participating in the study receive center reports after each monitoring, comparing their own center's clinical data with data from all other centers and data from the most successful centers (in relation to the short- and long-term outcomes mentioned above). Since we also collect a variety of treatment data, centers with unfavorable outcomes can quickly find out about differences in therapy compared to centers with good outcomes (always based on the respective outcome). We will also give the centers the ability to perform specific searches for their own records compared to all other records. We know from ongoing projects that the center reports we have prepared by many hospitals are already being used as quality indicators for their daily work.

Clinical studies

Furthermore, the goal of the preterm neonatal network described here is to promote the planning and conduct of drug studies in preterm babies. In recent decades, hardly any pharmaceutically sponsored clinical drug trials have been performed on children, particularly in preterm babies. As a result, the majority of the drugs used in neonatal intensive care units are not subject to formal drug approval. This problem has been recognized by the legislature and led to changes in pharmaceutical legislation specifically promoting studies in children and newborns. With our project, we want to support this process from a clinical perspective and provide a logistical framework for the pharmaceutical industry, and especially for scientists interested in clinical trials, to facilitate the conduct of drug studies in preterm babies. The network itself will not act as a sponsor or clinical coordination center.

The close cooperation of the centers within the framework of our existing project has already led to the establishment of first additional studies. As examples, two prospective randomized drug studies on the efficacy of spontaneously administered surfactant in preterm babies should be mentioned. In one study on the avoidance of artificial ventilation in preterm babies running since September 2007 (Avoid mechanical ventilation, EudraCT 2006-006912-31, ISRCTN 05025922, sponsored by Chiesi Pharmaceuticals, study leaders Profs. Göpel & Herting) already 50 preterm babies have been enrolled. Another clinical study to avoid chronic lung damage due to surfactant administration without intubation (funded by the German Research Foundation (DFG) and the Federal Ministry for Education and Science (BMBF), study leaders PD Dr. Kribs & Prof. Roth) will start this year.

5. Study design

5.1. Study type

The GNN is a prospective cohort study in which approximately 25% of all preterm babies born in Germany with a birth weight below 1,500 grams are included and followed up over a period of 6 years with respect to their physical and neurological development.

Inclusion criteria:

- Birth <= 36 + 6 weeks gestation
- Birth weight <1,500 g

Exclusion criterion:

- Transfer of the infant from another hospital.

5.2. Endpoints

Data on each individual patient are collected at 3 times:

1. During the immediate postnatal stay in the clinic.
2. During an outpatient re-appointment, at the age of 2 years.
3. During an outpatient re-appointment, at the age of 6 years.

The following relevant endpoints are analyzed:

During the primary stay in hospital:

- Death
- Blood culture-proven sepsis
- Cerebral hemorrhage (grading I-III, additional parenchyma involvement)
- Bronchopulmonary dysplasia (oxygen demand at 36 + 0 SSW according to NIH consensus definitions)
- Surgery because of
  - Persistent ductus arteriosus
  - Necrotizing enterocolitis
  - Hydrocephalus (shunt placement)
  - Retinopathy (laser-/ cryotherapy)
- Pahological hearing score
- Body weight, body length and head circumference under the 10th percentile (at admission and discharge)

During examination at the age of 2:

- Death

- Body weight, body length and head circumference below the 10th percentiles
- Bayley Scales for Infant Development II (English version) MIDII or PIDII <70

During the examination at the age of 6:

- Death
- Body weight, body length and head circumference below the 10th percentiles
- Kaufmann Assessment Battery for Children (K-ABC, German Version) score <70
- Strengths and difficulties questionnaire (module of the KIGGS Survey)
- Examination of motor performance, visual acuity test, examination of the stereo eye, blood pressure measurement (modules of the KIGGS Surveys)

Instruments of endpoint assessment:

The clinical endpoints during inpatient treatment are documented by the questionnaires used in our ongoing study. The Bayley Scales test the cognitive and motor development of children aged 1-42 months. With the Kaufmann ABC intelligence and acquired skills of children aged 2-12 years are tested. The "Strengths and Problems Questionnaire" is a parent questionnaire on the behavior of children and adolescents that was previously used in the KIGGS study. The other modules of the KIGGS survey were also standardized on a sample cohort of several thousand children. It consists of 10 tasks that test for coordination, strength and flexibility, a simple examination of stereo vision, a visual acuity test, and a blood pressure measurement. All clinical items formulated as endpoints are clearly defined and were previously used as endpoints in international studies. The tests and questionnaires are also standardized and have been validated on large groups of healthy children. They, too, are used as standards in international studies (with the exception of individual modules of the KIGGS survey) and also comply with the recommendations of the German specialist societies for the follow-up examination of preterm babies.

5.3. Collection of data with potential impact on endpoints

In addition to these endpoint assessment tools the study collects a wealth of data that could potentially affect the endpoints.

During the inpatient stay:

- Preclinical data (documentation sheet 1),
- All treatments during the inpatient stay (documentation sheet 2 & 3)
- KIGGS parent questionnaire (questions about social environment, nutrition etc.)

At the re-examination with 2 and 6 years

- Medical questionnaire about diseases

- KIGGS parent questionnaire (questions on social environment, nutrition, etc.)

At birth and at the age of 2 and 6 years, the questionnaires standardized in a large group of children of the KIGGS study are used (details at http://www.kiggs.de). The documentation of the data of the inpatient stay and at the time of neurodevelopmental follow-up at the age of 2 are provided by the study centers. The survey data on the environment (KIGGS parent questionnaires) and the examination at the age of 6 years is carried out by staff of the study center.

5.4. Proposed sample size

Since we expect the lowest effect sizes for genetic risk factors, the cohort size calculation for our study is based on the numbers required for the identification of genetic risk factors. Genome-wide studies in adults indicate that 500-2,000 affected patients are needed to identify genetic risk factors for a single disease. Since the incidence of the outcomes described above is between 3 and 20% in preterm babies with a birth weight below 1,500 grams, a number of 20,000 prospective children enrolled in the study is necessary to identify genetic risk factors. Thus, the final aim of the present study is to enroll and follow up 20,000 preterm babies with a birth weight below 1,500 grams over a 12-year period. For such a high case number, the permanent participation of about 50 large neonatal departments in the study is planned. We are aware that the participation in this study for the participating centers means additional expenses. From the very beginning, we therefore paid attention to making the study as easy as possible to implement in everyday clinical practice. There are also the following benefits for participating centers:

- Regular reports allow to compare therapy and outcome data with other centers.
- Participation in the study fulfills the legal requirements given by the Federal Joint Committee on "Measures to ensure the quality of care for preterm babies and newborns" of Sept. 20, 2005.
- For the tracking of patients, we will set up an address database maintained by the study center. The participating centers will be informed about address changes of their preterm babies. This greatly simplifies the communication for organizing the obligatory examination of preterm babies at 2 years.
- In order to relieve the participating centers and to enable a more objective assessment of the long-term course (reduce inter-observer variability) , the 6-year study is carried out by one study team of the study center in Lübeck. As with the short-term outcome data, the centers receive feedback on their patients as compared to those of all other centers.
- For the documentation of the data up to the 2-year examination, a lump-sum remuneration is paid per completed patient to the participating centers.

5.4. Study conduct

5.4.1 Inclusion of study centers

Due to the high number of cases required, the study was planned as a multi-center nationwide study. Since the study center invests considerable resources into the coordination of the individual centers, our goal is to get as large neonatological departments as possible to participate in this study, as this will not only increase the number of necessary patients more quickly, but also the resources of the patients study center can be used more targeted. Neonatological departments with less than 40 preterm infants <1,500 g per year should be included in this study only in exceptional cases. The admission of study centers is carried out by the study director.

5.5.1. Training of employees at the centers

Before the inclusion of a patient at the respective center, a staff training in the participating center is carried out by a doctor of the study center covering the objectives and course of the study. As part of this training the participating center is provided with 50 pre-packaged envelopes for patient inclusion and 50 containers for the asservation of umbilical cord samples.

5.5.2. Inclusion of patients and documentation of clinical data

In addition to a detailed information form, a consent form and a questionnaire on the preterm basic clinical data, the study center's patient inclusion set contains 4 sterile smear tubes for collecting 2 oral mucosal swabs each from the preterm infant and the baby's mother. The basic clinical data questionnaire (Documentation Sheet 1 of 3) will be sent to our laboratory along with the oral mucosal swabs. Furthermore, a short piece of umbilical cord (collected immediately after birth) is frozen at minus 20 °C from each preterm infant participating in the study. Once the child has been discharged, data collection forms 2 and 3 (one of which is identical to the neonatal questionnaire) are completed and sent to the study center or faxed. The flow chart on the following page summarizes the inclusion of patients.

Flowchart for participating study centers

***Inclusion criteria*: Preterm infants <= 36 + 6 weeks gestational weight <1,500 g**

The child was not transferred from another children's clinic

Inclusion criteria met

Hand over the information and consent form to the parents

Consent given

2 oral mucosal smears are taken from mother and preterm infants

Completion of the data collection form (1/3)

Filing the consent form in the study folder

Shipping of oral smears and data collection sheet 1/3

In data collection sheets 2/3 and 3/3, at first only the name of the child and the date of birth are entered. The sheets are included in the child's file and sent to the study center after he/she has been discharged.

In addition to the oral mucosal smears, a short piece of umbilical cord of each infant (obtained, for example, when shortening the umbilical cord as part of primary care) is frozen at minus 20 °C as soon as possible after birth.

The stored umbilical cord samples are collected by a doctor of the study center as part of the monitoring, so they do not have to be sent. At six-monthly intervals, the data collected will be reviewed by the coordinating study center in Lübeck and all participating centers will receive a written report commenting on both the clinical procedures and outcome data compared to the other centers participating in the study.

The detailed collection of drugs administered and procedures performed (data collection questionnaire 2/3) allows us to study the interplay between exogenous factors and specific genetic predispositions. A major benefit for the participating centers is a regular report of their performance which is provided by the coordinating study center and can provide important suggestions for the standard management of individual centers and the resulting clinical outcome. However, data comparability requires regular monitoring of data quality. Patient inclusion and documentation of the clinical data is the task of the respective centers; the data is entered into a database (double-checked) at the study center.

5.5.3. Data bases and data protection

In order to be able to follow the preterm infants over a period of 6 years, a comprehensive tracking system is established by the study management. Two databases are created for the entire study. The first database contains the personal data of preterm babies (date of birth, name, address, clinic, etc.) including the number of completed and certified documentation sheets. To avoid having to question parents of deceased children, even the possible death of a child is documented in this database. The clinical data of the patients are stored in a second, pseudonymized database, which is linked to the personal database only by a number. A feedback from data from the pseudonymized database to the parents, the supervising clinic or other persons is excluded, since otherwise the anonymity of the study participants would not be maintained. Conversely, however, the data in the pseudonymous database can later be improved by adding further data obtained in the course of follow-up examinations.
For the maintenance of the address database parents of all preterm babies participating in the study will be contacted by the study center with a greeting card for the child's birthday and informed about the course of the study. In the case of undeliverable cover letters, an address is determined via the supervising clinic or the communal registration offices.

5.5.4. Documentation of the follow-up examinations

Due to the commitment of the Joint Federal Committee for the Care of Preterm Infants, all preterm babies aged 18-24 months will be re-examined by the participating centers. The recommended method of investigation is the Bayley II scores, which is applied by the centers. Furthermore, the children are measured and weighed and there is a standardized collection of disease, surgery and medication data (KiGGS questionnaire; *The German Health Interview and Examination Survey for Children and Adolescents (KiGGS): sample desing, response and non-response analysis*. Kamtsiuris P, Lange M, Schaffrath RA. Bundesgesundheitsblatt Gesundheitsforschung Gesundheitsschutz. 2007 May-Jun; 50(5-6):547-56. German). Finally, the parents are also interviewed by means of a standardized questionnaire (KiGGS parent questionnaire). At the age of 5-6 years there is a second follow-up. This follow-up is done by a study doctor of the coordinating study site in Lübeck. The developmental neurological test methods planned for this time are the merchant ABC and the "Strengths and difficulties questionnaire". Children are also measured and weighed and relevant data on diseases, surgeries and medication (KIGGS questionnaires) are again collected. The parents are

interviewed by means of a standardized parent questionnaire (KiGGS parent questionnaire). Detailed documentation sheets for this examination are not yet stored in the current version of the study protocol as they are to correspond to the current recommendations of the specialist societies at the time of examination (i.e. in 5-6 years).

5.5.5. Monitoring and quality control

Since the study described here is intended to obtain data for the conduct of prospective clinical trials, the study design follows the recommendations for "*Good Clinical Practic* ". This requires appropriate monitoring of the participating centers.

5.5.5.1. Monitoring procedures prior to inclusion of a study center

Prior to the inclusion of a center in the study, hospitals that are interested in participating will be interviewed. This serves to ensure a sufficient center size. With regard to a clinical drug study, important center properties (e.g. possibility of carrying out ultrasound examinations, X-rays, laboratory examinations) are also requested.

5.5.5.2. Monitoring during the study

All study centers are visited by a doctor from the study center every six months. The monitoring is verifying the completeness of the clinical data on the basis of the source data (patient records), the inclusion rate of patients who fulfill the inclusion criteria of the study based on the source data (usually ward book) as well as a numerical collection of the parameters death, delivery mode, ventilation, laser- or cryocoagulation ROP, NEC or ileum perforation requiring surgery, VP shunt, gestational age, birth weight, sepsis with positive blood culture, surgery of a persistent ductus arteriosus, IVH grade, PVL, BPD and pathological hearing test for all preterm infants that met the inclusion criteria, but were not enrolled in the study. The documentation of these data takes place in the form of a counting list and serves to make the individual centers as comparable as possible. For each visit, a written report is provided by the respective monitor, which is filed in the study folder of the participating center and in the study center. The monitoring of the follow-up data at the age of 2 is carried out analogously by employees of the study center. After monitoring the data, all complete data sets and the associated DNA samples are pseudonymized.

5.5.6. Sample processing and laboratory tests

For each patient participating in the study 5 DNA samples will be taken: 2 oral mucosa smears each from mother and child are sent to the study center as part of the submission of the first documentation sheet, and a short piece of frozen umbilical cord is stored at the participating centers and collected by the doctor of the study center during the monitoring visit. Since the quality and quantity of umbilical cord DNA is much higher than that of oral mucosal DNA, only one maternal DNA sample is initially isolated from the oral mucosal specimens. The remaining oral mucosa smears remain frozen. After the monitoring visits DNA is isolated from the deep-frozen umbilical cord samples for each preterm baby

and divided into 3 portions (1 sample for genome-wide investigations, 1 sample for the selective examination of interesting polymorphisms, 1 sample as reserve and control). Should for individual patients no umbilical cord be preserved, the DNA will be isolated from the buccal swab and frozen as a sample for examination of interesting polymorphisms. Genome-wide studies only make sense from a very high number of cases of about 500 preterm babies affected by a specific disease (e.g. sepsis), so that we are planning first genome-wide examinations only from the second year of study on. Prior to these genome-wide studies, we will investigate candidate gene polymorphisms that were predictive of preterm disease in adult genome-wide studies (e.g. risk genes for diabetes mellitus and their impact on the incidence of postnatal insulin therapy in preterm babies) or in premenstrual candidate gene studies.

5.7. Case numbers and biometric concept

Genetic and risk factors

The following endpoints are planned for the first genome-wide investigations:

- prematurity
- Late-onset sepsis with positive blood culture
- Cerebral hemorrhage of the preterm baby
- Growth retardation (birth weight below the 10th percentile)
- Mental and psychomotor retardation at the age of 2 years

For all these outcomes, we expect a frequency of at least 20% on the basis of preliminary examinations, so that we are very likely to be able to identify valid genetic risk factors for these diseases already after 6 years. Sufficiently large number of cases for rare diseases of prematurity (e.g. necrotizing enterocolitis, ductus arteriosus requiring surgery, retinopathy requiring laser- or cryotherapy, a pathological hearing test in the neonatal period etc.) are expected after about 8-10 years. Genetic risk factors for mental retardation or disability, which can reliably be determined only during the follow-up at the age of 6 years, we expect after 9-12 years project duration. As a control group, we will select a group of preterm infants with genome-wide examinations who did not experience any complications during the clinical course, and who show normal physical and mental development at the age of 2, despite having extremely low birth weight (<1,000 g) and low gestational age (<29 weeks). From all preterm infants who meet these criteria, a control group of 1,000 children is randomly chosen for genome-wide examinations. Background of the creation of such a group of "resilient preterm infants" (i.e. no disease despite extremely high disease risk) is the expectation that in this group risk alleles are rare and protective alleles for diseases of preterm infants occur more frequently than in the normal population, whereas in the group of preterm infants with certain diseases (e.g. sepsis) we expect an accumulation of risk alleles and a rarer occurrence of protective alleles compared to the normal population. Overall, this results in a higher difference of allele frequency, so that predictive

polymorphisms can be detected more easily. Comparisons with published genome-wide health records are also planned.

6. Ethics

The study protocol is advised by all institutional review boards of those sites participating in the study. The study center is the Department of Pediatric and Adolescent Medicine of the University of Lübeck. The vote of the ethics committee of the medical faculty of the University of Lübeck is attached.

7. Costs

All our investigations and data collections are free of charge for the participating study centers as well as for the patients participating in the study.

8. Selected publications of our research group

Ahrens P, Kattner E, Köhler B, Härtel Ch, Seidenberg J, Segerer H, Möller J, Göpel W. Muta- tions of Genes Involved in the Innate Immune System as Predictors of Sepsis in Very Low Birth Weight Infants. Ped Res 2004, 55: 652-56 Commentary: Genetic Screening for Susceptibility to Infection in the NICU Setting. Ped Res 2004, 55: 546-48

Göpel W, Kim D, Gortner L. Prothrombotic mutations as a risk factor for preterm birth. Lancet 1999; 353:1411-2

Göpel W, Gortner L, Kohlmann T, Schultz C, Möller J.Low prevalence of large intraventricular haemorrhage in VLBW-infants carrying the factor-V-Leiden or prothrombin-G20210A muatation Acta paediatrica 2001; 90: 1021-24

Göpel W, Ludwig M, Junge AK, Kohlmann T, Diedrich K, Möller J. Positive selection pressure for the factor V Leiden mutation due to improved fetal implantation. Lancet 2001; 358: 1238-39

Göpel W, Kattner E, Seidenberg J, Kohlmann T, Segerer H, Möller J. The effect of the Val34Leu polymorphism in the factor XIII gene in infants with a birth weight below 1500 g
J Pediatr 2002; 140:688-92

Härtel Ch, Finas D, Ahrens P, Kattner E, Schaible Th, Müller D, Segerer H, Albrecht K, Möller J, Diedrich K, Göpel W. Polymorphisms of genes involved in innate immunity – association to preterm delivery. Mol Hum Reprod 2004; 10:911-5.

Härtel C, von Otte S, Koch J, Ahrens P, Kattner E, Segerer H, Möller J, Diedrich K, Göpel W. Polymorphisms of haemostasis genes and preterm delivery. Thromb Haemost 2005; 94;88-92

Göpel W, Härtel C, Ahrens P, König I, Kattner E, Kuhls E, Küster H, Möller J, Roth B, Segerer H, Wieg C, Herting E. Interleukin-6-174-genotype, sepsis and cerebral injury in very low birth weight infants. Genes Immun 2006; 7:65-8

Härtel C, König I, Köster S, Kattner E, Kuhls E, Küster H, Möller J, Müller D, Roth B, Segerer H, Wieg C, Herting E, Göpel W. Haemostasis genes and primary outcome of very-low-birth-weight- infants. Pediatrics 2006; 118:683-9

Göpel W. A spitting image of the lungs (Comment). Pediatrics 2006, 117:2285-6.

Hartel C, Puzik A, Göpel W, Temming P, Bucsky P, Schultz C. Immunomodulatory effect of vitamin C on intracytoplasmic cytokine production in neonatal cord blood cells. Neonatology 2007; 91:54-60.

Härtel C, Schultz C, Herting E, Göpel W. Genetic association studies in VLBW infants exemplifying susceptibility to sepsis – recent findings and implications for future research. Acta Paediatr 2007 96:158-65.

Härtel C, Osthues I, Rupp J, Haase B, Röder K, Göpel W, Herting E, Schultz C. Characterization of the host inflammatory response to Staphylococcus epidermidis in neonatal whole blood. Arch Dis Child Fetal Neonatal Ed. 2008; (Epub ahead of print).

Spiegler J, Kattner E, Vochem V, Küster H, Möller J, Müller D, Kribs A, Segerer H, Wieg C, Nikischin W, v.d.Wense A, Gebauer C, Herting E, Göpel W. Very low birth weight infants as a model to study genetic influences on neonatal weight gain. J Pediatr Gastr Nutr 2008, 46:113-6.

Deutsches Frühgeborenen-Netzwerk German Neonatal Network

Erklärung zur Teilnahme an der Studie
Declaration of Commitment of Participating Centers

Klinik:____
(Institution)

Name des teilnehmenden Arztes:_______________________________
(Name of investigator)

Einschlusskriterien (inclusion criteria):
Frühgeborene mit einem (preterm infants with)

- Geburtsgewicht < 1500 Gramm (birth weight < 1500 grams)
- Gestationalter <=36+6 Schwangerschaftswochen (gestational age <= 36+6 weeks)

Ausschlusskriterium (exclusion criteria):

- Verlegung des Kindes aus einer anderen Kinderklinik (transfer from another hospital with Paediatric Department)

Anzahl der Patienten pro Jahr, die obige Kriterien erfüllen und in die Studie eingeschlossen werden können:

(Number of patients expected to be recruited for the study (per year) under the above-mentioned criteria):

N=____________**Patienten/Jahr** (patients/year)

Ich kenne das Protokoll für die oben genannte Studie. Mir ist bekannt, dass das Protokoll die Sammlung von DNA-Proben, die Dokumentation des klinischen Verlaufs und die Dokumentation der Nachuntersuchung im Alter von 2 Jahren (entsprechend den Empfehlungen des Gemeinsamen Bundesausschusses) vorsieht. Ich bin über die Höhe der vorgesehenen Dokumentationspauschale für jeden vollständig dokumentierten Patienten (50€) informiert.
(I declare to be informed about the study protocol. I am aware that this includes the collection of DNA samples, the documentation of clinical data and of the 2-year follow-up examination – according to the recommendations of the Joint Federal Commission). I have been informed about the documentation fee for each completely documented patient of 50 €.

Es bestehen keine wirtschaftlichen oder andere konfligierenden Interessen der teilnehmenden Klinik oder Person im Zusammenhang mit der Studie.
(No conflicts of interests with regard to the study are declared.)

Unterschrift (Name und Anschrift oder Stempel)

(Signature) (Name and address)

German Neonatal Network, GNN

Information sheet for parents

Dear parents,

Your child was born with a birth weight below 1,500 grams and well before the expected date of birth. We know that most preterm babies, even those born with such low birth weight, can be released from the hospital after a period of time that seems very long to their parents. Unfortunately, there is little data on the long-term development of very small preterm infants in Germany. In particular, it is not known what influence the preterm birth itself, but also the therapy in the hospital, the genetic predisposition of the individual child and subsequent environmental factors have on the long-term physical and mental development of preterm babies.

What is the purpose of this study?

The most important goal of our study is to find factors that have a positive influence on the long-term development of preterm babies. Only if one knows which form of therapy, which genetic peculiarity and which environmental factors have a favorable influence on the long-term development of the preterm infants, one can use these therapies and environmental factors purposefully and develop perhaps also medicaments, which mimic particularly favorable genetic characteristics. Conversely, one can of course avoid unfavorable factors. Our study thus serves the future better treatment of very small preterm babies. The data registered in the study will only be collected and used for this purpose.

How is the study conducted?

Of all preterm babies participating in our study, data are collected at three time points: during inpatient stay after birth, at the age of 2 and at the age of 6. During your inpatient stay, your child's essential clinical data (e.g. birth and discharge weight) will be registered by the physicians. In order to be able to investigate possible genetic influences on the development of preterm babies, we need 2 oral mucosal smears each of mother and child and a short remainder of the child's umbilical cord. The smears are made with a sterile cotton swab and are not a burden even for very young children. The umbilical cord sting is taken when the umbilical cord is shortened at the first treatment of the child and would otherwise be discarded. No other research is done on the material used and privacy is respected. At no time will blood samples be taken for the study. At the age of 2 years, your child's motor development will be tested. This study is recommended for all preterm babies with a birth weight below 1500 grams; it is not an investigation that is done extra for our study. Finally, at the age of 6, an examination of mental and physical development (including an eye test) is planned. The follow-up examinations at the age of 2 and 6 take place in the clinic where your child was cared for right from the beginning, so you do not have long journeys. The result of the follow-up examination will be discussed with you immediately. We will also send you a parent questionnaire at all three times (*i.e.* while your child is still in the hospital, at the age of 2 and at the age of 6). This parent questionnaire was already answered by more than 17,000 parents whose children are not preterm infants. Thus, this study will enable us to find out whether preterm babies differ in terms of their diet, the frequency of visits to a doctor etc. from children who are not pretermly born. So that you can complete these questionnaires in peace, we will send them as pseudonymized (*i.e.* only with a number) sheets directly to your home address.

Who participates in the study and how long will the data be stored?

Our study is the largest study to date in Germany on the long-term development of preterm babies. 50 neonatal departments throughout Germany participate in our study. We plan to include approximately 20,000 preterm infants in our study over a 10-year period and expect to store the anonymized data for at least 18 years, when the last children included in the study will be re-examined.

How are the data protected?

Protecting the personal data of the preterm babies participating in the study is particularly important to us. For this reason, we will store all data in a pseudonymised way (i.e. all personal data such as name and address of the participating children will be replaced by a number). Therefore, we cannot tell you any individual results of genetic testing. In order to be able to invite you in time for the follow-up examinations, we would like to ask you for permission to save your address. We will only match your address with the pediatric clinic or the registration office and will not pass it on to any third party. Both the address file and the clinical data files are protected by special passwords and programs so that nobody has access to your data. It is particularly important to us to inform you about the progress of our study. We would therefore like to ask you for your consent that we send you a short information letter once a year (for the birthday of your child). After the examination at the age of 6 years, all data will be completely anonymized, i.e. your personal data will be completely deleted. Participation in this study is, of course, voluntary, and if you do not participate in the study, you or your child will not experience any disadvantages. You can, of course also withdraw your participation in the study. In this case, all data (personal and clinical) will be deleted and all samples will be destroyed. However, this is only possible until the examination at the age of 6 years, because at this time all personal data will be deleted anyway. If you would like to withdraw your participation in the study please write to the "German Neonatal Network, Department of Pediatrics of the University of Lübeck, 23538 Lübeck".

How are the results of the study published?

Basically, no data on individual clinics or individual children are published or passed on to third parties. All clinics participating in the study receive regular reports on their preterm babies. In order to inform as many interested physicians as possible, but also other interested parties, about the results of our work, we will publish the overall results of the study in scientific journals and on the internet.

How is the study funded?

The study is funded exclusively by public funding from the Federal Ministry of Education and Research. It is possible that in the future there will be commercial exploitation of the research results to which the researchers and universities participating in the study will contribute. In this case, you give up with your consent to any financial claims that could possibly result from the marketing of the test results.

Who is responsible for the content and conduct of the study?

Many doctors have contributed to the content of this study. The study content was discussed with representatives of the professional societies for neonatology, perinatology and social pediatrics and experts for the statistical analysis of data. In addition, we interviewed many parents of preterm babies about our study project and coordinated the study with the Federal Association "The Preterm Infant", the largest association of preterm birth parents in Germany. For the implementation of the study, the study director Prof. Dr. med.

Wolfgang Göpel (Department of Pediatrics, University of Lübeck, 23538 Lübeck). If damage occurs in the course of the study, which was inflicted on the study participants by the guilty behavior of an employee of the University Medical Center Schleswig-Holstein (UKSH), the UKSH is liable.

We hope to be able to help little babies even better in the future through this study. If you have any further questions about the study, please contact the doctor or nurse treating your child or one of the undersigned doctors of the study administration.

Doctor of the participating center

Prof. Dr. med. med. W. Göpel
(Study Center University Children's Hospital Lübeck)

Study „*Deutsches Frühgeborenen-Netzwerk (German Neonatal Network, GNN)*"

Consent form

I hereby declare my / our consent to the participation of my / our child (s)

____ born on ________________________

in the study "German Preterm Neonatal Network (German Neonatal Network)"

I have received, read and understood the written patient information for the above study. I have been verbally and in written form informed about the purpose and the course of the study.

I was advised that my child's participation is voluntary and that I have the right to terminate it at any time without notice for any reason without any disadvantages for me or my child.

I am informed and agree that the clinical progress data of my child and my address are forwarded to the study center (Children's Hospital of the University of Lübeck) and that my address data (e.g. in the case of a change of residence) are compared with the clinic treating my child or the population register.

I have been informed that the samples taken are only used anonymously for the investigation of influencing factors for preterm birth and preterm birth and that a report of the genetic data cannot be sent to me or to the doctors treating my child.

I / we had the opportunity to ask all my / our questions, these were answered satisfactorily and completely.

Place, date Signature of guardian (s)

Place, Date Informing Physician (Name) Informing physician (Signature)

Please fill in data sheet and send it, together with two buccal swabs of mother and child, to the study center (Department of Pediatric and Adolescent Medicine, Prof. Dr. W. Göpel, prematurity study, 23538 Lübeck).

Children's Hospital NN

Study " *German Preterm Neonatal Network (GNN)*"

Documentation sheet on study inclusion (1/3)

Initials first name/family name____/_____
Date of birth:
Has umbilical cord been frozen?
☐ yes ☐ no

1. Sex

☐ female
☐ male

2. Multiples

☐ no ☐ yes

if yes:
Twin No.____________ of_________ In case of twins:
☐ monozygotes ☐ dizygotes ☐ unknown

3. Birth

Mode:
☐ spontaneous ☐ sectio
APGAR 5 and 10 minutes:____/_____ Umbilical artery-ph:_

4. Birth weight and gestational age

______________gramms
calculates birth date: ....________
or
gestational age:__________weeks +_______days

5. CRIB score

Maximal BE during the first 12h: __________________
Minimal oxygen during the first 12h:________________
Maximal oxygen during the first 12h:________________
inherited malformations:
☐ none ☐ not life-threatening
☐ acutely life-threatening (ISTA, CHARGE, Fallot, hydrops, omphalozele, OI, VACTERL, urea cycle defect, lung hypoplasia, prune belly, polysystic kidney degeneration)

6. „Inborn" (birth at the clinic neonatal intensive care)

☐ no ☐ yes

7. Antenatal lung maturation treatment

☐ no ☐ yes
if yes**:**
☐ Betamethasone
☐ Dexamethasone
☐ Other medication: _________________________

8. Antenatal tocolysis treatment

☐ no ☐ yes
If yes:
☐ Magnesium
☐ Fenoterol (Partusisten®)
☐ Atosiban (Tractotile®)
☐ Other medication: _________________________

9. Antenatal antibiotic treatment

☐ no ☐ yes

If yes:
☐ Cephalosporine
☐ Metronidazol (Clont®)
☐ Ampicillin or other penicilline
☐ Imipenem/Meropenem
☐ Other:__________________________________

10. Reason for preterm birth

☐ Preterm labour
☐ Amniotic infection syndrome
☐ EPH gestosis
☐ HELLP
☐ pathological CTG
☐ Growth retardation and pathological Dopplers
☐ Placental abruption
☐ Others: _________________________________

11. Origin of the mother

☐ Germany
☐ Other Europe, including Russia
☐ Near East, including Turkey and North Africa
☐ Asia
☐ Africa
☐ Others: _________________________________

12. Mother's age

____________years

13. Previous births

☐ no ☐ yes
If yes:
Preterm birth(s) (before week 37)?
☐ no ☐ yes

This confirms the accuracy of the information. Signature (documenting physician)

Please fill in data sheet after the child's release and send it to the study center (Department of Pediatric and Adolescent Medicine, Prof. Dr. W. Göpel, prematurity study, 23538 Lübeck).

Childdrens clinic NN

German Neonatal Network (GNN) Study

Documentation sheet / examination (2/3)

Name of the child

7. Inhalative medication

Date of birth

1. Prophylactic medication:

Vitamin K : ☐ oral ☐ i.m. ☐ i.v. ☐ s.c. others:

☐ Teicoplanin/Vancomycin for gram-positive sepsis prophylaxis

☐ systemic Fluconazol for fungal infection prophylaxis

☐ Ibuprofen ☐ Indomethacin for persistent Ductus arteriosus prophylaxis

☐ Erythropoeitin for anemia prophylaxis

☐ ________________________for prophylaxis of

____

2. Antibiotic therapy

☐ no ☐ yes

If yes:

☐ Penicillin ☐ Ampicillin/Amoxicillin

☐ Cefotaxim ☐ Imipenem

☐ Gentamicin ☐ Tobramycin

☐ Erythromycin ☐ Vancomycin

☐ Teicoplanin ☐ Metronidazol

☐ ____________________________________

3. Catecholamine therapy

☐ no ☐ yes

If yes:

☐ Dopamine ☐ Dobutamine

☐ Noradrenaline ☐ Adrenaline

☐ ____________________________________

4. Analgetics und sedativa

☐ no ☐ yes

If yes:

☐ Midazolam (Dormicum®) ☐ Diazepam

☐ Morphin ☐ Fentanyl

☐ ____________________________________

5. Surfactant

☐ no ☐ yes If yes, how often:

☐ 1 x ☐ 2 x ☐ 3 x ☐_x

Surfactant without intubation ☐ yes ☐ no

6. Diuretics

☐ no ☐ yes

If yes:

☐ Furosemid (Lasix®)

☐ Hydrochlorothiazid

☐ Spironolactone (Aldactone®)

☐ ____________________________________

☐ no ☐ yes

If yes:

☐ Salbutamol (Sultanol®)

☐ Ipratropiumbromid (Atrovent®)

☐ Budenosid (z. B. Pulmicort®)

☐ ____________________________________

8. Transfusions

Blood: ☐ none ☐ 1 ☐ 2 ☐ 3 ☐____(n)

FFP: ☐ none ☐__(n) **TK**: ☐ none ☐____________(n)

9. Breath analeptic medications

☐ no ☐ yes If yes:

☐ Theophyllin ☐ Coffein

☐ Doxapram ☐ ________________

10. Other medications

☐ Insulin ☐ Vitamins (except Vit. D)

☐ Iron ☐ ______________________

☐ ____________________________________

☐ ____________________________________

11. Were meds stopped for any reason?

☐ no ☐ yes

If yes________________ reason_________________

12. Central-venous catheter

☐ no ☐ yes

If yes:

☐ Naval venous catheter

☐ Polyurethane catheter

☐ Silicone catheter

Removal due to complications?? If yes, please mark catheter type:

☐ Dislocation (________________________)

☐ Catheter infection (________________________)

☐ Thrombosis (________________________)

13. Arterial catheter:

☐ no ☐ yes

If yes: ☐ Navel ☐ peripheral artery catheter *Did catheterization lead to necroses?*

☐ no ☐ yes

14. Mechanical ventilation:

☐ no ☐ yes

If yes

- conventional lung support ☐ nein ☐ ja
- high-frequency ventilation ☐ nein ☐ ja
- mononasal CPAP ☐ nein ☐ ja
- binasal CPAP ☐ nein ☐ ja

Pneumothorax ☐ nein ☐ ja falls If Pneumothorax please list ventilation/CPAP type at the time of the pneu:____________________

15. Feeding support

Start on day ______

Completely oral from day _____ on.

This confirms the accuracy of the information. Signature (documenting physician)

Please fill in data sheet after the child's release and send it to the study center (Department of Pediatric and Adolescent Medicine, Prof. Dr. W. Göpel, prematurity study, 23538 Lübeck).

Children clinic NN

German Neonatal Network (GNN) Study

Documentation sheet / examination (3/3)

This documentation sheet contains points 5/6, 14-16 und 19-24 of the German Neonatal Assessment Sheet which may be used alternatively. In the latter case please make sure that all the relevant items are answered, especially any surgeries (see 9.)

Name of the child

Date of birth

1. Gestational age:

Calculated birth date: ...._________
or
Gestational age:__________weeks +________days

2. Admission

Body temperature at admission:_____________° C
Weight at admission:___________________gramms

Length:
Head circumf.:

3. Oxygen support

☐ no ☐ yes If yes:

Start: ______.._____ ________

End:__________________________ _____ ___________

4. Mechanical ventilation (non-CPAP)

☐ no ☐ yes
If yes, start: ....________
End: ....__________
End of all lung support: ....______

5. Sepsis

☐ no ☐ yes

If yes, start: ...._________
Pathogens in blood, CSF or urine:

none (00)	☐
Staphyl. aureus (31)	☐
Staphyl. epidermidis (32)	☐
Streptococc. group B (33)	☐
Strep. group D (Enterokokken) (34)	☐
Strep. other groups (35)	☐
Pneumococcous (36)	☐
E. coli (37)	☐
Klebsiella (38)	☐
Enterobacter (39)	☐
Serratia (40)	☐
Proteus (41)	☐
Pseudomonas aeruginosa (42)	☐
Listeria monocytogenes (43)	☐
Anaerobic coccous (44)	☐
Bacteroides spec. (45)	☐
Candida (46)	☐
Other pathogens (47)	☐

Length:
Head circumf.:

Typical clinical symptoms ☐ no ☐yes
Maximal CRP:______________________mg/dl
Maximal I/T ratio: _________________________
_____________________________________ If 2. Sepsis: pathogen cat.: ________________________
If 3. Sepsis: pathogen cat.: __________________

6. Head sonography

☐ no ☐ yes
If yes:
IVH: ☐ no ☐ yes If yes: max. stade

Grade 1: sub-ependymal	☐
Grade 2: < 50% ventricle lumen	☐
Grade 3: > 50% ventricle lumen	☐
Grade 4: intraparenchymal	☐

Periventricular leukomalacia ☐ no ☐ yes

7. Ophthalmological examination

☐ no ☐ yes
If yes, retinopathy: ☐ no ☐ yes If yes, max. stade:__________________________

8. Hearing screening

☐ no ☐ yes

If yes:☐ abnormal ☐ normal

9. Surgeries

Surgical PDA ligation ☐ no ☐ yes Laser or cryo ROP therapy ☐ no ☐ yes Abdominal NEC surgery ☐ no ☐ yes

Abdominal focal surgery intestinal perforation	☐ no	☐yes
VP shunts	☐ no	☐yes
inguinal herniotomy	☐ no	☐ yes
other surgeries	☐ no	☐ yes

____

10. Discharge:

Body weight:___________________________gramms
______________cm
______________cm

Stay ended on _________________ ______ ___________
Discharge to

Home	☐
Obstetrics	☐
Other clinic	☐
Death	☐

If death, cause:_____________________________________

11. other relevant diagnoses (ICD10):

____________ ___________
___________ ___________ ___________
___________ ___________ __________

This confirms the accuracy of the information. Signature (documenting physician)

Sheet 1

5. lung ripening treatment before birth:

Here, "yes" should be ticked even if only one dose of glucocorticoids was given to the mother, i.e. lung treatment does not have to be completed. The pulmonary treatment may also be several weeks earlier (also in this case tick "yes").

6. Tocolysis:

In contrast to pulmonary maturation treatment, only tocolyses that were used in the last 24 hours before birth are documented. However, it is not necessary that the tocolysis was given over 24 hours. A single dose, or the beginning of a continuous infusion with Mg or fenoterol is always documented as "yes". A tocolysis given days or weeks earlier is not documented. Any oral administration of magnesium is also not documented.

7. Antibiotics: As with tocolysis, only the antibiotics used in the last 24 hours before birth are documented, but only once.

8. Causes of preterm birth

Here several items may be ticked. Basically, all items should be ticked, which contributed to the preterm birth.
Example 1: Preterm birth in EPH gestosis, growth retardation and pathological Dopplers: Both EPH gestosis and growth retardation and pathological Doppler are documented here.
Example 2: Preterm birth due to EPH gestosis (maternal indication): even with simultaneous growth retardation, only EPH gestosis would be encoded here.

10. Pre-births: Here only births (defined as stillbirths above 500 g birth weight or live births) are defined as "yes". Abortions in early pregnancy are not counted as a previous birth.

Sheet 2:

Basically, the treatment of the individual patient should be documented as completely as possible by means of this sheet. In the foreground, however, are the therapies used in each center, *i.e.* drugs that were given in another clinic (in which the child was brought, for example, at short notice due to an operation) are not documented. Similarly, medications that the child receives in other departments (e.g. inhalation narcotics during surgery) are not documented. However, if an operation is performed in the neonatal intensive care unit and the child is looked after by the team during the operation, the given medication will be documented.
Basically, the one-time administration of a drug triggers the appropriate documentation (e.g. single administration of fentanyl for intubation = fentanyl "yes"). The item "complete oral nutrition" is defined as the day of life when the enteral amount of food is at least 150 ml / kg of body weight.

Sheet 3:

The definitions on this sheet follow the neonatal survey sheet. This is particularly important for item 5

(mechanical ventilation): CPAP times are coded here only in the case of previous mechanical ventilation (as the "final termination of each respiratory aid"). If a child was primarily provided with CPAP, it will only be documented on Sheet 2 (but without the duration of the CPAP application).

German Neonatal Network (GNN)

Study of the long-term development of preterm babies < 1,500 g

Parent Questionnaire
(re-examination at 6 years of age)

Question blocks:

1. Personal data (questions 1-16)
2. Health state of the child (questions 17-39)
3. Pain-related questions (questions 40-44)
4. Accidents (questions 45-56)
5. Development & nutrition (questions 57-59)
6. Childcare (questions 60-66)
7. **Well-being** (questions 67-89)
   - All items used for the antenatal GC study were from this block)

7.1 **Question 67: "How good do the following descriptions fit your child? Please only consider the child's behavior during the last 6 months.**
- All items used for the antenatal GC study were from this question)

Possible answers:
- "Does not fit." (counted as a "no" for the antenatal GC study)
- "Fits partly." (counted as a "yes" for the antenatal GC study)
- "Fits well." (counted as a "yes" for the antenatal GC study)

7.1.A: "Considerate"
7.1.B: **"Restless, hyperactive, cannot sit still" (counted as "hyperactivity" in the antenatal GC study)**
7.1.C: "Often complains about headache, stomachache or nausea"
7.1.D: "Likes to share with other children (sweets, toys, crayons etc.)"
7.1.E: **"Often suffers from tantrums; is quick-tempered" (counted as "quick tempered" in the antenatal GC study)**
7.1.F: "Loner; plays alone most of the time"
7.1.G: "Is generally compliant; does what adults ask for (most of the time)"
7.1.H: "Worries a lot; appears to be in a bad mood frequently"
7.1.I: "Is helpful when others are injured, sick or depressed"
7.1.J: **"Fidgety and nervous all the time" (counted as "nervous" in the antenatal GC study)**
7.1.K: "Has at least one good friend"
7.1.L: "Often fights with other children or bullies them"
7.1.M: "Is frequently depressed; cries often"
7.1.N: "Is popular with other children"
7.1.O: "Easily distractible, unconcentrated"
7.1.P: **"Nervous and clinging in novel situations; low self-confidence" (counted as "low self-confidence" in the antenatal GC study)**
7.1.Q: "Kind with younger kids"
7.1.R: "Often lies or cheats"
7.1.S: "Is often teased or bullied by other kids"
7.1.T: "Helps others voluntarily, e.g. parents, teachers or other kids"
7.1.U: "Thinks before acting"
7.1.V: "Steals in school, at home or elsewhere"
7.1.W: "Gets along with adults better than with other children"
7.1.X: **"Has many fears; is often anxious" (counted as "anxious" in the antenatal GC study)**
7.1.Y: "Finishes tasks; good concentration span"

7.2 Question 68: "Overall, would you say that you child has problems in any of the following areas?"
7.3 Question 69: "Is your kid affected in its daily life by any of these problems (mood, concentration, behavior, social intercourse)?"
7.4 Question 70: "Do any of these problems (s.a.) cause difficulties for your family?"
7.5 Question 71: "To which extent do these issues also apply to you?"

8. Life conditions (questions 72-89)
9. Health behaviors (questions 90-104)
10. Used health services (questions 105-111)
11. Health insurance (questions 112-115)
12. Parents' occupation and career (questions 116-126)
13. Parents' income (questions 127-128)

Survey „*Deutsches Frühgeborenen-Netzwerk (German Neonatal Network, GNN)*“

Education Sheet for Parents

Dear parents,

your child was born prematurely, and small for date- with a birth weight less than 1,500 g. We know that many babies can be discharged from hospital, even if they have been born small for date, albeit after an amount of time which appears excessive to the parents. Unfortunately, there is very little valid data, available in Germany, regarding the development of these small-for-date and premature babies in the long-term. The effect of prematurity and low birth weight on the long-term physical and mental development of these babies is particularly unclear. Apart from the low birth weight and prematurity itself, the influence of other factors; such as currently available therapeutic treatments, the genetic constitution and environmental factors on the development of these children is also not known.

What is the purpose of the survey?

The main objective of our survey is to determine the factors, which influence positively the long term progress and development of these babies. This is possible only if we are aware of the exact nature of the treatment, environmental factors and genetic imprinting, which affect the long term growth and development of premature babies. If these are known, it is possible to implement these treatment methods and environmental factors systematically and specifically. This may lead to the future creation of drugs, which simulate particularly favourable genetic characteristics as well as make it possible to eliminate the factors which have an adverse effect. Finally, our survey aims to discover improved treatment modalities, in order to improve the outcome of underweight and premature babies. In order to maintain confidentiality, the data is collected and used exclusively for this survey.

How is the survey carried out?

The data will be collected at three key stages:

1. During the baby’s stay in hospital immediately following birth

   During the stay at hospital all the essential clinical data of your child (e. g. birth weight and weight at discharge from hospital) will be recorded by the physicians. In order to also examine and analyse the possible genetic influence on the development of the baby, we request your permission to collect two smears of the oral mucosa (the inside of the mouth) of both mother and child, as well as a short piece of the umbilical cord. The smears are taken with a sterile cotton pad and the procedure doesn’t cause pain or distress, even to the small baby. The piece of the umbilical cord is obtained while cutting it during the initial medical care of the premature baby. The cord would otherwise be discarded. The human material will not be used for any additional tests and all data protection requirements will be adhered to strictly. We will not ask for blood collection at any point during the survey.

2. At the age of two years

   At the age of two years, the motor and neuro development of your child is tested. This examination is recommended anyway for all babies weighing less than 1.500 g at birth; i.e. this is not an examination specially carried out for the purpose of this survey.

3. At six years of age
   At six years of age, we plan to examine the mental, intellectual and physical developmental progress (including visual tests).

The follow-up examinations at the age of two and six years will take place in the hospitals where your child has been medically supervised from birth; meaning that you do not have to travel long distances in order to participate in the survey. The results of the follow-up examinations will be discussed immediately afterwards.
Furthermore we will forward to you a questionnaire for parents at all three stages of the survey (during the child's initial hospital stay and at the age of two and six years). These parents' questionnaires have already been answered by more than 17,000 parents, whose children were not born prematurely. As a result, this survey will enable us to find out if the prematurely born infants differ from those born at term; regarding their nutrition, the frequency of consultations etc. We will forward the pseudonymised (i. e. only identified by a number) sheets directly to your private address so that you may complete the questionnaire at your own convenience.

Who takes part in the survey? Data storage
Currently, our survey is the largest one on the long-term development of premature babies that has been conducted in Germany. 50 neonatal departments throughout the country are involved in our survey; and we intend to include about 20.000 neonates over a period of 10 years. We plan on storing the anonymised data over a period of at least 18 years, until the data from the follow-up examinations of the last child enrolled in the survey are completed.

Data protection
It is particularly important for us to protect the personal data of the children participating in the survey. All data will therefore be stored in a pseudonymised format (i. e. all personal data, such as the name and address of participating children are substituted for by a number). We will thus not be in a position to communicate individual results of the genetic examinations to you.
In order to invite you to the follow-up examinations in good time, we request permission to store your address. This address will be verified only with your respective mentoring paediatric clinic and with the registration office; without it being passed to a third party. Both the address data file and the data file including clinical data are protected by extra passwords and encryption, in order to ensure that there is no unauthorised access. It is very important to us to keep you informed about the progress of our survey, and we would therefore ask for your approval to send you a brief report, informing you of the study's progress once a year (on your child's birthday).
All data are completely anonymised immediately after the examination at 6 years of age, i. e. any personal data are completely deleted.
It must be clarified, that participation in this survey is entirely voluntary: no disadvantage, neither to you nor to your child will result from not taking part in it. It will also be possible to withdraw your participation at any point prior to the examination at six years of age, resulting in the destruction of all

data (personal and clinical), and all samples. After the final examination, all personal data will anyway erased.
In the case that you wish to withdraw your participation in this survey, please write to:
The „German Neonatal Network, GNN (Deutsches Frühgeborenen Netzwerk), Klinik für Kinder- und Jugendmedizin der Universität Lübeck, 23538 Lübeck".

Publication of the results of the survey

By principle, no data about clinics or individual infants will be published, or passed on to a third party. All clinics participating in the survey will receive regular reports on the development and progress of the premature infants that they are caring for. In order to inform as many medical scientists and professionals in the field, as well as others with interest in the results of our assignment, overall conclusions and results will be published in scientific journals, or on the internet.

Financing of the survey

This study is funded exclusively by public subsidies from the Bundesministerium für Bildung und Forschung (German Federal Ministry for Education and Research). It may the case, that in the future, the results of the survey will have a commercial use; wherein any profits are reserved for the use of the researchers/scientists and university participating in the survey. Your written consent agrees that any financial demands/claims, resulting from the potential commercialisation of the research will be refrained from.

Who is responsible for the content and carrying out of the survey?

Many medical professionals have taken an active role in this survey. The topic has been discussed with representatives from the professional associations for Neonatology, Perinatology and Social Paediatrics respectively; as well as numerous experts for statistical data evaluation. Prior to commencing with the survey, many parents with premature children had been interviewed; and the survey itself has been cleared by „Das frühgeborene Kind e. V." (German Federal Association), the largest association for parents with premature infants. Prof. Dr. Wolfgang Göpel (Klinik für Kinder- und Jugendmedizin, Universität Lübeck, 23538 Lübeck), the executive of the survey, is responsible for its implementation and realisation. Should the case be that any damages are inflicted upon a participant of the survey- during its progress, and on account of the behaviour of an employee of the Universitätsklinikums Schleswig-Holstein (UK S-H), the UK S-H will be held liable for any such issues.
By carrying out this survey, we hope to better support small-for-date and premature infants in the future. In the case that there remain any questions regarding this survey, please do not hesitate to contact the physician treating your child, or one of the physicians responsible for the survey.

Prof. Dr. med. W. Göpel
(Studienzentrale Universitäts-
Kinderklinik Lübeck)

Survey „*Deutsches Frühgeborenen-Netzwerk (German Neonatal Network, GNN)*"

Declaration of Consent

Herewith I/we give my/our agreement to the participation of my/our child/children

____ born on ___________________

at the survey „Deutsches Frühgeborenen-Netzwerk (German Neonatal Network)"

I have received, read and understood the written information for patients about the survey mentioned above. Purpose and issue of the survey where explained to me in detail – orally and written.

It was pointed out to me that the participation of my child is voluntarily. I have the right to stop/cease the participation at any time and without giving reasons devoid of disadvantages for me or my child.

I was explained and I agree that the clinical progression data of my child and my address are transferred to the survey centre (Paediatric Clinic of the University, Kinderklinik der Universität) and that my address (f. e. in case of a change of residence) will be adjusted at the clinic/hospital treating my child or at the registration office.

I was informed that the taken samples are exclusively used in a pseudonymised or anonymised form in order to research the influencing factors on diseases of neonatal infants and preterm birth. The information about the results of the genetic data cannot be forwarded neither to me nor to the doctor in attendance of my child.

I/we had the opportunity to asking all my/our questions which have been answered satisfactorily and completely.

____

Place, Date Signature of the parent(s)

____

Place, Date Informing doctor (name) Informing doctor (signature)

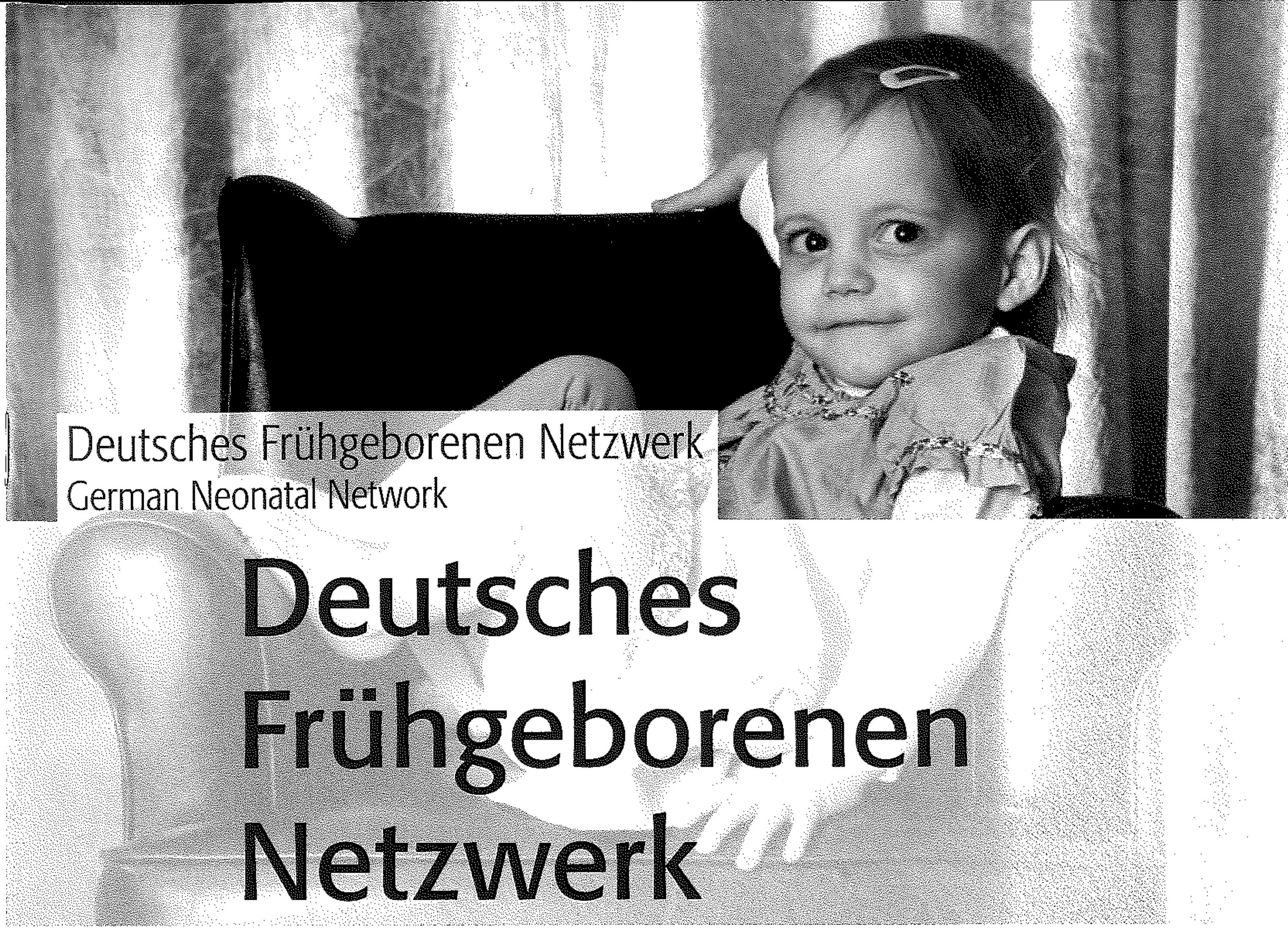

Deutsches Frühgeborenen Netzwerk
German Neonatal Network

Deutsches Frühgeborenen Netzwerk

Mariana

Studie zur langfristigen Entwicklung von sehr kleinen Frühgeborenen

GNN
German Neonatal Network

Elternfragebogen

Liebe Eltern,

mit Ihrem Kind nehmen Sie an einer wichtigen Untersuchung teil.
Sie soll Daten und Erkenntnisse gewinnen und dadurch die gesundheitliche Situation und die Entwicklung der Frühgeborenen in Deutschland verbessern. Sie haben damit eine verantwortungsvolle Aufgabe angenommen.
Dafür danken wir Ihnen.

Sie haben bereits mehrere Fragebögen erhalten. Wir möchten Sie erneut bitten, diesen etwas umfangreicheren Fragebogen auszufüllen und an uns zurück zu senden.

Zunächst befragen wir Sie zur gesundheitlichen Situation Ihres Kindes. Dazu gehören auch Themen wie Therapien, Lebensbedingungen und Verhalten.
Da nicht alle Kinder mit Ihren leiblichen Eltern zusammenleben, bezieht sich die Bezeichnung „Mutter " oder „Vater" in dem Fragebogen auch auf Personen, die an die Stelle der leiblichen Eltern treten (z. B. der Lebenspartner der Mutter, gleichgeschlechtliche Partnerschaft).

- Bitte beantworten Sie die Fragen vollständig.
- Überspringen Sie eine Frage nur, wenn für Sie der Hinweis gilt: ➲ „weiter mit Frage...".
- Kreuzen Sie bei jeder Frage das an oder schreiben Sie das hinein, was auf Sie/ Ihr Kind zutrifft.

Alle Ihre Informationen und Antworten unterliegen den Vorschriften des Datenschutzes. Ihre Angaben werden pseudonymisiert ausgewertet. Niemand kann feststellen, wer welche Angaben gemacht hat.
Bei Fragen oder Unklarheiten können Sie sich jederzeit an uns wenden.

Wir danken Ihnen sehr für Ihre Mithilfe!

Team des Deutschen Frühgeborenen-Netzwerkes
Klinik für Kinder- und Jugendmedizin
Universität zu Lübeck | UKSH | Ratzeburger Allee 160 | 23538 Lübeck
Telefon: **0451 500-43050** | Internet: **www.vlbw.de** | E-Mail: **gnn@uksh.de**

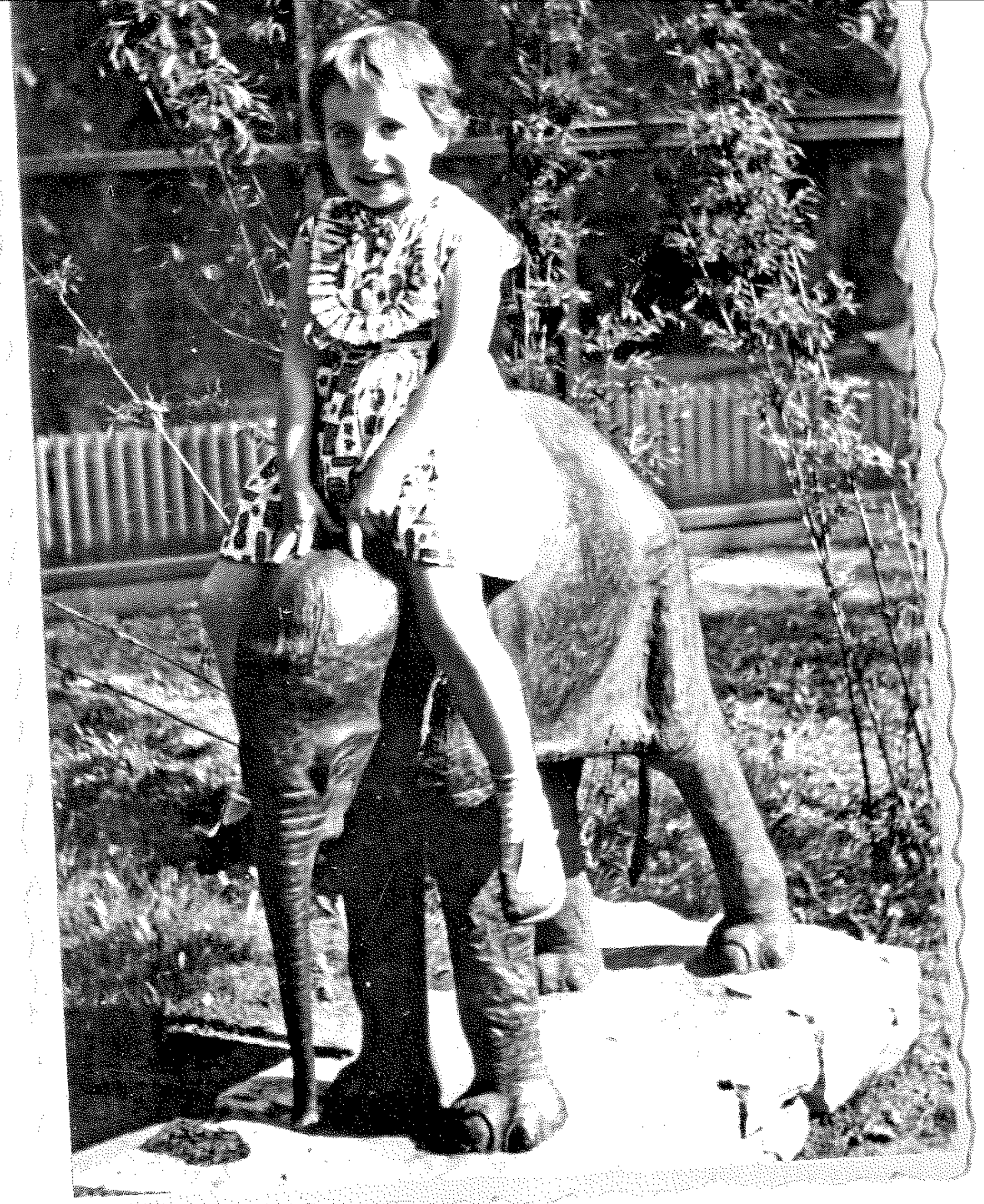

Das bin ich, 5 Jahre alt, bei einem Zoobesuch.

Zoobesuche haben mir und meinen Geschwistern immer sehr gut gefallen, wie bestimmt allen Kindern.

So, nun wissen Sie was von mir, als ich klein war.

Jetzt sind Sie an der Reihe. Ihre Angaben werden helfen, die medizinische Versorgung und Gesundheitsvorsorge von Frühgeborenen zu verbessern.
Es geht um die Steigerung der Lebensqualität.
Die Ihres Kindes und zukünftiger Generationen.

Petra Sperner
Study-Nurse
Deutsches Frühgeborenen Netzwerk
Universität zu Lübeck

Angaben zu den Personen

1 Wann ist Ihr Kind geboren?

Monat | Jahr

2 Welches Geschlecht hat Ihr Kind?

- ○ Männlich
- ○ Weiblich

3 Bei wem lebt Ihr Kind hauptsächlich?

- ○ Leibliche Eltern im gemeinsamen Haushalt
- ○ Zu etwa gleichen Teilen bei Mutter und Vater in getrennten Haushalten
- ○ Mutter mit deren Partner/in im Haushalt
- ○ Mutter ohne Partner/in im Haushalt
- ○ Vater mit dessen Partner/in im Haushalt
- ○ Vater ohne Partner/in im Haushalt
- ○ Großeltern oder andere Verwandte
- ○ Pflegeeltern/Adoptiveltern
- ○ In einem Heim
- ○ Woanders, und zwar: ....

4 Wer beantwortet diesen Fragebogen?

- ○ Mutter
- ○ Vater
- ○ Mutter und Vater
- ○ Großeltern, andere Verwandte
- ○ Pflegeeltern/Adoptiveltern
- ○ Betreuer/in

5 Haben Sie einen festen Partner bzw. eine feste Partnerin?

○ Ja ○ Nein → *Wenn nein, weiter zu Frage 6*

Leben Sie mit ihm bzw. ihr zusammen?

○ Ja ○ Nein

6 Welchen Familienstand haben Sie?

- ○ Verheiratet
- ○ Verheiratet, getrennt lebend
- ○ Eingetragene Lebenspartnerschaft (gleichgeschlechtlich)
- ○ Eingetragene Lebenspartnerschaft (gleichgeschlechtlich), getrennt lebend
- ○ Ledig
- ○ Geschieden
- ○ Verwitwet

7 Wie groß sind Sie und wie viel wiegen Sie?
Bitte für die Elternteile angeben, bei denen Ihr Kind haupsächlich lebt.

	Mutter		Vater	
Größe:		cm		cm
Gewicht:		kg		kg

8 Welche Staatsangehörigkeit(en) haben Sie?
Bitte für beide Elternteile angeben. Mehrere Angaben sind möglich.

Mutter:

- ☐ Deutsch
- ☐ Andere Staatsangehörigkeit → Welche? ....

Vater:

- ☐ Deutsch
- ☐ Andere Staatsangehörigkeit → Welche? ....

9 **Wenn Sie nicht die deutsche Staatsangehörigkeit haben, verfügen Sie derzeit über einen unbefristeten Aufenthaltsstatus?**
Bitte für beide Elternteile angeben.

Mutter:

○ Ja ○ Nein

Vater:

○ Ja ○ Nein

10 **In welchem Land sind Sie geboren?**
Bitte für beide Elternteile angeben.

Mutter:

○ In Deutschland

○ In einem anderen Land → In welchem? ....

Vater:

○ In Deutschland

○ In einem anderen Land → In welchem? ....

11 **Seit wann leben Sie hauptsächlich in Deutschland?**
Bitte für beide Elternteile angeben.

Mutter:

○ Seit meiner Geburt → Seit (Jahreszahl) ☐☐☐☐

Vater:

○ Seit meiner Geburt → Seit (Jahreszahl) ☐☐☐☐

12 **Welche Sprachen werden bei Ihnen zu Hause gesprochen?**
Mehrere Angaben sind möglich.

☐ Deutsch

☐ Andere Sprachen: → Welche? 1. ....

2. ....

13 **Sofern Sie aus einem anderen Land nach Deutschland zugewandert sind, zu welcher der folgenden Zuwanderungsgruppen gehören Sie?**
Bitte für beide Elternteile angeben.

Mutter:

○ Asylbewerberin

○ Asylberechtigte

○ Kriegsflüchtling

○ Kontingentflüchtling

○ Bürgerin eines EU-Staates

○ Ehegattin oder Kind einer bereits in Deutschland lebenden Person

○ Gastarbeiterin, Vertragsarbeiterin oder Ausländische Arbeitnehmerin

○ Deutschstämmige/(Spät-)Aussiedlerin

○ Studentin

○ Sonstige Gruppe

Vater:

○ Asylbewerber

○ Asylberechtigter

○ Kriegsflüchtling

○ Kontingentflüchtling

○ Bürger eines EU-Staates

○ Ehegatte oder Kind einer bereits in Deutschland lebenden Person

○ Gastarbeiter, Vertragsarbeiter oder Ausländischer Arbeitnehmer

○ Deutschstämmiger/(Spät-)Aussiedler

○ Student

○ Sonstige Gruppe

14 **Wie viele Personen leben insgesamt in dem Haushalt, in dem Ihr Kind hauptsächlich lebt?**

Gemeint sind alle Haushaltsmitglieder – Sie selbst und Ihr Kind eingeschlossen –, die ständig in diesem Haushalt gemeinsam wohnen und wirtschaften, egal ob verwandt oder nicht verwandt.
*Lebt Ihr Kind in etwa zu gleichen Teilen bei Mutter und Vater in getrennten Haushalten, machen Sie bitte **nur zu Ihrem Haushalt** Angaben.*

Insgesamt ☐☐ Personen

15 **Welche Personen sind das?**

Nennen Sie bitte für jede Person die Beziehung (siehe Beispiel), in der sie zu Ihrem Kind steht. Bitte schreiben Sie auch das Alter der Person dazu.

Beispiel: Personen im Haushalt

		Alter
1.	Ihr Kind	8
2.	Ich (Mutter)	33
3.	Stiefvater	34
4.	Bruder	5

Ihre Angaben: Personen in Ihrem Haushalt

		Alter
1.	Ihr Kind	
2.		
3.		
4.		
5.		
6.		
7.		
8.		
9.		
10.		

16 **Wie viele leibliche Geschwister/leibliche Halbgeschwister hat Ihr Kind?**

Es ist ein Einzelkind ○
Es ist ein Zwilling/Mehrling ○
Es hat ☐☐ leibliche ältere Geschwister
Es hat ☐☐ leibliche jüngere Geschwister
Es hat ☐☐ leibliche gleichaltrige Geschwister

Gesundheitszustand des Kindes

17 **Wie würden Sie den Gesundheitszustand Ihres Kindes im Allgemeinen beschreiben?**

○ Sehr gut ○ Gut ○ Mittelmäßig ○ Schlecht ○ Sehr schlecht

18 **Hat Ihr Kind eine oder mehrere lang andauernde, chronische Krankheiten Oder Gesundheitsprobleme?**

Chronische Krankheiten sind lang andauernde Erkrankungen, die regelmäßiger Behandlung und Kontrolle bedürfen, zum Beispiel Asthma, Epilepsie, Diabetes oder Herzerkrankungen.

○ Ja ○ Nein

19 **Ist Ihr Kind in irgendeiner Art und Weise eingeschränkt oder daran gehindert, Dinge zu tun, die die meisten gleichaltrigen Kinder tun können?**

○ Ja ○ Nein → *Weiter mit Frage 20*

Geschieht dies aufgrund einer Krankheit, Verhaltensstörung oder eines anderen gesundheitlichen Problems?

○ Ja ○ Nein

Dauert dieses Problem bereits 12 Monate an oder ist eine Dauer von mindestens 12 Monaten zu erwarten?

○ Ja ○ Nein

20 **Hat Ihr Kind eine vom Versorgungsamt amtlich anerkannte Behinderung?**

O Ja

O Nein → *Weiter mit Frage 21*

Welche Behinderung hat Ihr Kind? ....

Seit welchem Jahr ist die Behinderung anerkannt?

Seit (Jahreszahl) [ ][ ][ ][ ]

Wie viel Prozent beträgt der Grad der Behinderung heute?

Grad der Behinderung [ ][ ][ ] %

21 **Hatte Ihr Kind *jemals* Keuchhusten?**

O Ja ↓

O Nein

O Weiß nicht

} *Weiter mit Frage 22*

In welchem Alter hatte Ihr Kind Keuchhusten?
Bitte nur ein Feld ausfüllen.

Jünger als 1 Jahr, im Alter von [ ][ ] Monaten — *Wenn Alter unter 1 Monat, dann "0" eintragen.*

Im Alter von [ ][ ] Jahren — *Nur vollendete Lebensjahre.*

O Weiß nicht

22 **Hatte Ihr Kind *jemals* Masern?**

O Ja ↓

O Nein

O Weiß nicht

} *Weiter mit Frage 23*

In welchem Alter hatte Ihr Kind Masern?
Bitte nur ein Feld ausfüllen.

Jünger als 1 Jahr, im Alter von [ ][ ] Monaten — *Wenn Alter unter 1 Monat, dann "0" eintragen.*

Im Alter von [ ][ ] Jahren — *Nur vollendete Lebensjahre.*

O Weiß nicht

23 **Hatte Ihr Kind *jemals* Windpocken?**

O Ja ↓

O Nein

O Weiß nicht

} *Weiter mit Frage 24*

In welchem Alter hatte Ihr Kind Windpocken?
Bitte nur ein Feld ausfüllen.

Jünger als 1 Jahr, im Alter von [ ][ ] Monaten — *Wenn Alter unter 1 Monat, dann "0" eintragen.*

Im Alter von [ ][ ] Jahren — *Nur vollendete Lebensjahre.*

O Weiß nicht

24 **Welche der folgenden Krankheiten hatte Ihr Kind** *in den letzten 12 Monaten* **und wie oft?**

Falls Ihr Kind keine dieser Krankheiten hatte, tragen Sie bitte jeweils eine "0" ein.

Erkältung, grippaler Infekt	[ ][ ] Mal
Angina (Mandelentzündung)	[ ][ ] Mal
Herpes-Infektion:<br>Bläschen an Lippen und/oder Nase, Mundfäule<br>(Stomatitis aphthosa)	[ ][ ] Mal
Bronchitis (nicht bei Asthma)	[ ][ ] Mal
Pseudokrupp/Kruppsyndrom	[ ][ ] Mal
Durchfall, Magen-Darm-Infektion	[ ][ ] Mal
Blasen- und/oder Harnwegsentzündung	[ ][ ] Mal
Eitrige Bindehautentzündung (bakterielle Konjunktivitis)	[ ][ ] Mal
Lungenentzündung(Pneumonie)	[ ][ ] Mal
Mittelohrentzündung (Otitis media)	[ ][ ] Mal

35 **Wurde bei Ihrem Kind** *jemals* **eine weitere psychische Erkrankung/Störung diagnostiziert?**

O Ja ↓

Welche Erkrankung bzw. Störung ist das?:

O Nein
O Weiß nicht
} *Weiter mit Frage 39*

36 **Bestand die psychische Erkrankung/Störung** *innerhalb der letzten 12 Monate ?*

O Ja O Nein

In welchem Alter wurde die Diagnose gestellt?
Nicht runden. Nur vollendete Lebensjahre eintragen.

Alter bei Diagnose ☐☐ Jahre

37 **Durch wen wurde die Diagnose gestellt?**

O Arzt O Psychologe O Andere

38 **Wurde Ihr Kind deswegen** *jemals* **mit Medikamenten behandelt?**

O Ja O Nein

39 **Leiden die** *leiblichen* **Eltern unter Allergien?**

O Nein
O Weiß nicht
} *Weiter mit Frage 40*

O Ja ↓

Wenn ja, welche Allergien sind das?
Mehrere Angaben sind möglich.

	Mutter	Vater
Heuschnupfen	☐	☐
Neurodermitis	☐	☐
Allergisches Asthma bronchiale	☐	☐
Kontaktallergie, allergisches Kontaktekzem	☐	☐

Schmerzen

40 **Hatte Ihr Kind folgende Schmerzen** *in den letzten 3 Monaten* **?**

	Ja, einmalig	Ja, wiederholt	Nein
Kopfschmerzen	O	O	O
Rückenschmerzen	O	O	O
Ohrenschmerzen	O	O	O
Augenschmerzen	O	O	O
Bauchschmerzen	O	O	O
Schmerzen im Unterleib	O	O	O
Armschmerzen /Handschmerzen	O	O	O
Beinschmerzen/Fußschmerzen	O	O	O
Schmerzen im Brustkorb	O	O	O
Halsschmerzen	O	O	O
Zahnschmerzen	O	O	O
**Andere Schmerzen, welche?**	O	O	O

41 **Welcher dieser Schmerzen Ihres Kindes war** *in den letzten 3 Monaten* **der Hauptschmerz?**

Bitte hier nur einen Schmerz angeben.

Der Hauptschmerz meines Kindes war: ....

42 **Wenn Ihr Kind** *in den letzten 3 Monaten* **wiederholt Kopfschmerzen hatte, wie oft traten diese auf?**

- O Mein Kind hatte keine wiederholten Kopfschmerzen in den letzten 3 Monaten. → Weiter mit Frage 45

Wiederkehrend:

- O 1 Mal im Monat
- O 2-3 Mal im Monat
- O 1 Mal in der Woche
- O Mehrmals in der Woche
- O Täglich
- O Unregelmäßig, insgesamt ☐☐ Mal in den letzten 3 Monaten

Hatte Ihr Kind auch *in den letzten 7 Tagen* **Kopfschmerzen?**

O Ja O Nein

43 **Stand das Auftreten des Kopfschmerzes mit den unten genannten Ereignissen im Zusammenhang?**

	Ja	Nein
Erkrankung	O	O
Verletzung	O	O
Medizinische Behandlung/Operation	O	O
Lärm	O	O
Sportliche Betätigung	O	O
Wechsel der Betreuungseinrichtung/Schulwechsel	O	O
Trennung/Scheidung der Eltern	O	O
Tod eines Angehörigen	O	O
Umzug	O	O
**Mit etwas anderem, und zwar:**	O	O

44 **Wegen der Kopfschmerzen...**

	Nie	Einmal	Manchmal	Häufig	Immer
...hat mein Kind Medikamente eingenommen	O	O	O	O	O
...habe ich mit meinem Kind einen Arzt aufgesucht	O	O	O	O	O
...konnte mein Kind nicht in die Kita/Schule gehen	O	O	O	O	O
...konnte mein Kind seine Freunde nicht treffen	O	O	O	O	O
...hatte mein Kind keinen Appetit/konnte es nicht essen	O	O	O	O	O
...hatte mein Kind Probleme mit dem Schlaf	O	O	O	O	O
...konnte mein Kind seine Hobbys nicht ausüben	O	O	O	O	O
...konnte ein Elternteil nicht zur Arbeit gehen	O	O	O	O	O
...hat mein Kind sich hingelegt	O	O	O	O	O
**...war mein Kind anders beeinträchtigt. - Worin?:**	O	O	O	O	O
**...hat mein Kind Folgendes getan:**	O	O	O	O	O

Unfälle

45 **Wurde Ihr Kind** *in den letzten 12 Monaten* **wegen einer Verletzung (z.B. durch Unfall, Vergiftung oder Gewalt) von einem Arzt behandelt?**

O Ja ↓ O Nein → *Weiter mit Frage 56*

War(en) diese Verletzung(en) bzw. Vergiftung(en) ...
Mehrere Angaben sind möglich.

- ☐ ...unbeabsichtigt, also ein Unfall? → Weiter mit Frage 46
- ☐ ...Folge von Gewalt bei einer tätlichen Auseinandersetzung? } *Weiter mit Frage 56*
- ☐ ...Folge einer absichtlichen Selbstverletzung? } *Weiter mit Frage 56*

Im Folgenden geht es allein um die unbeabsichtigten, also unfallbedingten Verletzungen Ihres Kindes in den letzten 12 Monaten

46 **Wie viele Unfälle mit Verletzungen oder Vergiftungen, die ärztlich behandelt wurden, hatte Ihr Kind insgesamt** *in den letzten 12 Monaten?*

Anzahl der Verletzungen/Vergiftungen ☐☐

47 **Denken Sie nun bitte an die letzte Unfallverletzung/Vergiftung Ihres Kindes** *in den letzten 12 Monaten,* **die ärztlich behandelt wurde.**

Wo hat sich dieser *letzte* **Unfall ereignet?**

- O Zu Hause oder anderes privates Wohnumfeld (Wohnung, Haus, Garten etc., auch bei Nachbarn, Bekannten)
- O Auf dem Weg zu/von: Tagesmutter/Kita/Kindergarten/Hort/Schule ("Wegeunfall")
- O Bei der Tagesmutter/in der Kita/im Kindergarten/Hort/in der Schule (drinnen oder draußen, inkl. Sport- und Spielstätten, Ausflug/Klassenfahrt)
- O In der Freizeit auf dem Spielplatz
- O In der Freizeit in Sport- oder Freizeitstätte (z.B. Turnhalle, Fußballplatz, Schwimmbad, Freizeitpark)
- O In der Freizeit auf öffentlichen Straßen, Wegen oder Plätzen
- O An einem anderen Ort, und zwar: ....

48 **Ereignete sich die** *letzte* **Unfallverletzung während sportlicher Betätigung?**

Gemeint ist Schulsport, Vereinssport oder Freizeitsport.

O Ja O Nein

49 **Wenn der** *letzte* **Unfall auf Wegen, Straßen oder Plätzen stattfand, z.B. auf dem Weg zur Schule oder in der Freizeit, war Ihr Kind dabei...**

- O ...Fußgänger?
- O ...Fahrradfahrer?
- O ...Fahrer eines Kinderfahrzeugs (z.B. Bobbycar oder Laufrad)?
- O ...unterwegs auf Inline-Skates?
- O ...unterwegs auf einem Roller/Kickboard oder Skateboard?
- O ...Mitfahrer eines Verkehrsmittels (z.B. Fahrrad, Auto, Moped oder Bahn)?
- O ...Anderes, und zwar: ....

50 **Wenn der** *letzte* **Unfall auf Wegen, Straßen oder Plätzen stattfand, z.B. auf dem Weg zur Schule oder in der Freizeit, hat die Polizei den Unfall aufgenommen?**

O Ja O Nein

51 **Was ist beim** *letzten* **Unfall passiert?**

- O Sturz in der Ebene
- O Sturz aus der Höhe (höher als 1 Meter)
- O Zusammenstoß/Zusammenprall → O Mit Person
  - O Mit Gegenstand/Objekt
- O Überbeanspruchung eines Körperteils (z.B. Umknicken beim Laufen, Ellenbogenverrenkung durch Hochziehen des Kindes am Arm)
- O Verbrennen oder Verbrühen (z.B. mit kochendem Wasser)
- O Stich- oder Schnittverletzung (z.B. mit Küchenmesser)
- O Vergiftung (durch Verschlucken oder Einatmen von Substanzen oder durch Hautkontakt)
- O Verschlucken, Einatmen eines Fremdkörpers (auch: Stecken in natürliche Körperöffnung)
- O Verletzung durch ein Tier (z.B. Biss, Stich)
- O Anderes, und zwar: ....

52 **Kam der *letzte* Unfall in Zusammenhang mit einem Produkt bzw. Gegenstand oder mit einem Tier zustande?**

Gemeint ist hier zum Beispiel das offene Fenster oder das Pferd, von dem das Kind stürzt, aber auch das Kochwasser, an dem es sich verbrüht.

○ Ja ○ Nein → *Weiter mit Frage 54*

53 **Welches Produkt oder Tier?**

Bitte machen Sie nur ein Kreuz.

○ Kinderfahrzeug (z.B. Kinderdreirad, Kettcar)
○ Spielplatzausstattung (z.B. Schaukel, Rutsche, Klettergerüst)
○ Softball als Sportausrüstung
○ Trampolin
○ Fahrrad
○ Möbel, Einrichtungsgegenstände
○ Tür, Türschwelle
○ Material (z.B. Glas, Stein)
○ Baum, Pflanze oder Pflanzenteile
○ Haushaltszubehör oder -behältnis (z.B. Messer)
○ Tier, welches? ....
○ Anderes Produkt, welches? ....

54 **Bitte geben Sie an, welche Verletzungen bei Ihrem Kind nach dem *letzten* Unfall ärztlich festgestellt wurden.**

Mehrere Angaben sind möglich.

☐ Schädelprellung, Gehirnerschütterung → Mit Bewusstlosigkeit?: ○ Ja ○ Nein
☐ Prellung (außer Kopf), Verrenkung/Zerrung
☐ Knochenbruch: → ☐ Schädel/Halswirbelsäule ☐ Arm/Hand/Schulter ☐ Rumpf ☐ Bein/Fuß
☐ Offene Wunde
☐ Innere Verletzungen (Brust/Bauch)
☐ Vergiftung, Verätzung
☐ Verbrennung, Verbrühung
☐ Andere, und zwar: ....

55 **Wo wurde die *letzte* Verletzung/Vergiftung Ihres Kindes ärztlich behandelt?**

○ Im Krankenhaus ambulant in der Notaufnahme, Erste Hilfe (ohne Übernachtung) } *Weiter mit Frage 56*
○ In einer Arztpraxis } *Weiter mit Frage 56*
○ Im Krankenhaus stationär (mit Übernachtung)

Wie viele Nächte wurde Ihr Kind stationär im Krankenhaus behandelt?

Anzahl der Nächte ☐☐☐

56 **Trägt Ihr Kind...**

	Ja	Nein	Mein Kind fährt nicht Fahrrad, Inline-Skates oder Skateboard
...einen Helm, wenn es Fahrrad fährt?	○	○	○
...einen Helm, wenn es Inline-Skates oder Skateboard fährt?	○	○	○
...Knie- oder Armschützer, wenn es Inline-Skates oder Skateboard fährt?	○	○	○

Entwicklung und Ernährung

57 **Ist Ihr Kind per Kaiserschnitt zur Welt gekommen?**

- O Ja
- O Nein
- O Weiß nicht

58 **Wurde Ihr Kind *jemals* gestillt und/oder mit abgepumpter Muttermilch ernährt?**

- O Ja
- O Nein } *Weiter mit Frage 59*
- O Weiß nicht } *Weiter mit Frage 59*

Bis zu welchem Lebensmonat haben Sie/wurde Ihr Kind gestillt?
Bitte begonnene Lebensmonate eintragen.

Bis zum ☐☐ Lebensmonat

Wie lange haben Sie/wurde Ihr Kind *voll gestillt*, also ohne zusätzliche Gabe von Flaschennahrung, Säuglingsanfangsnahrung oder Beikost?

- O Es wurde bis zum ☐☐ Lebensmonat voll gestillt
- O Es wurde nie voll gestillt.
- O Weiß nicht

59 **Sind Sie der Ansicht, dass Ihr Kind...**

- O ...viel zu dünn ist?
- O ...ein bisschen zu dünn ist?
- O ...genau das richtige Gewicht hat?
- O ...ein bisschen zu dick ist?
- O ...viel zu dick ist?

Kinderbetreuung

60 **Wird bzw. wurde Ihr Kind vor dem Schuleintritt ausschließlich innerhalb der Familie betreut?**
Gemeint ist ohne Tagesmutter, Kindergarten o.ä. Betreuung.

- O Ja → *Weiter mit Frage 66*
- O Nein

Wie wird bzw. wurde Ihr Kind vor dem Schuleintritt noch betreut?
Mehrere Angaben sind möglich.

- ☐ In einer Kinderkrippe, einem Kindergarten oder einer Kita
- ☐ Von einer Tagesmutter oder einer ähnlichen Tagesbetreuung

61 **Wie alt war Ihr Kind zu Beginn der Betreuung in einer Kinderkrippe, Kindertageseinrichtung oder bei einer Tagesmutter/Tagespflege?**

☐☐ Jahre ☐☐ Monate

62 **Wenn Ihr Kind *nicht ausschließlich* innerhalb der Familie betreut wird, wie lange wird Ihr Kind *täglich* außerhalb der Familie betreut?**

- O 5 Stunden oder weniger
- O Mehr als 5, aber weniger als 8 Stunden
- O 8 Stunden und mehr

Bitte beantworten Sie die nächsten Fragen nur, wenn Ihr Kind in einer Kinderkrippe, einem Kindergarten oder einer Kita betreut wird.

63 **Wie groß ist die Kindergruppe, in der Ihr Kind die meiste Zeit betreut wird?**
Bitte zählen Sie dabei Ihr Kind nicht mit.

Mit ☐☐ Kindern

64 **Erhält/erhielt Ihr Kind eine heilpädagogische Förderung im Kindergarten?**
Gemeint sind Leistungen, die durch die Eingliederungshilfe bewilligt werden, z.B. Frühfördermaßnahme, Integrationskindergartenplatz, heilpädagogische Kleingruppe, Einzelintergrationsmaßnahme, Sprachheilkindergarten o.ä.

- O Ja
- O Nein

65 **Wie würden Sie im Allgemeinen die Qualität der Betreuung Ihres Kindes einschätzen?**

Sehr gut	Gut	Mittelmäßig	Schlecht	Sehr schlecht
O	O	O	O	O

Bitte beantworten Sie die nächste Frage nur, wenn...

...Ihr Kind noch nicht eingeschult wurde,
...keine Kindertageseinrichtung,
...keinen Kindergarten oder
...keine Tagesmutter/Tagespflege

in Anspruch nimmt.

66 **Es gibt verschiedene Gründe, warum Eltern ihre Kinder ausschließlich zu Hause und nicht in einer Einrichtung oder durch eine Tagesmutter/Tagespflege betreuen lassen.**

Aus welchen Gründen wird Ihr Kind ausschließlich zu Hause betreut? Was trifft auf Sie zu?

	Trifft zu	Trifft nicht zu
Weil ich das Kind noch für zu jung halte.	O	O
Weil ich das Kind selbst erziehen möchte.	O	O
Weil es die Möglichkeit gibt, das Kind zu Hause zu betreuen (Elternzeit, Schichtdienst etc.).	O	O
Wegen der anfallenden Kosten.	O	O
Weil ich für das Kind keinen geeigneten Platz gefunden habe.	O	O
Weil das Kind eine chronische Krankheit oder Behinderung hat.	O	O
**Anderes**	O	O

Wohlbefinden

67 **Wie gut treffen die folgenden Beschreibungen auf Ihr Kind zu?**

Bitte berücksichtigen Sie bei Ihren Antworten das Verhalten Ihres Kindes ***in den letzten 6 Monaten.***

	Trifft nicht zu	Trifft teilweise zu	Trifft eindeutig zu
Rücksichtsvoll	O	O	O
Unruhig, überaktiv, kann nicht lange stillsitzen	O	O	O
Klagt häufig über Kopfschmerzen, Bauchschmerzen oder Übelkeit	O	O	O
Teilt gerne mit anderen Kindern (Süßigkeiten, Spielzeug, Buntstifte usw.)	O	O	O
Hat oft Wutanfälle; ist aufbrausend	O	O	O
Einzelgänger; spielt meist alleine	O	O	O
Im Allgemeinen folgsam; macht meist, was Erwachsene verlangen	O	O	O
Hat viele Sorgen; erscheint häufig bedrückt	O	O	O
Hilfsbereit, wenn andere verletzt, krank oder betrübt sind	O	O	O
Ständig zappelig	O	O	O
Hat wenigstens einen guten Freund oder eine gute Freundin	O	O	O
Streitet sich oft mit anderen Kindern oder schikaniert sie	O	O	O
Oft unglücklich oder niedergeschlagen; weint häufig	O	O	O
Im Allgemeinen bei anderen Kindern beliebt	O	O	O
Leicht ablenkbar, unkonzentriert	O	O	O
Nervös oder anklammernd in neuen Situationen; verliert leicht das Selbstvertrauen	O	O	O

... → *Fortsetzung auf Seite 23*

67 ↓ *Bitte hier weiter mit Frage 67*
Bitte berücksichtigen Sie weiterhin das Verhalten Ihres Kindes in den letzten 6 Monaten.

	Trifft nicht zu	Trifft teilweise zu	Trifft eindeutig zu
Lieb zu jüngeren Kindern	O	O	O
Lügt oder mogelt häufig	O	O	O
Wird von anderen gehänselt oder schikaniert	O	O	O
Hilft anderen oft freiwillig, wie z.B. Eltern, Lehrern oder anderen Kindern	O	O	O
Denkt nach, bevor er/sie handelt	O	O	O
Stiehlt zu Hause, in der Schule oder anderswo	O	O	O
Kommt besser mit Erwachsenen aus, als mit anderen Kindern	O	O	O
Hat viele Ängste; fürchtet sich leicht	O	O	O
Führt Aufgaben zu Ende; gute Konzentrationsspanne	O	O	O

68 **Würden Sie sagen, dass Ihr Kind insgesamt gesehen in einem oder mehreren der folgenden Bereiche Schwierigkeiten hat:**
Stimmung, Konzentration, Verhalten oder Umgang mit Anderen?

O Ja, leichte Schwierigkeiten
O Ja, deutliche Schwierigkeiten
O Ja, massive Schwierigkeiten
O Nein, keine Schwierigkeiten → *Weiter mit Frage 71*

Seit wann gibt es diese Schwierigkeiten
(Stimmung, Konzentration, Verhalten oder Umgang mit Anderen)?

Seit weniger als 1 Monat	Seit 6 bis 12 Monaten	Seit 1 bis 5 Monaten	Seit über 1 Jahr
O	O	O	O

Leidet Ihr Kind unter diesen Schwierigkeiten (Stimmung, Konzentration, Verhalten oder Umgang mit Anderen)?

Gar nicht	Kaum	Deutlich	Massiv
O	O	O	O

69 **Wird Ihr Kind durch diese Schwierigkeiten (Stimmung, Konzentration, Verhalten oder Umgang mit Anderen) in einem der folgenden Bereiche des Alltagslebens beeinträchtigt?**

	Gar nicht	Kaum	Deutlich	Schwer
Zu Hause	O	O	O	O
Mit Freunden	O	O	O	O
Im Unterricht (wenn Kind noch nicht in der Schule, dann alternativ auf Kindergarten beziehen)	O	O	O	O
In der Freizeit	O	O	O	O

70 **Stellen diese Schwierigkeiten (Stimmung, Konzentration, Verhalten oder Umgang mit Anderen) eine Belastung für Sie oder die gesamte Familie dar?**

Keine Belastung	Leichte Belastung	Deutliche Belastung	Schwere Belastung
O	O	O	O

71 **Wie ist das bei Ihnen?**

	Stimmt nicht	Stimmt kaum	Stimmt eher	Stimmt genau
In unserer Familie geht jeder auf die Sorgen und Nöte des anderen ein.	O	O	O	O
Am Wochenende geht es bei uns zu Hause häufig ziemlich eintönig und langweilig zu.	O	O	O	O
Wir kommen wirklich alle gut miteinander aus.	O	O	O	O
Wir gehen oft ins Kino, besuchen Sportveranstaltungen oder machen Ausflüge.	O	O	O	O
Bei allem, was wir zu Hause tun, sind wir mit Begeisterung dabei.	O	O	O	O
Bei uns vergeht kein Wochenende, ohne dass wir etwas unternehmen.	O	O	O	O
In unserer Familie hat jeder das Gefühl, dass man ihm zuhört und auf ihn eingeht.	O	O	O	O
Abends und an den Wochenenden unternehmen wir selten etwas, sondern bleiben lieber zu Hause.	O	O	O	O
Bei uns ist man eher großzügig, wenn bestimmte Dinge nicht so hundertprozentig gemacht werden.	O	O	O	O

Lebensbedingungen

Im Folgenden geht es um die Wohnung/das Haus, in der/in dem Ihr Kind hauptsächlich lebt. Wenn Ihr Kind zu gleichen Teilen bei Mutter und Vater in getrennten Haushalten lebt, beziehen Sie die Antwort bitte auf Ihre Wohnung.

72 **Seit wann wohnt Ihr Kind am jetzigen Wohnort?**

- O Seit seiner Geburt
- Seit (Jahreszahl) ☐☐☐☐

73 **Seit wann wohnt Ihr Kind in der jetzigen Wohnung/im jetzigen Haus?**

- O Seit seiner Geburt
- Seit (Jahreszahl) ☐☐☐☐

74 **Handelt es sich bei der Wohnung/bei dem Haus, in der/in dem Ihr Kind hauptsächlich lebt, um...**

- O ...eine gemietete Wohnung?
- O ...ein gemietetes Haus?
- O ...eine Eigentumswohnung?
- O ...ein eigenes Haus?
- O ...ein Untermietverhältnis?
- O ...Sonstiges

75 **In welchem der folgenden Gebäudetypen wohnt Ihr Kind hauptsächlich?**

- O Hochhaus (Gebäude mit 5 oder mehr Stockwerken)
- O Wohnblock (Gebäude mit mehreren Hausnummern und max. 4 Etagen)
- O Mehrfamilienhaus (Gebäude mit 1 Hausnummer und max. 4 Etagen)
- O Zweifamilienhaus, Doppelhaushälfte oder Reihenhaus
- O Einfamilienhaus
- O Bauernhof
- O Sonstiges

76 **Wie viele Zimmer/Wohnräume hat die Wohnung/das Haus, in der/in dem Ihr Kind hauptsächlich lebt?**
*Bitte Küche, Bad/Toilette, getrenntes WC oder Abstell-/Kellerräume **nicht** mitzählen.*

Anzahl der Zimmer: ☐☐

77 **Wie groß ist die Wohnung/Haus, in der Ihr Kind hauptsächlich lebt?**

Größe in Quadratmetern (m²): ☐☐☐☐

78 **Wo schläft Ihr Kind üblicherweise?**

- O Allein im eigenen Kinderzimmer
- O Mit anderen Kindern im Zimmer
- O Im Elternschlafzimmer
- O Mit anderen Personen im Zimmer

79 **Wächst Ihr Kind mit engem Kontakt zu landwirtschaftlich-bäuerlich gehaltenen Tieren auf?**

- O Ja
- O Nein

80 **Gibt es in der Wohnung/ Haus Räume, in denen sich Ihr Kind aufhält, deren Wände und/oder Decken schimmlig sind?**

- O Ja
- O Nein

Gibt es in der Wohnung Räume, in denen sich Ihr Kind aufhält, deren Wände und/oder Decken feucht sind?

- O Ja
- O Nein

81 **Wie würden Sie die Wohngegend beschreiben?**

- O Ein reines Wohngebiet
- O Ein Mischgebiet mit Wohnungen und Geschäften bzw. Gewerbebetrieben
- O Ein Gewerbe- bzw. Industriegebiet mit wenigen Wohnungen

82 **Falls Ihre Wohnung an mehrere Straßen angrenzt, beziehen Sie sich bitte im Folgenden nur auf die am stärksten befahrene Straße. Liegt die Wohnung oder das Haus, in der Ihr Kind hauptsächlich lebt, an einer durch Kraftfahrzeugverkehr...**

- O ...extrem stark befahrenen Durchgangsstraße?
- O ...stark befahrenen Haupt- oder Durchgangsstraße?
- O ...beträchtlich befahrenen Nebenstraße?
- O ...mäßig befahrenen Nebenstraße?
- O ...sehr wenig befahrenen Straße (Anliegerstraße, Weg, verkehrsberuhigte Zone)?

83 **Wenn Sie an die *letzten 12 Monate* denken, wie stark haben Sie sich in der Wohnung/in dem Haus, in der/in dem Ihr Kind hauptsächlich lebt, durch Lärm von den im Folgenden aufgeführten Quellen gestört oder belästigt gefühlt?**

Bitte für jede Lärmquelle ein Kreuz machen, auch wenn die Quelle vielleicht nicht vorhanden ist.

	Gar nicht	Etwas	Mittelmäßig	Stark	Sehr stark
Straßenlärm	O	O	O	O	O
Fluglärm	O	O	O	O	O
Schienenverkehrslärm	O	O	O	O	O
Baulärm	O	O	O	O	O
Nachbarschaftslärm	O	O	O	O	O
Industrie-/Gewerbelärm	O	O	O	O	O
Lärm durch Gaststätten/ Diskotheken	O	O	O	O	O
Lärm von Kinderspielplätzen	O	O	O	O	O
Lärm durch Naturgeräusche (z.B. Bach, Vogel)	O	O	O	O	O
Lärm durch Familienmitglieder in der Wohnung	O	O	O	O	O
**Sonstiger Lärm, und zwar:**	O	O	O	O	O

84 **Gibt es an Ihrem derzeitigen Wohnort Spiel- oder Sportmöglichkeiten, die gut für Ihr Kind zu erreichen sind?**

	Ja	Nein	Weiß nicht
Spielplatz	O	O	O
Sportplatz/Sporthalle	O	O	O
Schwimmbad/Schwimmhalle	O	O	O
Park, öffentliche Grünflächen	O	O	O
Garten	O	O	O

85 **Welche der folgenden Dinge besitzt Ihr Kind persönlich?**
Mehrere Angaben sind möglich.

- ☐ Smartphone
- ☐ Anderes Handy
- ☐ Laptop
- ☐ Tablet-PC
- ☐ PC
- ☐ Spielkonsole
- ☐ iPod oder MP3-Player
- ☐ Keines dieser Dinge

86 **Hat Ihr Kind ein Fernsehgerät in seinem Zimmer?**

O Ja O Nein

Hat Ihr Kind einen Computer (auch Laptop) in seinem Zimmer?

- O Ja, ohne Internetanschluss
- O Ja, mit Internetanschluss
- O Nein

87 **Wie lange beschäftigt sich Ihr Kind durchschnittlich pro Tag mit folgenden Dingen?**

	Stunden (h) pro Tag					
	Mehr als 4 h	3 h bis unter 4 h	2 h bis unter 3 h	1 h bis unter 2 h	Bis zu 1 h	Gar nicht
Fernsehen und Video/DVD (auch über PC/Laptop oder Smartphone)	O	O	O	O	O	O
Spielkonsole oder Computerspiele (auch über Smartphone)	O	O	O	O	O	O
Sonstige Computernutzung und Internet (auch über Smartphone)	O	O	O	O	O	O
Bücher lesen	O	O	O	O	O	O

88 **Rauchen Sie zurzeit?**
Bitte für beide Elternteile angeben.

	Ja, täglich	Ja, gelegentlich	Nein
**Mutter**	O	O	O
**Vater**	O	O	O

Wird in der Gegenwart Ihres Kindes in der Wohnung geraucht?

Täglich	Mehrmals pro Woche	Einmal pro Woche	Seltener	Nie
O	O	O	O	O

89 **Hat die Mutter des Kindes während der Schwangerschaft geraucht?**

Ja, regelmäßig	Ja, ab und zu	Nein, nie
O	O	O

Hat die Mutter des Kindes während der Stillzeit geraucht?

Ja, regelmäßig	Ja, ab und zu	Nein, nie	Hat nicht gestillt
O	O	O	O

Gesundheitsverhalten

Die folgenden Aussagen betreffen die Schlafgewohnheiten Ihres Kindes und mögliche Schwierigkeiten mit dem Schlaf.
Denken Sie bei der Beantwortung der Fragen an einen typischen Monat Ihres Kindes.

90 **Welche ist die übliche Zubettgehzeit Ihres Kindes unter der Woche?**
Bitte geben Sie die Zeit in Stunden und Minuten, z.B. 19:30 an.

☐☐ : ☐☐ Uhr

Welche ist die übliche Uhrzeit, zu der Ihr Kind unter der Woche Gewöhnlich morgens aufwacht bzw. aufgeweckt wird?
Bitte geben Sie die Zeit in Stunden und Minuten, z.B. 06:30 an.

☐☐ : ☐☐ Uhr

91 **Wie viele Stunden Schlaf bekommt Ihr Kind unter der Woche üblicherweise pro Tag?**
Bitte geben Sie die Gesamtschlafmenge aus Nacht und Tag an.

☐☐ Stunden und ☐☐ Minuten

92 **Wie häufig schläft Ihr Kind nach dem Zubettgehen innerhalb von 20 Minuten ein?**

O Gewöhnlich (5-7 Mal/Woche)
O Manchmal (2-4 Mal/Woche)
O Selten (0-1 Mal/Woche)

93 **Wie häufig wacht Ihr Kind mehr als einmal während der Nacht auf?**

O Gewöhnlich (5-7 Mal/Woche)
O Manchmal (2-4 Mal/Woche)
O Selten (0-1 Mal/Woche)

94 **Wie häufig schnarcht Ihr Kind laut?**

- O Gewöhnlich (5-7 Mal/Woche)
- O Manchmal (2-4 Mal/Woche)
- O Selten (0-1 Mal/Woche)

95 **Wie oft putzt sich Ihr Kind die Zähne?**

- O Zweimal täglich oder häufiger
- O Einmal täglich
- O Nicht täglich, aber mehrmals pro Woche
- O Einmal pro Woche oder seltener
- O Gar nicht

96 **In welchen Abständen geht Ihr Kind zur Kontrolle zum Zahnarzt?**

- O Vierteljährlich
- O Halbjährlich
- O Einmal im Jahr
- O Seltener
- O Mein Kind war noch nie beim Zahnarzt.

Ist Ihr Kind in ständiger kieferorthopädischer Behandlung?

O Ja O Nein

97 **Wie oft treiben Sie selbst Sport?**
Denken Sie dabei bitte an die letzten 3 Monate.

	Mutter	Vater
Keine sportliche Betätigung	O	O
Weniger als eine Stunde in der Woche	O	O
Regelmäßig 1-2 Stunden in der Woche	O	O
Regelmäßig 2-4 Stunden in der Woche	O	O
Regelmäßig mehr als vier Stunden in der Woche	O	O

98 **An wie vielen Tagen einer normalen Woche ist Ihr Kind für mindestens 60 Minuten am Tag körperlich aktiv?**

An 7 Tagen	O	An 3 Tagen	O
An 6 Tagen	O	An 2 Tagen	O
An 5 Tagen	O	An einem Tag	O
An 4 Tagen	O	An keinem Tag	O

99 **An wie vielen** *der letzten 7 Tage* **war Ihr Kind für mindestens 60 Minuten am Tag körperlich aktiv?**

An 7 Tagen	O	An 3 Tagen	O
An 6 Tagen	O	An 2 Tagen	O
An 5 Tagen	O	An einem Tag	O
An 4 Tagen	O	An keinem Tag	O

106 **Hat Ihr Arzt/Ihre Ärztin Ihnen von Impfungen für Ihr Kind abgeraten?**

○ Ja ○ Nein → *Weiter mit Frage 107*

↓

Von welcher/welchen Impfung/en hat Ihr Arzt/Ihre Ärztin Ihnen abgeraten?

	Impfung	Begründung
1	....	....
2.	....	....
3.	....	....
4.	....	....

107 **Bitte teilen Sie uns mit, welche niedergelassenen Ärzte/Ärztinnen der folgenden Fachrichtungen Sie für Ihr Kind** *in den letzten 12 Monaten* **wie häufig in Anspruch genommen haben.**

Bitte zählen Sie die Hausbesuche mit, aber keine Arztkontakte im Krankenhaus oder in einer Kur.

Mehrere Angaben sind möglich.

	Ja, in Anspruch genommen		Anzahl der Arztkontakte	
Kinderarzt, Pädiater	☐	→	☐☐	Mal
Praktischer Arzt, Arzt für Allgemeinmedizin	☐	→	☐☐	Mal
Internist (Innere Medizin, z.B. Kardiologe, Pneumologe, Diabetologe)	☐	→	☐☐	Mal
Frauenarzt, Gynäkologe	☐	→	☐☐	Mal
Augenarzt	☐	→	☐☐	Mal
Orthopäde	☐	→	☐☐	Mal
Hals-Nasen-Ohrenarzt	☐	→	☐☐	Mal
Neurologe, Nervenarzt	☐	→	☐☐	Mal

...

107 ↓ *Fortsetzung von Frage 107 Seite 35*

Bitte achten Sie darauf, dass die Angaben die Sie machen im Zeitraum der letzten 12 Monate liegen.
zählen Sie die Hausbesuche mit, aber keine Arztkontakte im Krankenhaus oder in einer Kur.

Mehrere Angaben sind möglich.

	Ja, in Anspruch genommen	Anzahl der Arztkontakte	
Psychiater, Kinder- und Jugendpsychiater, ärztlicher Psychotherapeut	☐	☐☐	Mal
Psychologe, psychologischer Psychotherapeut	☐	☐☐	Mal
Chirurg	☐	☐☐	Mal
Hautarzt, Dermatologe	☐	☐☐	Mal
Röntgenarzt, Radiologe	☐	☐☐	Mal
Urologe	☐	☐☐	Mal
Zahnarzt, Kieferorthopäde	☐	☐☐	Mal
**Sonstiger Arzt, und zwar** (bitte hier keine Therapeuten/Personen eintragen):	☐	☐☐	Mal

Mein Kind war *in den letzten 12 Monaten* **NICHT bei einem niedergelassenen Arzt/einer niedergelassenen Ärztin.** ○

108 **Haben Sie für Ihr Kind** *in den letzten 12 Monaten* **Ambulanzen, Erste-Hilfe-Stationen oder medizinische Behandlungszentren in Krankenhäusern ambulant in Anspruch genommen?**

Hier sind nur Behandlungen ohne Übernachtung im Krankenhaus gemeint.

○ Ja ○ Nein → *Weiter mit Frage 109*

↓

Wie häufig wurde Ihr Kind ambulant behandelt?

Anzahl: ☐☐☐ Mal

109 **War Ihr Kind** *in den letzten 12 Monaten* **zur stationären Behandlung (über Nacht) in einem Krankenhaus aufgenommen?**

○ Ja ○ Nein → *Weiter mit Frage 110*

Wie viele Nächte hat Ihr Kind *in den letzten 12 Monaten* **in einem Krankenhaus verbracht?**

Anzahl der Nächte: ☐☐☐

110 **Welche der nachfolgenden Therapeuten/Therapeutinnen haben Sie für Ihr Kind** *in den letzten 12 Monaten* **in Anspruch genommen und wie häufig?**
Nicht gemeint sind Psychotherapeuten/-therapeutinnen.
Mehrere Angaben sind möglich.

	Ja, in Anspruch genommen	Anzahl Wochen
Heilpraktiker	☐	☐☐
Krankengymnast, Physiotherapeut	☐	☐☐
Logopäde, Sprachtherapeut	☐	☐☐
Ergotherapeut	☐	☐☐
**Sonstige, und zwar?** ______	☐	☐☐

Mein Kind war *in den letzten 12 Monaten* **NICHT bei einem Therapeuten/ einer Therapeutin.** ○

111 **Haben Sie** *in den ersten 2 Lebensjahren* **folgende Angebote mit ihrem Kind In Anspruch genommen?**
Mehrere Angaben sind möglich.

	Ja, in Anspruch genommen	Anzahl Wochen gesamt
Krabbelgruppe	☐	☐☐
DELFI oder PEKIP-Gruppe (Frühförderung in Gruppen)	☐	☐☐
Babyschwimmen	☐	☐☐
Musikalische Förderung	☐	☐☐
**Sonstige, und zwar?** ______	☐	☐☐

Wir haben *in den ersten 2 Lebensjahren* **keine Angebote in Anspruch genommen.** ○

Krankenversicherung

112 **In welcher Krankenversicherung/-kasse ist Ihr Kind versichert?**

☐ **Gesetzliche Krankenversicherung (GKV), und zwar:**

○ Allgemeine Ortskrankenkasse (AOK)
○ Betriebskrankenkasse (BKK)
○ Ersatzkrankenkasse (z.B. Barmer GEK, TK, DAK, KKH)
○ Innungskrankenkasse (IKK)
○ Landwirtschaftliche Krankenkasse, Knappschaft, Deutsche Rentenversicherung, Knappschaft-Bahn-See als Träger der knappschaftlichen Rentenversicherung

☐ **Beihilfe**

☐ **Private Krankenversicherung**
(Hier ist *keine private Zusatzversicherung* zur gesetzlichen Krankenversicherung gemeint.)

☐ **Anderer Anspruch auf Krankenversorgung**
(z.B. freie Heilfürsorge, Sozialhilfeempfänger, Kriegsschadenrentner, Lastenausgleich, Ausländische Krankenkasse)

Falls Sie nicht wissen, wie Ihre Krankenversicherung einzuordnen ist, schreiben Sie bitte den genauen Namen der Krankenkasse auf: ______

Bildung

Bei den folgenden Fragen, die Vater und Mutter betreffen, meinen wir die Personen, die mit dem Kind in einem Haushalt leben. Mit der Bezeichnung „Mutter“ oder „Vater“ sind auch diejenigen Personen gemeint, die an die Stelle der leiblichen Eltern treten, z.B. Lebenspartnerin des Vaters, Stiefvater o.a. .

113 Welchen *höchsten* allgemein bildenden Schulabschluss haben Sie?

* oder vergleichbare Personen im Haushalt

	* Mutter	* Vater
Noch keinen Abschluss (noch Schüler)	○	○
Schule beendet ohne Abschluss	○	○
Haupt-/Volksschule	○	○
Realschule/Mittlere Reife/Mittlerer Schulabschluss (MSA)/ Polytechnische Oberschule (POS)	○	○
Abitur, allgemeine oder fachgebundene Hochschulreife, erweiterte Oberschule (EOS), Fachhochschulreife/ Fachoberschule	○	○
Anderer Schulabschluss (z.B. im Ausland erworben)	○	○

114 Welchen *höchsten* beruflichen Abschluss haben Sie?

* oder vergleichbare Personen im Haushalt

	* Mutter	* Vater
Keinen Abschluss, noch in beruflicher Ausbildung, z.B. Student/in, AZUBI, Berufsvorbereitungsjahr, Praktikant/in	○	○
Keinen Berufsabschluss und nicht in Ausbildung	○	○
Lehre, also beruflich-betriebliche Ausbildung	○	○
Ausbildung an Berufsfachschule, Handelsschule, also beruflich-schulische Ausbildung	○	○
Fachschule, z.B. Meister-, Technikerschule, Berufs- oder Fachakademie	○	○
Fachhochschule, Ingenieurschule	○	○
Universität oder Hochschule	○	○
Anderen Ausbildungsabschluss *(z.B. im Ausland erworben)*	○	○

Bitte beantworten Sie die folgende Frage nur, wenn Sie einen Fachhochschul-, Universitäts-,Hochschulabschluss oder einen anderen Ausbildungsabschluss z.B. im Ausland erworben haben.

115 Mit welchem *höchsten* Hochschulabschluss haben Sie abgeschlossen?

* oder vergleichbare Personen im Haushalt

	* Mutter	* Vater
Mit einer Promotion	○	○
Mit einem Universitätsabschluss, z.B. Diplom, Magister, Staatsexamen, Master	○	○
Mit einem Fachhochschulabschluss z.B. Diplom, Magister	○	○
Mit einem Bachelor an einer Hochschule oder Fachhochschule	○	○

Erwerbstätigkeit/Berufliche Stellung

116 Welche Lebenssituation trifft derzeit überwiegend auf Sie zu?
Kreuzen Sie für Mutter/Vater jeweils bitte nur eine Antwort an.

* oder vergleichbare Personen im Haushalt

	* Mutter	* Vater
Ich bin erwerbstätig in Vollzeit (auch Berufsausbildung oder Selbstständigkeit; ohne Altersteilzeit).	○	○
Ich bin erwerbstätig in Teilzeit (auch Berufsausbildung oder Selbstständigkeit; ohne Altersteilzeit).	○	○
Ich bin geringfügig erwerbstätig (z.B. 450-Euro-Job, Minijob).	○	○
Ich bin in Altersteilzeit (Arbeits- und Freistellungsphase).	○	○
Ich leiste ein freiwilliges soziales/ökologisches/kulturelles Jahr.	○	○
Ich bin freiwillig Wehrdienst- oder Bundesfreiwilligendienstleistende/r.	○	○
Ich bin arbeitslos oder arbeitssuchend bei der Agentur für Arbeit gemeldet.	○	○
Ich bin im Ruhestand oder Vorruhestand.	○	○
Ich bin dauerhaft erwerbsunfähig.	○	○
Ich bin Schüler/in, Student/in, Praktikant/in, Teilnehmer/in an Fortbildungsmaßnahmen.	○	○
Ich bin in Elternzeit.	○	○
Ich bin als Hausfrau/Hausmann tätig, betreue Kinder oder hilfsbedürftige Personen.	○	○
Ich bin aus anderen Gründen nicht erwerbstätig.	○	○

117 **Falls Sie derzeit arbeitslos sind, wie lange sind Sie bereits arbeitslos gemeldet?**

* **Mutter:**	☐☐	Jahre	und	☐☐	Monate
* **Vater:**	☐☐	Jahre	und	☐☐	Monate

* oder vergleichbare Personen im Haushalt

118 **Wie viele Stunden pro Woche waren Sie *im letzten Monat* durchschnittlich erwerbstätig?**

Bitte zählen Sie bei mehreren bezahlten Tätigkeiten alle tatsächlich geleisteten Stunden Zusammen. Wenn Sie im letzten Monat nicht erwerbstätig waren, tragen Sie bitte eine "0" ein.

* **Mutter:**	☐☐	Stunden pro Woche
* **Vater:**	☐☐	Stunden pro Woche

* oder vergleichbare Personen im Haushalt

119 **Welche berufliche Stellung haben Sie in Ihrer Haupterwerbstätigkeit?**

Wenn Sie derzeit nicht oder nicht mehr berufstätig sind, nennen Sie bitte die berufliche Stellung, die Sie zuletzt innehatten.

Beachten Sie, dass auch ein Minijob eine Haupterwerbstätigkeit, z.B. für Rentner, Hausfrauen, Studierende, oder Arbeitslose, sein kann. In diesem Fall gelten Sie als Angestellter.

Selbstständige, die nur mithelfende Familienangehörige beschäftigen, geben "selbstständig erwerbstätig ohne Mitarbeiter" an.

* oder vergleichbare Personen im Haushalt

Kreuzen Sie für Mutter/Vater jeweils bitte nur eine Antwort an.	* **Mutter**	* **Vater**
Ich bin Angestellte/r.	O	O
Ich bin Arbeiter/in.	O	O
Ich bin Beamtin/Beamter (auch Anwärter/in).	O	O
Ich bin Landwirt/in im Haupterwerb.	O	O
Ich bin selbstständig erwerbstätig und habe Mitarbeiter.	O	O
Ich bin selbstständig erwerbstätig ohne Mitarbeiter.	O	O
Ich bin mithelfende/r Familienangehörige/r (unbezahlt).	O	O
Ich bin Auszubildende/r (auch Praktikant/in, Volontär/in).	O	O
Ich bin freiwillig Wehrdienst- oder Bundesfreiwilligendienstleistende/r.	O	O
Ich leiste ein freiwilliges soziales/ökologisches/kulturelles Jahr.	O	O
Ich bin noch nie erwerbstätig gewesen.	O	O

120 **Nehmen Sie eine Führungsaufgabe wahr, d.h. sind Sie Mitarbeitern/ Mitarbeiterinnen gegenüber weisungsbefugt, die keine Auszubildenden sind?**

* oder vergleichbare Personen im Haushalt

	* Mutter	* Vater
Ja, als Führungskraft *(mit Entscheidungsbefugnis über Personal, Budget und Strategie)*	O	O
Ja, als Aufsichtskraft *(Anleiten und Beaufsichtigen von Personal, Verteilen und Kontrollieren von Arbeit)*	O	O
Nein	O	O

121 **Welche Art von Beschäftigungsverhältnis haben Sie in Ihrer Haupterwerbstätigkeit?**

Wenn Sie derzeit nicht oder nicht mehr berufstätig sind, nennen Sie bitte die berufliche Stellung, die Sie zuletzt innehatten.

* oder vergleichbare Personen im Haushalt

	* Mutter	* Vater
Unbefristetes Beschäftigungsverhältnis	O	O
Befristetes Beschäftigungsverhältnis	O	O

Wenn Sie noch nie berufstätig gewesen sind bzw. derzeit nicht oder nicht mehr berufstätig sind → *Bitte weiter mit Frage 128*

122 **Welchen Beruf üben Sie derzeit aus?**

Geben Sie die genaue Berufsbezeichnung an, nicht den Ausbildungsabschluss oder Rang.
Zum Beispiel:
- Blumenverkäuferin (nicht Verkäuferin)
- Maurer (nicht Bauarbeiter)
- Zollsachbearbeiterin (nicht Beamtin)
- Unternehmensberaterin (nicht Betriebswirtin)

* **Mutter** ....

* **Vater** ....

* oder vergleichbare Personen im Haushalt

123 **Um die Einordnung Ihres Berufes zu erleichtern, geben Sie bitte zusätzliche Erläuterungen in Stichworten an.**

Zum Beispiel:
- *Beratung, Verkauf, Verpacken von Pflanzen (als Blumenverkäuferin)*
- *Zollfahndung, Einsatzplanung, Pressearbeit (als Zollbeamter)*
- *Wartung, Instandsetzung, Ausrüstung von Kraftfahrzeugen, Leitung der Werkstatt (als KFZ-Mechaniker)*

Falls Sie Führungsaufgaben wahrnehmen, vermerken Sie dies auch.

*** Mutter** ....

....

....

....

*** Vater** ....

....

....

....

* oder vergleichbare Personen im Haushalt

124 **Arbeiten Sie in Ihrer Haupterwerbstätigkeit im öffentlichen Dienst?**

	ja	nein
*** Mutter**	O	O
*** Vater**	O	O

* oder vergleichbare Personen im Haushalt

125 **Bitte ordnen Sie den Betrieb, in dem Sie tätig sind, einer Branche/einem Wirtschaftszweig zu.**

Richten Sie sich nach dem wirtschaftlichen Schwerpunkt des Betriebes (nicht des gesamten Unternehmens).

Für Selbstständige und Nebenjobber/-innen:
Falls Sie in keinem Betrieb tätig sind, geben Sie die Branche/den Wirtschaftszweig an, in dem Sie als Selbstständige/r oder Nebenjobber/in schwerpunktmäßig tätig sind.
Kreuzen Sie für Mutter/Vater jeweils bitte nur eine Antwort an.

* oder vergleichbare Personen im Haushalt

	*** Mutter**	*** Vater**
**Land- und Forstwirtschaft, Fischerei**	O	O
**Verarbeitendes Gewerbe/Herstellung von Waren, Bergbau und Gewinnung von Steinen und Erden, sonstige Industrie**		
Bergbau und Gewinnung von Erdöl, Erdgas, Steinen und Erden	O	O
Verarbeitendes Gewerbe/Herstellung von Waren (z.B. Lebensmittel, Textilien, Elektronik, Maschinen, Fahrzeuge, Mineralölverarbeitung, Druckerzeugnisse)	O	O
Reparatur und Installation von Maschinen und Ausrüstungen	O	O
Energieversorgung	O	O
Wasserversorgung; Abwasser- und Abfallentsorgung und Beseitigung von Umweltverschmutzung	O	O
**Baugewerbe, Hoch- und Tiefbau**	O	O
**Handel, Verkehr und Lagerei, Gastgewerbe/Beherbergung und Gastronomie**		
Groß- und Einzelhandel; Instandhaltung und Reparatur von Kraftfahrzeugen	O	O
Personen- und Güterverkehr; Lagerei (auch Post- und Kurierdienst)	O	O
Gastgewerbe/Beherbergung und Gastronomie	O	O
**Information und Kommunikation** (z.B. Telekommunikation, Dienstleistungen der Informationstechnologie, Medien und Verlagswesen)	O	O

... → *Fortsetzung der Frage auf Seite 45*

* oder vergleichbare Personen im Haushalt

125 ↓ *Fortsetzung von Seite 44*

	* Mutter	* Vater
**Banken/Finanz- und Versicherungsdienstleister**	O	O
**Grundstücks- und Wohnungswesen**	O	O
**Freiberufliche, wissenschaftliche und technische Dienstleistungen sowie sonstige wirtschaftliche Dienstleistungen**		
Freiberufliche, wissenschaftliche und technische Dienstleistungen (z.B. Unternehmens-, Rechts- und Steuerberatung, Wirtschaftsprüfung, Architektur-/Ingenieurbüro, Forschungs- und Entwicklungsleistungen, Werbung und Marktforschung)	O	O
Sonstige wirtschaftliche Dienstleistungen für Unternehmen und Privatpersonen (z.B. Vermietung beweglicher Sachen, Sicherheitsdienst, Gebäudebetreuung/ -reinigung, Garten- und Landschaftsbau,Reisebüro/-veranstalter, Vermittlung von Arbeitskräften,Sekretariatsdienste, Messeveranstalter)	O	O
**Öffentliche Verwaltung, Verteidigung, Sozialversicherung, Erziehung und Unterricht, Gesundheits- und Sozialwesen**		
Öffentliche Verwaltung, Gerichte, Öffentliche Sicherheit und Ordnung, Verteidigung, Sozialversicherung	O	O
Erziehung und Unterricht (z.B. Hochschule, Schule, sonstige Schule (auch Fahrschule), Kindergarten)	O	O
Gesundheits- und Sozialwesen (z.B. Krankenhaus, Arztpraxis, Alten- und Pflegeheim, Behindertenwerkstatt)	O	O
**Sonstige Dienstleistungen**		
Sonstige überwiegend personenbezogene Dienstleistungen; allgemeine Reparaturen von Waren und Geräten (z.B. Friseur- und Kosmetiksalon, Wäscherei, Solarium/Sauna/Bad, Bestattung)	O	O
Kunst, Unterhaltung, Sport und Erholung (z.B. Theater, Museum, schriftstellerische Tätigkeit, Sport- und Fitnesszentrum)	O	O
Gewerkschaft, Verband, Partei und sonstige Interessenvertretung, kirchliche und religiöse Vereinigung	O	O
Konsulat, Botschaft, internationale und supranationale Organisation	O	O
Privater Haushalt mit Beschäftigten	O	O

126 **Wie viele Personen arbeiten in diesem Betrieb?**

Zählen Sie bei Unternehmen mit mehreren Betrieben (z.B. Werk, Filiale, Niederlassung) nur die Personen, die in Ihrem Betrieb (örtliche Einheit) arbeiten. Zählen Sie auch sich selbst, alle Teilzeitkräfte sowie Auszubildende und tätige Firmeninhaber dazu.

	* Mutter	* Vater
Bis einschließlich 10 Personen	O	O
11 bis einschließlich 19 Personen	O	O
20 bis einschließlich 49 Personen	O	O
50 Personen oder mehr	O	O

* oder vergleichbare Personen im Haushalt

Einkommen

127 **Wie hoch ist in etwa das monatliche Netto-Einkommen Ihres Haushalts insgesamt?**

Bitte zählen Sie die monatlichen Einkommen aller Haushaltsmitglieder (einschließlich Elterngeld, Kindergeld usw.) nach Abzug von Steuern und Sozialabgaben zusammen.

- Euro -

Das monatliche Nettoeinkommen unseres Haushalts beträgt etwa: ____________

128 **Falls Sie den Betrag nicht kennen oder nicht nennen möchten, schätzen Sie ihn bitte anhand der folgenden Liste:**

- O unter 500 Euro pro Monat
- O 500 bis unter 750 Euro
- O 750 bis unter 1.000 Euro
- O 1.000 bis unter 1.250 Euro
- O 1.250 bis unter 1.500 Euro
- O 1.500 bis unter 1.750 Euro
- O 1.750 bis unter 2.000 Euro
- O 2.000 bis unter 2.250 Euro
- O 2.250 bis unter 2.500 Euro
- O 2.500 bis unter 2.750 Euro
- O 2.750 bis unter 3.250 Euro
- O 3.250 bis unter 5.000 Euro
- O 5.000 bis unter 10.000 Euro
- O 10.000 Euro und mehr pro Monat

Fragebogen ausgefüllt am (heutiges Datum): [|] . [|] . [| | |]

Tag Monat Jahr

Bitte prüfen Sie Ihre Angaben noch einmal auf Vollständigkeit.

Wir danken Ihnen, für die Beantwortung des Fragebogens und Ihre beständige Unterstützung der GNN - Studie!

Impressum

Herausgeber: Deutsches Frühgeborenen-Netzwerk · Universität zu Lübeck
Klinik für Kinder- und Jugendmedizin
Ratzeburger Allee 160 · 23538 Lübeck

Druck: DRUCKHAUS MENNE GmbH · Rapsacker 10 · 23556 Lübeck

© Lübeck, 2014

Die Verwendung von Fragen aus der KiGGS-Studie erfolgt mit freundlicher Genehmigung des Robert Koch-Instituts.

Nachdruck, auch auszugsweise, nur mit schriftlicher Genehmigung des Herausgebers.
