# Supplementary material for: The circadian phase of antenatal glucocorticoid treatment affects the risk of behavioral disorders
Source: Nat Commun. 2020 Jul 17;11:3593. doi: 10.1038/s41467-020-17429-5 (PMC7367845; doi:10.1038/s41467-020-17429-5)
Supplement: Supplementary file 3 — Reporting Summary [file 41467_2020_17429_MOESM3_ESM.pdf]

nature research

Corresponding author(s): Prof. Dr. Henrik Oster, Dr Mariana Astiz

Last updated by author(s): May 7, 2020

Reporting Summary

Nature Research wishes to improve the reproducibility of the work that we publish. This form provides structure for consistency and transparency in reporting. For further information on Nature Research policies, see Authors & Referees and the Editorial Policy Checklist.

Statistics

For all statistical analyses, confirm that the following items are present in the figure legend, table legend, main text, or Methods section.

n/a	Confirmed	
☐	☒	The exact sample size (*n*) for each experimental group/condition, given as a discrete number and unit of measurement
☐	☒	A statement on whether measurements were taken from distinct samples or whether the same sample was measured repeatedly
☐	☒	The statistical test(s) used AND whether they are one- or two-sided<br>*Only common tests should be described solely by name; describe more complex techniques in the Methods section.*
☐	☒	A description of all covariates tested
☐	☒	A description of any assumptions or corrections, such as tests of normality and adjustment for multiple comparisons
☐	☒	A full description of the statistical parameters including central tendency (e.g. means) or other basic estimates (e.g. regression coefficient) AND variation (e.g. standard deviation) or associated estimates of uncertainty (e.g. confidence intervals)
☐	☒	For null hypothesis testing, the test statistic (e.g. *F*, *t*, *r*) with confidence intervals, effect sizes, degrees of freedom and *P* value noted<br>*Give P values as exact values whenever suitable.*
☒	☐	For Bayesian analysis, information on the choice of priors and Markov chain Monte Carlo settings
☒	☐	For hierarchical and complex designs, identification of the appropriate level for tests and full reporting of outcomes
☐	☒	Estimates of effect sizes (e.g. Cohen's *d*, Pearson's *r*), indicating how they were calculated

Our web collection on statistics for biologists contains articles on many of the points above.

Software and code

Policy information about availability of computer code

Data collection	Image Lab™ Software, Western blot scan, Bio-Rad, California, USA<br>Quantity One 1-D analysis software, ISH quantification, Bio-Rad, California, USA<br>CFX96 Real-Time PCR Detection System, qPCR data collection, Bio-Rad, California, USA<br>ClockLab software, Circadian data collection, Actimetrics, Evanston, IL, USA
Data analysis	Any-maze, Behavioral tests analysis, Any-Maze, Dublin, Ireland<br>ClockLab software, Circadian data analysis, Actimetrics, Evanston, IL, USA<br>G-power analysis software 3.1.9.1, sample size determination, University of Duesseldorf<br>GraphPad Prism 8, Statistic analysis, GraphPad software, California, USA<br>Jamovi 1.1.9.0, Statistic analysis, Topeka, KS, USA<br>CircWave v1.4, circadian rhythmicity analysis, University of Groningen<br>Image Lab™ Software, Western blot analysis, Bio-Rad, California, USA

For manuscripts utilizing custom algorithms or software that are central to the research but not yet described in published literature, software must be made available to editors/reviewers. We strongly encourage code deposition in a community repository (e.g. GitHub). See the Nature Research guidelines for submitting code & software for further information.

Data

Policy information about availability of data

All manuscripts must include a data availability statement. This statement should provide the following information, where applicable:

- Accession codes, unique identifiers, or web links for publicly available datasets
- A list of figures that have associated raw data
- A description of any restrictions on data availability

All raw data form human and mice experiments included in the main and supplementary figures and tables are fully available.

Field-specific reporting

Please select the one below that is the best fit for your research. If you are not sure, read the appropriate sections before making your selection.

☒ Life sciences ☐ Behavioural & social sciences ☐ Ecological, evolutionary & environmental sciences

For a reference copy of the document with all sections, see nature.com/documents/nr-reporting-summary-flat.pdf

Life sciences study design

All studies must disclose on these points even when the disclosure is negative.

Sample size	Sample sizes necessary for statistic accuracy was calculated using G-Power 3.1.9.1 software, University of Düsseldorf, Germany assuming 95% power and α=0.05.<br>Sample size used in each experiment is shown by individual data points and indicated in the figure legend and/or in the methods section.<br>Statistical tests used and level of significance are indicated for each figure at the end of the each legend.<br>Number of litters use for each experiment and for the overall project is detailed in the methods section.
Data exclusions	All data were included in the analysis
Replication	Behavioral experiments (circadian activity profile for CORT "out-of-phase" and CORT "in-phase" groups, elevated plus maze and forced swim test) were performed once per animal in two different batches with comparable outcomes. Data from both were combined for statistical analysis. Hormonal and protein levels and gene expression measurements in adults, embryos and mothers for all the genotypes were performed in duplicates in several different batches depending on the availability of offspring/pregnant mice. All data were comparable and pooled for statistical assessment. Thus, all the attempts at replication were successful.
Randomization	The pregnant mice were randomly allocated into the experimental groups. Adults offspring and fetus allocation depends on the maternal treatment. The adult offspring and the fetus were age- and weight- matched. A maximum of 2 male offspring per litter were include in each cohort for each of the following experiments:<br>1) Behavioral tests (EPM and FST)<br>2) Wheel-running experiments<br>3) Fecal collection for Corticoid measurement<br>4) DEX-suppression test<br>At the end, all offspring from each group were randomly assigned to each time-point.<br>All the experiments were run in male fetus, only pregnancies with at least 5 fetus were included.<br>Corticosterone measurements, gene expression and western blots were done on a maximum of 2 male fetus/mother to avoid litter effects and were randomly assigned to each measurement. In the case of experiments with small sample size (n=4) such as GR binding ability and western blots in Bmal1 +/+ and -/- (Fig 3 e and g and Fig 4 b and c) only one fetus/mother was included.<br>Similarly, for the analysis of the human data allocation of participants to in-phase and out-of-phase groups was done depending on the time of the maternal injection
Blinding	During data collection and analysis of behavioral experiments, hormonal and protein levels and gene expression analysis the treatment groups, time-points and genotypes were blinded to the investigator. Human data collection was blinded.

Reporting for specific materials, systems and methods

We require information from authors about some types of materials, experimental systems and methods used in many studies. Here, indicate whether each material, system or method listed is relevant to your study. If you are not sure if a list item applies to your research, read the appropriate section before selecting a response.

Materials & experimental systems

n/a	Involved in the study
☐	☒ Antibodies
☒	☐ Eukaryotic cell lines
☒	☐ Palaeontology
☐	☒ Animals and other organisms
☐	☒ Human research participants
☐	☒ Clinical data

Methods

n/a	Involved in the study
☒	☐ ChIP-seq
☒	☐ Flow cytometry
☒	☐ MRI-based neuroimaging

Antibodies

Antibodies used

Rabbit anti-GR, Dilution 1:1000, Ab183127, LOT# GR284781, Abcam, Cambridge, UK and was previously tested in rat tissue (1).
Rabbit anti-RevErbα, Dilution 1:1000, PA5-29865, LOT# UJ2853222B, Thermo Fisher Sci, Massachusetts, USA and was tested previously in mouse tissue (2).
Mouse anti-αTubulin, Dilution 1:1000, T5168, LOT# 039M4769V, Sigma-Aldrich, Missouri, USA and was successfully used in more than 1700 studies according to the manufacturer's website (e.g. 3).
Goat anti-Rabbit HRP, Dilution 1:20000, A9169, LOT# 117M4808V, Sigma-Aldrich, Missouri, USA
Horse anti-Mouse HRP, Dilution 1:3000, 7076S, LOT# 27 Cell Signaling, Massachusetts, USA

Validation

1. Hu S, Xia L, Luo H, Xu Y, Yu H, Xu D, Wang H., 2019. Prenatal caffeine exposure increases the susceptibility to non-alcoholic fatty liver disease in female offspring rats via activation of GR-C/EBP -SIRT1 pathway. Toxicology 417:23-34. doi: 10.1016/j.tox.2019.02.008)
2. Nam D, Chatterjee S, Yin H, Liu R, Lee J, Yechoor VK, Ma K. 2015. Novel Function of Rev-erbα in Promoting Brown Adipogenesis. Sci Rep. 5:11239. doi: 10.1038/srep11239.
3. Chang, Xu, Lin, Hsu, Hsieh-Li, Hwu, Liu, Lu, Sung. Survival Motor Neuron Protein Participates in Mouse Germ Cell Development and Spermatogonium Maintenance. Int J Mol Sci. 21(3):794. doi: 10.3390/ijms21030794.

Animals and other organisms

Policy information about studies involving animals; ARRIVE guidelines recommended for reporting animal research

Laboratory animals

Adult mice used for the experiments in Figure 1 and Supplementary Figure 1 were C57BL/6J males between 8 and 16 weeks old. C57BL/6J males and females (between 7-9 weeks old) were mated, females subject to gestational intervention and a maximum of 2 male fetus GD 15.5-16.5/mother were used for the experiments in Figure 2, 3 and Supplementary Figure 2, 3.

Hetrozygous (+/-) Bmal knock-out mice (B6.129S4(Cg)-Arntltm1Weit/J) (1) on a C57BL/6J background were used. Male and females (between 7-9 weeks old) were mated, females subject to gestational intervention and a maximum of 2 male fetus GD 15.5-16.5/mother were used for the experiments in Figure 3g, 4

Per1/2-doble mutants (B6.Cg-Per1tm1BrdTyrc-Brd/J & B6.Cg-Per2tm1Brd Tyrc-Brd/J) (2) on a C57BL/6J background were used. C57BL/6J wild-type male and Per1/2-double mutant females (between 7-9 weeks old) were mated, females subject to gestational intervention and a maximum of 2 male fetus GD 15.5-16.5/mother were used for the experiments in Supplementary Figure 4
Breedings were performed by the researchers in the animal facility of the University of Luebeck.

1. Storch KF; Paz C; Signorovitch J; Raviola E; Pawlyk B; Li T; Weitz CJ. 2007. Intrinsic circadian clock of the Mammalian retina: importance for retinal processing of visual information. Cell 130(4):730-41

2. Zheng B; Larkin DW; Albrecht U; Sun ZS; Sage M; Eichele G; Lee CC; Bradley A. 1999. The mPer2 gene encodes a functional component of the mammalian circadian clock. Nature 400(6740):169-73 / Zheng B, Albrecht U, Kaasik K, Sage M, Lu W, Vaishnav S, Li Q, Sun ZS, Eichele G, Bradley A, Lee CC. 2001. Nonredundant roles of the mPer1 and mPer2 genes in the mammalian circadian clock. Cell. 105(5):683-94.

Wild animals

No wild animals were used in the study

Field-collected samples

No field collected samples were used in the study

Ethics oversight

All experimental protocols were ethically approved by the Committee on Animal Health and Care of the Government of Schleswig-Holstein (V 242-7224.122-4(45-4/15) and V 242-7604/2017 (37-3/17) and were performed according to international guidelines on the ethical use of animals.

Note that full information on the approval of the study protocol must also be provided in the manuscript.

Human research participants

Policy information about studies involving human research participants

Population characteristics	Pre-term infants of a birth weight (BW) < 1,500 g and gestational age (GA) ≤ 36 6/7 weeks met the inclusion criteria. Exclusion criteria were lethal malformations (e.g., trisomy 13 or trisomy 18).
Recruitment	Within the study data were obtained from infants born between January 1st, 2009 and December 31st, 2013. At five years of age of GNN enrolled infants the parents were contacted and invited to a follow-up examination.
Ethics oversight	The German Neonatal Network (GNN; accessible web-link: https://www.vlbw.de/en) is a population-based observational study which is funded by the German Ministry of Education and Research (https://www.gesundheitsforschung-bmbf.de/de/deutsches-fruhgeborenen-netzwerk-german-neonatal-network-gnn-3798.php).

Note that full information on the approval of the study protocol must also be provided in the manuscript.

Clinical data

Policy information about clinical studies

All manuscripts should comply with the ICMJE guidelines for publication of clinical research and a completed CONSORT checklist must be included with all submissions.

Clinical trial registration	According to German research regulations the GNN had not to be registered as a clinical trial, however, it provides a platform for ISRCTN registered trials such as the AMV study (ISRCTN05025922; Göpel W et al. Lancet 2011; 378: 1627-34) and the NINSAPP trial (ISRCTN64011614; Kribs A et al. JAMA Pediatr 2015; 169: 723-30).
Study protocol	The study protocol can be accessed at https://www.vlbw.de/ (in German) - see also attached study protocol (in german and english) and informed consent form are provided as supplementary material
Data collection	For the purpose of this study, we retrospectively collected data on the timing of antenatal synthetic glucocorticoid injection (Betamethasone at least two injections 24 hrs appart of 8 or 12 mg) to the mother between weeks 24 and 34 of gestation in Lübeck, Cologne, and Essen. From a total of 141 preterm infants, exclusion criteria were applied to Dexamethasone treated children (6 cases) since the doses are given every 12 hrs, to children born before gestational week 24 (18 cases) and to children with incomplete or unreliable questionnaire (10 cases). Therefore, we correlated the time of GCs injection with the 5-year follow-up for the 107 preterm infants that met the criteria.<br>Parents received standardized questionnaires that had been used in the KiGGS, a German nation-wide health survey comprehensively mapping children's health (KiGGS: Kurth et al., BMC Public Health. 2008;8:196; Meyer, Eur Child Adolesc Psychiatry. 2017 Feb;26(2):165-175. GNN: Spiegler et al., Early Hum Dev 2017; 115:88-92). Parents provided a written informed consent to answer questions concerning detailed information on the children's social background, illnesses, general development and behavior. At follow-up examination at the local site a standardized assessment by the GNN study team (two study nurses, one pediatrician) was performed including monitoring of the questionnaire responses while the parents were present. All data were coded and entered into a central database.
Outcomes	The German Neonatal Network (GNN) studies the long term effects of genetic, clinical and social risk factors as well as the influence of center specific treatment strategies of infants born at a gestational age of< 37 weeks and with a birth weight of< 1500 g. A predefined clinical data set including antenatal and postnatal treatment and outcome data (250 parameters) was recorded prospectively on case report forms. The clinical data used in this study were pre-defined as follows:<br>Definitions:<br>Gestational age was calculated from the best obstetric estimate based on early prenatal ultrasound and obstetric examination. Small for gestational age was defined as birth weight percentile < 10 according to gestational age (Voigt M, Geburtsh. Frauenheilk. 66, 391-9 (2006)).<br>Birth weight was measured by a standardized scale for preterm infants at each participating site.<br>The mode of delivery was documented according to the attending obstetrician:<br>Planned (elective) cesarean section is defined as not immediate requirement of cesarean section >20 min after diagnosis of necessity of delivery. Emergency cesarean section is defined as the immediate requirement of cesarean section due to conditions that are associated with poor outcome (e.g. fetal distress, placenta praevia, placental abruption) and caesarean section within 20 min after diagnosis of necessity of delivery. Spontaneous delivery was defined as vaginal delivery.<br>Assessment: To assure proper assessment of clinical data a yearly on-site-monitoring by a study nurse or paediatrician experienced in perinatal medicine was performed. All clinical data were coded and entered into a central database.
