# Supplementary material for: The circadian phase of antenatal glucocorticoid treatment affects the risk of behavioral disorders
Source: Nat Commun. 2020 Jul 17;11:3593. doi: 10.1038/s41467-020-17429-5 (PMC7367845; doi:10.1038/s41467-020-17429-5)
Supplement: Supplementary file 2 — Peer Review File [file 41467_2020_17429_MOESM2_ESM.docx]

Peer Review Information

**Journal:** Nature Communications

**Manuscript title:** THE CIRCADIAN PHASE OF ANTENATAL GLUCOCORTICOID TREATMENT AFFECTS THE RISK OF BEHAVIORAL DISORDERS

**Corresponding author name(s):** Dr Mariana Astiz

**Editorial notes:**

**Transferred manuscripts**

This manuscript has been previously reviewed at another journal that is not operating a transparent peer review scheme. This document only contains reviewer comments and rebuttal letters for versions considered at *Nature Communications*.

**Reviewer comments & decisions:**

**Reviewer comments, first version:**

Reviewer #1 (Remarks to the Author):

The authors have provided very thoughtful responses to all of my comments and included additional discussions where relevant to highlight some of the limitations of their study design while maintaining appropriate discussion of the translational relevance of their results. Of particularly importance is the expansion of the number of children recruited allowing them to test for various confounders. The newly added ISH image from the PVN is also an important addition to the manuscript although I would recommend that the authors provide a larger image focused more on the PVN rather than an entire brain section.


Reviewer #2 (Remarks to the Author):

This is an exciting paper as it suggests that the time of administration (evening or morning) of antenatal corticosteroid administration, given to women at risk of premature labour, determines whether there is an adverse effect on the health of offspring, or not. This could lead to a major impact on clinical practice, to ensure that the necessary antenatal corticosteroids that can save premature babies’ lives do not have detrimental, long lasting effects on babies that are delivered at term.

The authors have carried out a series of well-described, logical experiments demonstrating
that it is the timing of the corticosteroid treatment rather than the dose that is crucial for
causing the long-lasting, adverse effects on the behavior of the offspring.

The revised paper is considerably improved, and I now do not have any concerns in the animal studies which is the main part of the manuscript. The data is presented and analysed appropriately and fully support the conclusions drawn.

However, the ‘observational’ study in humans, to support that timing of glucocorticoid treatment in women at risk of premature birth, can affect the behavioural response to stress (poorer stress compensatory capacity) in 5-year olds, still has a limitation in respect to the size of the study.
While the authors have now added data from a new Centre, the numbers in the groups are still very small (n=33 for the in phase Tx group and n=20 for the out of phase group). The ‘middle’ group (n=54) was not included in the analysis. The behavioural analysis score of the children was calculated from 5 questions and the answers to these questions are subjective, so such small groups in this analysis is not too compelling. However, a significant effect was obtained in the overall behavioural score (but not this is not seen in the individual behaviours).

This is an intriguing hypothesis that could revolutionise prenatal care for women at risk of prenatal birth, and the publication of this study would demonstrate the crucial need for further studies investigating the consequences of the timing of antenatal steroid treatment on the behavioural outcomes of the babies and children.


Reviewer #3 (Remarks to the Author):

I supported the publication of this manuscript in its previous venue as well, with some modifications. I think that it is an important study, and do not agree with Reviewer 1 that the molecular results are "not impressive". While they are not extensive, they certainly support well the hypotheses of the authors, and the conclusions are now all quite well supported.

The authors have answered all my comments in satisfactory fashion. I also think that they have answered the other reviewers.

**Author rebuttal, first version:**

Reviewer #1 (Remarks to the Author):

The authors have provided very thoughtful responses to all of my comments and included additional discussions where relevant to highlight some of the limitations of their study design while maintaining appropriate discussion of the translational relevance of their results. Of particularly importance is the expansion of the number of children recruited allowing them to test for various confounders. The newly added ISH image from the PVN is also an important addition to the manuscript although I would recommend that the authors provide a larger image focused more on the PVN rather than an entire brain section.

**Action:** We would like to thank reviewer #1 for the time invested on revising our paper and all his/her constructive comments that have undoubtedly improved it.

Reviewer #2 (Remarks to the Author):

This is an exciting paper as it suggests that the time of administration (evening or morning) of antenatal corticosteroid administration, given to women at risk of premature labour, determines whether there is an adverse effect on the health of offspring, or not. This could lead to a major impact on clinical practice, to ensure that the necessary antenatal corticosteroids that can save premature babies’ lives do not have detrimental, long lasting effects on babies that are delivered at term.

The authors have carried out a series of well-described, logical experiments demonstrating
that it is the timing of the corticosteroid treatment rather than the dose that is crucial for
causing the long-lasting, adverse effects on the behavior of the offspring.

The revised paper is considerably improved, and I now do not have any concerns in the animal studies which is the main part of the manuscript. The data is presented and analysed appropriately and fully support the conclusions drawn.

However, the ‘observational’ study in humans, to support that timing of glucocorticoid treatment in women at risk of premature birth, can affect the behavioural response to stress (poorer stress compensatory capacity) in 5-year olds, still has a limitation in respect to the size of the study.
While the authors have now added data from a new Centre, the numbers in the groups are still very small (n=33 for the in phase Tx group and n=20 for the out of phase group). The ‘middle’ group (n=54) was not included in the analysis. The behavioural analysis score of the children was calculated from 5 questions and the answers to these questions are subjective, so such small groups in this analysis is not too compelling. However, a significant effect was obtained in the overall behavioural score (but not this is not seen in the individual behaviours).

This is an intriguing hypothesis that could revolutionise prenatal care for women at risk of prenatal birth, and the publication of this study would demonstrate the crucial need for further studies investigating the consequences of the timing of antenatal steroid treatment on the behavioural outcomes of the babies and children.

**Action:** We would like to thank reviewer #2 for the time invested on revising our paper and all his/her constructive comments.

We agree that our observational study has limitations with regard to the cohort size and the specificity of the behavioral assessment. However, we are confident that its publication may encourage more systematic data collections including the time of antenatal steroid treatment and more specific stress-related tests during the 5-year follow-up.

Reviewer #3 (Remarks to the Author):

I supported the publication of this manuscript in its previous venue as well, with some modifications. I think that it is an important study, and do not agree with Reviewer 1 that the molecular results are "not impressive". While they are not extensive, they certainly support well the hypotheses of the authors, and the conclusions are now all quite well supported.

The authors have answered all my comments in satisfactory fashion. I also think that they have answered the other reviewer

**Action:** We would like to thank reviewer #3 for the time invested on revising our paper and all his/her constructive comments and support.

**Reviewer comments, second version:**

**Author rebuttal, second version:**

**Reviewer comments, third version:**

**Author rebuttal, third version:**
